# Newer human inosine 5′-monophosphate dehydrogenase 2 (*h*IMPDH2) inhibitors as potential anticancer agents

**DOI:** 10.1080/14756366.2018.1474211

**Published:** 2018-05-24

**Authors:** Chetan P. Shah, Prashant S. Kharkar

**Affiliations:** Department of Pharmaceutical Chemistry, SPP School of Pharmacy and Technology Management, SVKM’s NMIMS, Mumbai, India

**Keywords:** Mycophenolic acid, *h*IMPDH2 inhibitors, anticancer agents, MTT, *de novo* purine synthesis

## Abstract

Human inosine 5′-monophosphate dehydrogenase 2 (*h*IMPDH2), being an age-old target, has attracted attention recently for anticancer drug development. Mycophenolic acid (MPA), a well-known immunosuppressant drug, was used a lead structure to design and develop modestly potent and selective analogues. The steep structure–activity relationship (SAR) requirements of the lead molecule left little scope to synthesise newer analogues. Here, newer MPA amides were designed, synthesised and evaluated for *h*IMPDH2 inhibition and cellular efficacy in breast, prostate and glioblastoma cell lines. Few title compounds exhibited cellular activity profile better than MPA itself. The observed differences in the overall biological profile could be attributed to improved structural and physicochemical properties of the analogues over MPA. This is the first report of the activity of MPA derivatives in glioblastoma, the most aggressive brain cancer.

## Introduction

Cancer is a group of heterogeneous diseases characterised by uncontrolled growth and spread of abnormal cells. Cancer cells adapt to their high metabolic state by increasing energy production, that is increased cellular metabolism. Potential molecular targets for cancer therapeutics are signalling pathways that are preferentially activated in numerous cancers. However, heterogeneity of tumours and frequent oncogenic mutations over a period makes it difficult for a medicinal chemist to design antitumour molecules that target tumour-specific pathways^1^. Proliferating cells have a high demand for guanine nucleotides (GMP) that generally cannot be sustained by salvage pathways, which explains the importance of inosine 5'-monophosphate dehydrogenase (IMPDH, an enzyme linked with proliferation and malignancy). Non-proliferating cells use an alternative purine nucleotide synthesis pathway, the salvage pathway, to synthesise GMP. These observations pose IMPDH as a potential target to suppress tumour cell growth^1^.

The IMPDH (E.C.1.1.1.205), the nicotinamide adenine dinucleotide (NAD^+^)-dependent enzyme that controls *de novo* synthesis of purine nucleotides, catalyses the oxidation of inosine 5'-monophosphate (IMP) to xanthosine 5'-monophosphate (XMP), which is then converted to guanosine 5'-monophosphate (GMP) by GMP synthase. The IMP also serves as a substrate for the biosynthesis of adenosine 5'-monophosphate (AMP). An adequate pool of purine nucleotides is essential for cell proliferation, cell signalling and as an energy source^2^. Consequently, inhibition of IMPDH causes a variety of biological responses, such as reduction in guanine nucleotide pools resulting in arrest of cell proliferation (interruption of DNA and RNA synthesis)^3^, a decline in intracellular signalling (G-protein-mediated signal transduction)^4–6^, downregulation of *c-myc* and *Ki-ras* oncogenes *in vitro*^7^. Also, IMPDH inhibition is associated with an upregulation of *p53* (commonly mutated protein in human cancers)^8^, *p21*, *bax* and a downregulation of *bcl-2*, survivin and *p27* protein^9^.

The enzyme human IMPDH exists in two isoforms (type 1 and type 2). These isoforms are of identical size and share 84% sequence identity. However, the type 1 “housekeeping” isoform is constitutively expressed in both normal and neoplastic cells, while type 2 expression is preferentially upregulated in human neoplastic cell lines^10^. Human IMPDH type 1 (*h*IMPDH1) has been identified as an anti-angiogenic drug target and mycophenolic acid (MPA) was found to block tumour-induced angiogenesis (*in vivo*)^11^ while the disproportionate increase in human IMPDH type 2 (*h*IMPDH2) activity in neoplastic cells has made this isoform a key target for the development of anticancer drug discovery^12^^,^^13^. Also, *h*IMPDH2 has become a major drug target for immunosuppression^14^^,^^15^, antiviral^16^ and parasitic infestations^17–20^.

**Figure 1.**
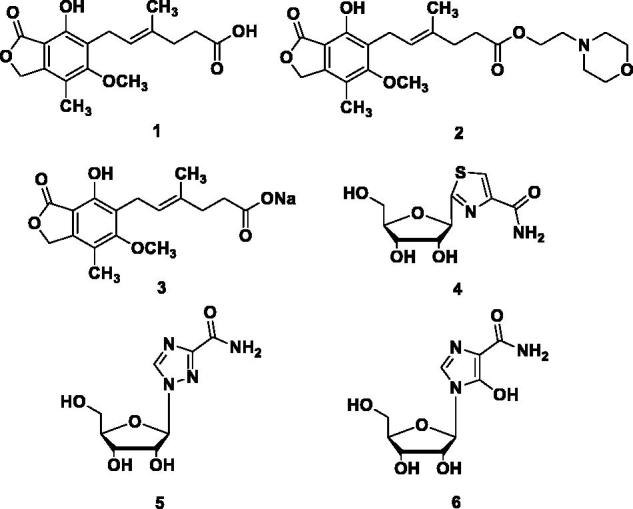
Currently used IMPDH inhibitor drugs.

Mycophenolic acid (MPA) **1** (Figure 1), a natural product, is a reversible, potent, uncompetitive inhibitor of IMPDH and known to be an anticancer and immunosuppressive agent. Mycophenolate mofetil (MMF, **2**, a prodrug of MPA), has been approved for the treatment of acute allograft rejection following kidney transplant. The MPA and its related forms MMF or MPA sodium (**3**) (MPS) cause dose-limiting gastrointestinal (GI) toxicity. However, adverse effects related to the treatment with MPA-based drugs, such as diarrhoea, leukopenia, sepsis and vomiting, are the barriers to the administration of higher doses and more effective treatment^21^. The competitive IMPDH inhibitors such as tiazofurin (**4**), ribavirin (**5**) and mizoribine (**6**) (after intracellular activation by phosphorylation) are nucleoside analogues and derivatives thereof^22^ have unfavourable tolerability profiles too. Thus, there is a urgent need for newer, safer, potent and orally bioavailable IMPDH inhibitors^23^^,^^24^.

**Figure 2.**
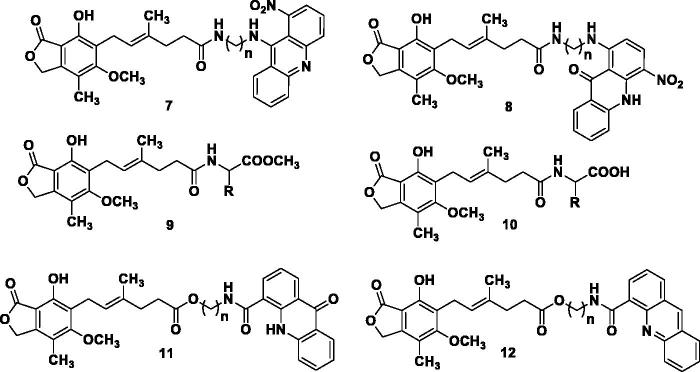
Recently reported MPA analogues.

In an attempt to address this issue, significant efforts were focused on bioisosteric replacements along with various structural modifications of **1** to develop potent IMPDH inhibitors, but with limited success^12^^,^^25^. The MPA has proven to be an effective inducer of differentiation in a number of cancer cell lines (melanoma, leukaemia and prostate cancer). Also, MPA exhibited synergism with imatinib in the treatment of chronic myelogenous leukaemia (CML)^26^. Furthermore, antitumour activities of several derivatives of **1** are reported^27^^,^^28^. The MPA conjugates possessing amide and ester linkages (**7–12**, Figure 2) exhibited potent anticancer properties^21^^,^^29–31^. These findings strongly support the role of **1** as a potential anticancer drug. Moreover, MPA, being an acid, is likely to be prevented from entering in the central nervous system (CNS). There are no evidence in the literature citing this fact that **1** could be useful for treating gliomas, cancers of the CNS^32^.

In the present work, we report the design and synthesis of newer analogues of **1** with the aim of improving its cellular potency, selectivity and toxicity profiles. As is previously known, little structural alteration of **1** was detrimental to its IMPDH inhibitory activity^29–32^. It was crucial to select the design strategy leading to retention of the inhibitory potential against *h*IMPDH2. The title compounds were further evaluated for their cellular potency in MDA-MB-231 (breast adenocarcinoma), DU145 (prostate carcinoma) and U87 MG (glioblastoma astrocytoma) cell lines. The present study summarises the finding of the medicinal chemistry activities centred on **1**.

## Methods

### Chemistry

The synthetic details of only two representative compounds are described. For the rest of compounds, readers are requested to refer to the Supplementary Material section. We preferentially selected aliphatic amines to balance logP values, particularly benzyl amines and while avoiding the use of anilines which are often mutagenic. Nonetheless, two compounds with substituted anilines were synthesised as a part of structure–activity relationship (SAR) studies.

**Scheme 1.**
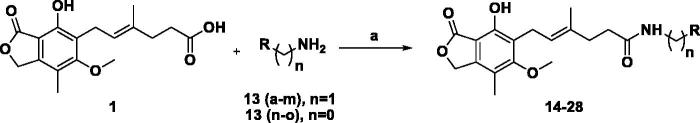
General scheme for synthesis of mycophenolic acid amides (**14–28**): Reagents and conditions: Method A: DMAP, EDCI.HCl, DMF, 0** **°C 6 h, RT, 48–72 h; Method B: DIPEA, HATU, DMF, 0** **°C 6** **h, RT, 48–72 h.

*General procedure for the synthesis of MPA amides****(14–28)***^31^^,^^33^ (Scheme 1). *Method A:* Mycophenolic acid **1** (1 eq.), appropriate amines **13a–m** (1.14 eq.) and 4(dimethylamino)pyridine (DMAP) (1.14 eq.) were dissolved in anhydrous DMF (3 ml). The reaction mixture was cooled to 0 °C in an ice bath, followed by the addition of EDCI.HCl (1.1 eq.) with stirring. The reaction mixture was stirred at 0 °C for 6 h and left at RT for 48–72 h. After completion of the reaction, the reaction mixture was cooled to 5 °C and poured into cold water (25 ml), extracted with EtOAc (3X20 ml). The combined organic layers were dried over Na_2_SO_4_ (anhydrous) and concentrated *in vacuo*. The crude products were purified with the help of column chromatography using DCM:EtOAc (9:1) as the mobile phase.

*Method B:* Mycophenolic acid **1** (1 eq.), appropriate amines **13n-o** (0.67 eq.) and HATU (1.33 eq.) were dissolved in anhydrous DMF (3 ml). The reaction mixture was cooled to 0 °C in an ice bath and DIPEA (3.4 eq.) was added with stirring. The reaction mixture was stirred at 0 °C for 6 h and left at RT for 48–72 h. After completion of the reaction, the contents of the flask were cooled to 5 °C and poured into cold water (25 ml), extracted with EtOAc (3X20 ml). The combined organic layers were dried over Na_2_SO_4_ (anhydrous) and concentrated *in vacuo*. The crude products were purified with the help of column chromatography using DCM:EtOAc (9:1) as the mobile phase.

*(S)(E)-6–(4-hydroxy-6-methoxy-7-methyl-3-oxo-1,3-dihydroisobenzofuran-5-yl)-4-methyl-N-(1-phenylethyl)hex-4-enamide****(14)***. It was synthesised using **1** (0.1 g, 0.31 mmol), (*S*)-(−)-*α*-methylbenzylamine **13a** (0.045 ml, 0.35 mmol), DMAP (0.044 g, 0.35 mmol) and EDCI.HCl (0.066 g, 0.34 mmol) as per Method A to yield **14** as off-white solid. Yield: 55%; TLC: R*_f_* = 0.71 (DCM: EtOAc, 8:2); purity (HPLC): 95.28%; mp: 114–116 °C; IR (KBr) cm^−1^ 3444 (OH, str), 3284 (NH, str), 1745 (O–C=O, str), 1637 (NH-C=O, str), 1551 (C=C, str), 1134 (C-O, str); ^1^H-NMR (DMSO-d_6,_ D_2_O exchange, 400 MHz) *δ* 7.29–7.12 (m, 5H), 5.21 (s, 2H), 5.09–5.12 (t, *J* = 7.0 Hz, 1H), 4.79 (q, *J* = 7.0 Hz, 1H), 3.63 (s, 3H), 3.25 (d, *J* = 6.8 Hz, 2H), 2.14 (dd, *J* = 7.1, 11.9 Hz, 4H), 2.04 (s, 3H), 1.69 (s, 3H), 1.23 (d, *J* = 7.0 Hz, 3H); MS (ESI) *m/z*: 422 [M–H]^−^.

*(E)-6–(4-hydroxy-6-methoxy-7-methyl-3-oxo-1,3-dihydroisobenzofuran-5-yl)-N-(3-methoxyphenyl)-4-methylhex-4-enamide****(27)***. It was synthesised using **1** (0.1 g, 0.31 mmol), 3-methoxyaniline **13n** (0.024 ml, 0.21 mmol), HATU (0.158 g, 0.41 mmol) and DIPEA (0.185 ml, 1.06 mmol) as per Method B to yield **27** as off-white solid. Yield: 45%; TLC: R*_f_* = 0.62 (DCM:EtOAc, 8:2); purity (HPLC): 97.63%; mp: 238–240 °C; IR (KBr) cm^−1^ 3437 (OH, str), 1756 (O–C=O, str), 1689 (NH–C=O, str), 1613 (C=C, str), 1127 (C–O, str); ^1^H-NMR (DMSO-d_6,_ D_2_O exchange, 400 MHz) *δ* 7.2 (s, 1H), 6.8–6.6 (s, 3H), 5.21 (s, 2H), 5.15–5.13 (t, *J* = 7.0 Hz, 1H), 3.69 (s, 3H), 3.64 (s, 3H), 3.28 (d, *J* = 6.8 Hz, 2H), 2.30 (t, *J* = 5.8, 9.5 Hz, 2H), 2.22 (t, *J* = 7.8 Hz, 2H), 2.04 (s, 3H), 1.76 (s, 3H); MS (ESI) *m/z*: 424 [M–H]^−^.

### Biological activity

#### *In vitro h*IMPDH2 inhibition assay^23^

The enzyme (*h*IMPDH2) was purchased from NovoCIB SAS (Lyon, France). A total of 15 molecules were screened at 10 μM concentration for enzyme inhibition and IC_50_ values were determined for compounds with *h*IMPDH2 inhibition >70% at 10 μM. The assay was performed in a 200 µl final volume in 96-well UV plates (Tarsons, 980040, Tarsons Products Pvt. Ltd., Kolkata, India) with a reaction buffer composed of 100 mM Tris–HCl (pH 8.6), 100 mM KCl and 5 mM DTT, 4% v/v DMSO plus or minus test compound and 0.15 mU of purified *h*IMPDH2 enzyme per well (from 1.5 mg/ml stock solution). The final volume of the enzyme stock solution per well was 2 µl which was insignificant to cause any change in the final assay buffer composition. The reaction was initiated by the addition of (substrate buffer) 0.2 mM of IMP and 0.2 mM of NAD^+^ and the assay was allowed to proceed at 37 °C for 45 min. The generated NADH was measured by reading the absorbance at 340 nm. At this wavelength, a background of <0.1 optical density (OD) was observed with negligible crosstalk between wells. The MPA (10 µM) was used as a positive control and DMSO as a vehicle control. For IC_50_ determinations, a total of 10 concentrations ranging from 25 µM to 50 nM in triplicates were used. Enzyme inhibition and IC_50_ values were expressed in % inhibition and μM, respectively.

#### Cell viability (MTT assay)^34^

Cancer cell lines such as MDA-MB-231 (breast adenocarcinoma), DU145 (prostate carcinoma) and U87 MG (glioblastoma astrocytoma) were purchased from National Centre for Cell Sciences (NCCS) (Pune, India). Cytotoxic activity of compounds (>80% *h*IMPDH2 inhibition at 10 μM) was evaluated using colorimetric MTT assay on the above-mentioned cell lines. Briefly, cells were grown in DMEM media supplemented with foetal bovine serum (FBS) 10% and penicillin–streptomycin (50 U/ml, 50 µg/ml) at 37 °C, CO_2_ (5%) and air (95%). Logarithmically, growing cells were seeded using 96-well plate at different concentrations of the test compounds ranging from 0.01 to 100 μM (seeding density: MDA-MB-231: 10,000 cells/well, DU145: 8000 cells/well, U87 MG: 5000 cells/well). After 24 h of seeding, the cells were observed under microscope and treated with varying drug concentration along with DMSO (vehicle control). Each dilution of test compound was added in triplicate. Following 48-h incubation with the compounds, cells were incubated with MTT reagent (5 mg/ml) for 4 h, and then 100 μl of DMSO was added to dissolve formazan crystals. The absorbance was then measured at 540 nm and 630 nm (background scan) using EPOCH 2 BioTek microplate reader. The IC_50_ values for the tested compounds were calculated and expressed in μM using mean of triplicate readings. IC_50_ is defined as the compound concentration required to reduce the viability of the cells by 50% with respect to the control.

## Results and discussion

### Chemistry

The best operative method for drug discovery is to modify drug substances with known biological activity. Novel analogues are synthesised using molecular modifications to alter the physicochemical properties which may lead to enhancement in efficacy or receptor interactions, administration pathway, toxicity and stability issues. The MPA (**1**) is a well-known *h*IMPDH2 inhibitor with several pharmacological activities^2^. Its antitumour activity was abolished or reduced when any of the modifications, for example reduction or oxidation of the olefin, demethylation of –OCH_3_, oxidation of methyl group attached to the benzene substructure and altering of the terpene side chain, were made^28^. We set out with the aim of synthesising MPA amides since the amides were spared from the reduction or loss of biological activity on its structural modification(s). We carefully selected the amines to be coupled with MPA –COOH group based on the predicted structural, pharmacokinetic and toxicity properties of the proposed compounds (Table S1, Supplementary Material section).

The MPA possesses electrophilic centre at the acyl carbon in the lactone ring and free phenol group. Protection of the phenolic –OH using Ac_2_O (mycophenolic acid acetate), *tert*-butyldimethylsilyl triflate or *tert*-butyldimethylsilyl chloride (7-O-TBDMS-mycophenolic acid) and selective hydrolysis of the same has been reported and used for amide formation^21^. Protection and deprotection approach increases the number of steps leading to lower overall yield. Many reported coupling agents (isobutyl chloroformate, diphenyl phosphorazidate (DPPA)/triethylamine (TEA), 2-ethoxy-1-ethoxycarbonyl-1,2-dihydroquinoline (EEDQ)/pyridine, O-(Benzotriazol-1-yl)-N,N,N′,N′-tetramethyluronium hexafluorophosphate (HBTU)/N-methylmorpholine (NMM), O-(Benzotriazol-1-yl)-N,N,N′,N′-tetramethyluronium tetrafluoroborate (TBTU)/1-hydroxybenzotriazole (HOBt), 1-ethyl-3–(3′-dimethylaminopropyl)carbodiimide (EDCI)/DMAP in the presence of HOBt and so on) were used to form an amide bond between the –COOH of **1** and various amines **13(a-o)** (Scheme 1) selectively, without protection of the phenolic –OH^30^^,^^31^. Unfortunately, none of the attempted methods led to the proposed compounds due to low conversion of the substrates, problems with purification and similar reasons. The reaction conditions using EDCI/DMAP without HOBt (Method A) and HATU/DIPEA (Method B) were proved to be suitable for the synthesis of MPA derivatives **14–28** in moderate yields 40–75% (Scheme 1) and higher purity of the products.

### Biological activity

#### *In vitro h*IMPDH2 inhibition assay

**Figure 3.**
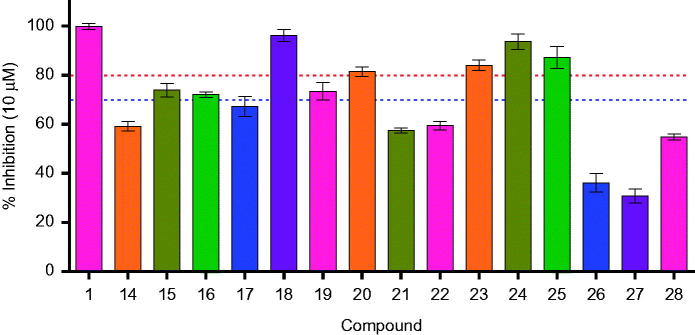
*h*IMPDH2 % inhibition of compounds **14–28**.

In line with our previous efforts on *h*IMPDH2 inhibitor discovery and development, all the synthesised molecules were screened for *h*IMPDH2 inhibition at 10 μM, in triplicate. The results are shown in Table 1. A total of eight derivatives (**15**, **16**, **18–21**, **24** and **25)** exhibited *h*IMPDH2 inhibition >70% (subjected to IC_50_ determination), while remaining compounds showed % inhibition <70. None of the synthesised molecules exhibited *h*IMPDH2 inhibitory activity superior than MPA (**1**) (IC_50_ = 0.25 ± 0.03 μM), although **18** (IC_50_ = 0.33 ± 0.11 μM) and **24** (IC_50_ = 0.48 ± 0.02 μM) were close competitors (Table 1, Figure 3). This could be possibly due to the stringent structural requirements and receptor binding space of the enzyme^27^^,^^28^.

**Table 1.** *h*IMPDH2 enzyme inhibition of MPA amides (**14–28** at 10 µM).

Compound	R	*h*IMPDH2 enzyme	
		% Inhibition^a^	IC_50_ (µM)**** ****±**** ****SD^b^
**1**	Mycophenolic acid	99.90 **±** 1.20	0.25 ± 0.03
**14**	(*S*)-(−)-α-Methylbenzyl	59.15 **±** 1.91	n.d.^c^
**15**	(*R*)-(+)-α-Methylbenzyl	73.94 **±** 2.84	0.82 ± 0.32
**16**	2-Chlorobenzyl	72.04 **±** 1.11	0.73 ± 0.06
**17**	2-Furfuryl	67.18 **±** 4.16	n.d.^c^
**18**	4-Methoxybenzyl	96.13 **±** 2.51	0.33 ± 0.11
**19**	4-Methylbenzyl	73.42 **±** 3.62	0.60 ± 0.13
**20**	4-Chlorobenzyl	81.36 **±** 1.95	0.57 ± 0.11
**21**	4-Pyridylmethyl	57.38 **±** 1.02	n.d.^c^
**22**	2-Pyridylmethyl	59.38 **±** 1.81	n.d.^c^
**23**	2-Methylbenzyl	84.00 **±** 2.13	0.50 ± 0.18
**24**	4-Phenylbutyl	93.62 **±** 3.17	0.48 ± 0.02
**25**	3,4-(Methylenedioxy)benzyl	87.09 **±** 4.54	0.51 ± 0.08
**26**	2-Morpholinoethyl	36.05 **±** 3.86	n.d.^c^
**27**	3-Methoxyphenyl	30.82 **±** 2.82	n.d.^c^
**28**	4-Methoxyphenyl	54.84 **±** 1.15	n.d.^c^

^a^All the data are expressed as ± SD (results are average of duplicate analysis).

^b^All the data are expressed as ± SD (results are average of triplicate analysis). IC_50_ value was determined when the inhibitory rate of compound is higher than 70% at the concentration of 10 µM.

^c^n.d.: not determined.

##### Cell viability (MTT assay)

**Figure 4.**
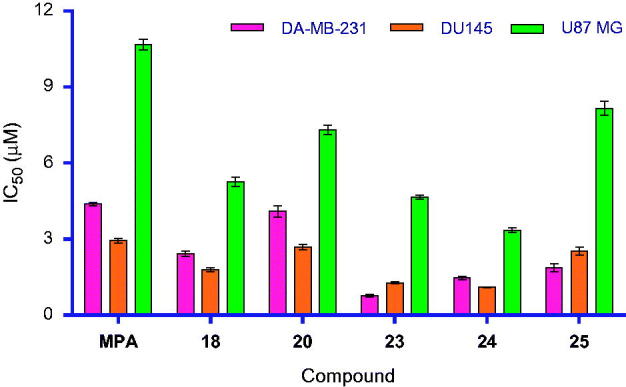
Cytotoxicity of MPA amide derivatives.

Cytotoxic activity of six compounds (**MPA**, **18**, **20** and **23–25**) are reported in Table 2. All MPA amides exhibited better activity than MPA in cell-based assays. This may be due to lower logP value (2.68) of MPA (free acid group), whereas the derivatives tested possessed higher logP (average logP 3.98). Out of five compounds screened (Table 2) against three cell lines, **23** was found to be more potent compared to **18**, **20**, **24**, **25** and five-fold more potent than MPA in MDA-MB-231 (breast adenocarcinoma) cell line (Table 2, Figure 4). Similarly, **24** exhibited greater potency than **18**, **20**, **23**, **25** and three-fold higher potency than MPA in DU145 (prostate carcinoma) cell line (Table 2, Figure 4). For U87 MG, **24** showed promising activity compared to **18**, **20**, **23**, **25** and three-fold more active than MPA in U87 MG (glioblastoma astrocytoma) cell line (Table 2, Figure 4). This is the first time anticancer activity of MPA derivatives on glioblastoma cell lines was demonstrated. Higher lipophilicity and masking of the –COOH in **1** could possibly be responsible for this observation since usually acids are prevented from entering in the CNS.^35^ Also, compound **24** and **1** were tested for hPBMC proliferation assay (see Supplementary Material section for protocol) and both these compounds exhibited similar % cell viability (63% for **1** and 65% for **24)**. The possibility of the title compounds behaving as prodrugs could be minute due to the difficulty in hydrolysing the amide linkage inside cells and release of the active form “MPA”. The higher cellular potency of these derivatives over MPA is definitely a progress towards the goal, that is to discover a novel anticancer agent, better than MPA.

**Table 2.** Cytotoxicity of MPA amide derivatives

S. No.	Compound	Cell lines [IC_50_ (μM) ± SD^a^]		
		MDA-MB-231^b^	DU145^c^	U87 MG^d^
1	Cisplatin	50.40 ± 1.1	34.47 ± 0.78	21.32 ± 0.39
2	Doxorubicin HCl	0.54 ± 0.08	0.21 ± 0.06	0.11 ± 0.02
3	MPA **(1)**	4.38 ± 0.06	2.94 ± 0.09	10.69 ± 0.21
4	**18**	2.42 ± 0.11	1.8 ± 0.07	5.26 ± 0.18
5	**20**	4.10 ± 0.23	2.69 ± 0.11	7.31 ± 0.17
6	**23**	**0.77 ± 0.04**	1.28 ± 0.04	4.66 ± 0.07
7	**24**	1.46 ± 0.07	**1.09 ± 0.02**	**3.35 ± 0.09**
8	**25**	1.86 ± 0.16	2.53 ± 0.15	8.16 ± 0.27

^a^All the data are expressed as ± SD (results are average of triplicate analysis).

^b^MDA-MB-231 (breast adenocarcinoma).

^c^DU145 (prostate carcinoma).

^d^U87 MG (glioblastoma astrocytoma).

Bold values indicate the most potent molecule in each cell line.

## Conclusions

Our design strategy based on MPA as the lead structure yielded fruitful outcome in terms of slightly better *h*IMPDH2 inhibitors with improved cellular potency. The potential issues with the steep SAR exhibited by the lead molecule in cells were overcome by virtually modulating the physicochemical properties and then carefully cheery-picking the analogues to be synthesised. Our medicinal chemistry approach yielded modestly more potent molecules in cells. The lead molecules are further being evaluated for their utility as potential anticancer agents.

## Supplementary Material

Supplemental Material
