# Supplementary material for: Newer human inosine 5′-monophosphate dehydrogenase 2 (hIMPDH2) inhibitors as potential anticancer agents
Source: J Enzyme Inhib Med Chem. 2018 May 24;33(1):972–7. doi: 10.1080/14756366.2018.1474211 (PMC6009919; doi:10.1080/14756366.2018.1474211)
Supplement: Supplemental Material [file IENZ_A_1474211_SM4762.pdf]

**Supporting Information**

# Mycophenolic acid-amides derivatives as human Inosine-5′-Monophosphate dehydrogenase 2 inhibitors (*h*IMPDH2)

Chetan P. Shah and Prashant S. Kharkar*

*Department of Pharmaceutical Chemistry, SPP School of Pharmacy and Technology Management, SVKM's NMIMS, V. L. Mehta Road, Vile Parle (West),Mumbai, India*

* Corresponding Author: Prashant S. Kharkar, prashant.kharkar@nmims.edu, Department of Pharmaceutical Chemistry, SPP School of Pharmacy and Technology Management, SVKM's NMIMS, V. L. Mehta Road,Vile Parle (West), Mumbai-400056. India.

## Table of Contents


| Sr. No. | Description | Page No. |
|---|---|---|
| Table 1S | Summary of the physicochemical and calculated molecular properties of the synthesized molecules | 1-2 |
| - | General | 3-9 |
| Figure 1S | $^1$H-NMR ($D_2O$ exchg.) spectrum of compound **14** | 10 |
| Figure 2S | Mass spectrum of compound **14** | 10 |
| Figure 3S | HPLC chromatogram of compound **14** | 11 |
| Figure 4S | FTIR spectrum of compound **14** | 11 |
| Figure 5S | $^1$H-NMR ($D_2O$ exchg.) spectrum of compound **15** | 12 |
| Figure 6S | Mass spectrum of compound **15** | 12 |
| Figure 7S | HPLC chromatogram of compound **15** | 13 |
| Figure 8S | FTIR spectrum of compound **15** | 13 |
| Figure 9S | $^1$H-NMR ($D_2O$ exchg.) spectrum of compound **16** | 14 |
| Figure 10S | Mass spectrum of compound **16** | 14 |
| Figure 11S | HPLC chromatogram of compound **16** | 15 |
| Figure 12S | FTIR spectrum of compound **16** | 15 |
| Figure 13S | $^1$H-NMR ($D_2O$ exchg.) spectrum of compound **17** | 16 |
| Figure 14S | Mass spectrum of compound **17** | 16 |
| Figure 15S | HPLC chromatogram of compound **17** | 17 |
| Figure 16S | FTIR spectrum of compound **17** | 17 |
| Figure 17S | $^1$H-NMR ($D_2O$ exchg.) spectrum of compound **18** | 18 |
| Figure 18S | Mass spectrum of compound **18** | 18 |
| Figure 19S | HPLC chromatogram of compound **18** | 19 |
| Figure 20S | FTIR spectrum of compound **18** | 19 |
| Figure 21S | $^1$H-NMR ($D_2O$ exchg.) spectrum of compound **19** | 20 |
| Figure 22S | Mass spectrum of compound **19** | 20 |
| Figure 23S | HPLC chromatogram of compound **19** | 21 |
| Figure 24S | FTIR spectrum of compound **19** | 21 |
| Figure 25S | $^1$H-NMR ($D_2O$ exchg.) spectrum of compound **20** | 22 |
| Figure 26S | Mass spectrum of compound **20** | 22 |
| Figure 27S | HPLC chromatogram of compound **20** | 23 |
| Figure 28S | FTIR spectrum of compound **20** | 23 |
| Figure 29S | $^1$H-NMR ($D_2O$ exchg.) spectrum of compound **21** | 24 |
| Figure 30S | Mass spectrum of compound **21** | 24 |
| Figure 31S | HPLC chromatogram of compound **21** | 25 |
| Figure 32S | FTIR spectrum of compound **21** | 25 |
| Figure 33S | $^1$H-NMR ($D_2O$ exchg.) spectrum of compound **22** | 26 |
| Figure 34S | Mass spectrum of compound **22** | 26 |
| Figure 35S | HPLC chromatogram of compound **22** | 27 |
| Figure 36S | FTIR spectrum of compound **22** | 27 |
| Figure 37S | $^1$H-NMR ($D_2O$ exchg.) spectrum of compound **23** | 28 |
| Figure 38S | Mass spectrum of compound **23** | 28 |
| Figure 39S | HPLC chromatogram of compound **23** | 29 |
| Figure 40S | FTIR spectrum of compound **23** | 29 |
| Figure 41S | $^1$H-NMR ($D_2O$ exchg.) spectrum of compound **24** | 30 |
| Figure 42S | Mass spectrum of compound **24** | 30 |

| | | |
|---|---|---|
| Figure 43S | HPLC chromatogram of compound **24** | 31 |
| Figure 44S | FTIR spectrum of compound **24** | 31 |
| Figure 45S | $^1$H-NMR ($D_2O$ exchg.) spectrum of compound **25** | 32 |
| Figure 46S | Mass spectrum of compound **25** | 32 |
| Figure 47S | HPLC chromatogram of compound **25** | 33 |
| Figure 48S | FTIR spectrum of compound **25** | 33 |
| Figure 49S | $^1$H-NMR ($D_2O$ exchg.) spectrum of compound **26** | 34 |
| Figure 50S | Mass spectrum of compound **26** | 34 |
| Figure 51S | HPLC chromatogram of compound **26** | 35 |
| Figure 52S | FTIR spectrum of compound **26** | 35 |
| Figure 53S | $^1$H-NMR ($D_2O$ exchg.) spectrum of compound **27** | 36 |
| Figure 54S | Mass spectrum of compound **27** | 36 |
| Figure 55S | HPLC chromatogram of compound **27** | 37 |
| Figure 56S | FTIR spectrum of compound **27** | 37 |
| Figure 57S | $^1$H-NMR ($D_2O$ exchg.) spectrum of compound **28** | 38 |
| Figure 58S | Mass spectrum of compound **28** | 38 |
| Figure 59S | HPLC chromatogram of compound **28** | 39 |
| Figure 60S | FTIR spectrum of compound **28** | 39 |

**Table 1S.** Summary of the physicochemical and calculated molecular properties of the synthesized molecules

| Code | logS | logS @ pH 7.4 | logP | logD | 2C9 pKi | hERG pIC50 | BBB log ([brain] :[blood]) | MW | HBD | HBA | TPSA | Flexibility | Rotatable Bonds |
|---|---|---|---|---|---|---|---|---|---|---|---|---|---|
| **1** | 2.066 | 3.741 | 2.679 | -0.3857 | 4.817 | 3.674 | -0.7722 | 320.3 | 2 | 6 | 93.06 | 0.25 | 6 |
| **14** | 0.5091 | 0.5091 | 4.099 | 4.099 | 5.635 | 4.624 | -0.3468 | 423.5 | 2 | 6 | 84.86 | 0.2727 | 9 |
| **15** | 0.5091 | 0.5091 | 4.099 | 4.099 | 5.635 | 4.624 | -0.3468 | 423.5 | 2 | 6 | 84.86 | 0.2727 | 9 |
| **16** | 0.4051 | 0.4051 | 4.447 | 4.447 | 5.829 | 4.539 | -0.3546 | 443.9 | 2 | 6 | 84.86 | 0.2727 | 9 |
| **17** | 1.439 | 1.439 | 3.216 | 3.216 | 5.394 | 4.324 | -0.4136 | 399.4 | 2 | 7 | 98 | 0.2903 | 9 |
| **18** | 1.053 | 1.053 | 3.548 | 3.548 | 5.627 | 4.635 | -0.4211 | 439.5 | 2 | 7 | 94.09 | 0.2941 | 10 |
| **19** | 0.556 | 0.556 | 4.162 | 4.162 | 5.698 | 4.647 | -0.3585 | 423.5 | 2 | 6 | 84.86 | 0.2727 | 9 |
| **20** | 0.4051 | 0.4051 | 4.447 | 4.447 | 5.831 | 4.679 | -0.3626 | 443.9 | 2 | 6 | 84.86 | 0.2727 | 9 |
| **21** | 0.9787 | 0.9787 | 2.68 | 2.68 | 5.474 | 4.245 | -0.4472 | 410.5 | 2 | 7 | 97.75 | 0.2812 | 9 |
| **22** | 0.9787 | 0.9787 | 2.68 | 2.68 | 5.494 | 4.261 | -0.4472 | 410.5 | 2 | 7 | 97.75 | 0.2812 | 9 |
| **23** | 0.556 | 0.556 | 4.162 | 4.162 | 5.698 | 4.501 | -0.3504 | 423.5 | 2 | 6 | 84.86 | 0.2727 | 9 |
| **24** | 1.003 | 1.003 | 4.647 | 4.647 | 5.697 | 4.965 | -0.3445 | 451.6 | 2 | 6 | 84.86 | 0.3429 | 12 |
| **25** | 0.8509 | 0.8509 | 3.081 | 3.081 | 5.946 | 4.465 | -0.4748 | 453.5 | 2 | 8 | 103.3 | 0.25 | 9 |
| **26** | 3.678 | 2.429 | 1.999 | 1.316 | 4.805 | 4.724 | -0.4472 | 432.5 | 2 | 8 | 97.33 | 0.303 | 10 |
| **27** | 0.8339 | 0.8339 | 3.757 | 3.757 | 5.637 | 4.666 | -0.3753 | 425.5 | 2 | 7 | 94.09 | 0.2727 | 9 |
| **28** | 0.7507 | 0.7507 | 3.717 | 3.717 | 5.651 | 4.884 | -0.3766 | 425.5 | 2 | 7 | 94.09 | 0.2727 | 9 |

**logS:** logarithm of the intrinsic aqueous solubility, S in µM, for neutral compounds
**logS@pH7.4:** logarithm of the apparent solubility at pH 7.4, S in µM, for ionised compounds
**logP:** logarithm of the octanol/water partition coefficient for neutral compounds
**logD:** logarithm of the octanol/water partition coefficient for ionised compounds at a fixed pH of 7.4
**2C9 pKi:** pKi values for affinity with CYP2C9
**hERG pIC50:** pIC50 values for inhibition of hERG K+ channels expressed in mammalian cells
**BBB log([brain]:[blood]):** logarithm of the Brain/Blood ratio
**MW:** Molecular weight
**HBD:** No. of H-bond donors
**HBA:** No. of H-bond acceptors
**TPSA:** Topological polar surface area (Å2)
**Flexibility:** ratio of rotatable bonds to total bonds

## Experimental Section

### General.

All the chemicals were purchased from the producers vendors such as Acros Organics (Geel, Belgium), Alfa Aesar (Karlsruhe, Germany), Sigma Aldrich (St. Louis, USA) or Merck (Darmstadt, Germany) and used without further purification. Solvents were used directly, unless specified. All the reactions were carried out under anhydrous conditions under $N_2$ atmosphere and the progress of the reaction was monitored by thin- layer chromatography (TLC) using silica gel 60 $F_{254}$ (Merck Millipore, Billerica, MA, USA) coated aluminium plates. All the title compounds were purified by column chromatography packed with silica gel of #230-400. Melting points of the compounds were determined on Veego VMP DS (General Trading Co., India) and were uncorrected. FT-IR spectra were recorded on a Perkin Elmer RX1 instrument (Waltham, MA). The purity of all the compounds was determined to be > 95% by HPLC. HPLC analysis was performed on Agilent 1220 Infinity system (Santa Clara, CA, USA). An isocratic mobile phase system consisting of (A) Acetonitrile and (B) Water (70:30, v/v) was used with a $C_{18}$ Kromasil® column (15 cm × 4.6 mm, 5 μm particle size, 100 Å pore size) at a flow rate of 1mL/min. Deionised water was obtained from EMD Millipore Elix™ Essential 3 water purification system. $^1$H-NMR spectra were recorded on Bruker Advance 400 MHz (Billerica, MA) instrument in DMSO-$d_6$ ($D_2O$ exchange), tetramethyl silane (TMS) as internal standard and the chemical shifts were expressed in $\delta$ (ppm) values. Mass spectra (MS) were recorded on Shimadzu 8040 LC-MS/MS system (Kyoto, Japan) using electrospray ionization (ESI) mode. Enzyme inhibition and MTT assay readings were recorded on EPOCH 2 Biotek microplate reader (Winooski, VT, USA). β-Nicotinamide adenine dinucleotide (NAD), inosine-5′-monophosphate (IMP), DL-dithiothreitol (DTT), potassium chloride (KCl), 3-(4,5-dimethyl thiazol-2-yl)-2,5-diphenyl tetrazolium bromide (MTT), Tris-HCl were obtained from SRL (Mumbai, India), foetal bovine serum (FBS), phosphate-buffered saline

(PBS), Dulbecco's modified eagle's medium (DMEM) and trypsin-EDTA were obtained from CellClone (Delhi, India) and the antibiotics from Hi-Media Laboratories Ltd. (Mumbai, India).

*(R)(E)-6-(4-hydroxy-6-methoxy-7-methyl-3-oxo-1,3-dihydroisobenzofuran-5-yl)-4-methyl-N-(1-phenylethyl)hex-4-enamide* ***(15).*** It was synthesized using **1** (0.1 g, 0.31 mmol), (*R*)-(+)-α-methylbenzylamine **13b** (0.045 mL, 0.35 mmol), DMAP (0.044 g, 0.35 mmol) and EDCI.HCl (0.066 g, 0.34 mmol) as per Method A to yield **15** as off-white solid. Yield: 58%; TLC: $R_f$ = 0.72 (DCM:EtOAc, 8:2); purity (HPLC): 96.67%; mp: 128-130 °C; IR (KBr) $cm^{-1}$ 3443 (OH, str), 3284 (NH, str), 1745 (O-C=O, str), 1638 (NH-C=O, str), 1549 (C=C, str), 1134 (C-O, str); $^1$H-NMR (DMSO-$d_6$, $D_2O$ exchange, 400 MHz) δ 7.29 – 7.12 (m, 5H), 5.20 (s, 2H), 5.08 (t, 1H), 4.79 (q, *J* = 7.1 Hz, 1H), 3.63 (s, 3H), 3.24 (d, *J* = 6.8 Hz, 2H), 2.18 – 2.06 (m, 4H), 2.03 (s, 3H), 1.69 (s, 3H), 1.22 (d, *J* = 7.0 Hz, 3H); MS (ESI) *m/z*: 422 $[M-H]^-$.

*(E)-N-(2-chlorobenzyl)-6-(4-hydroxy-6-methoxy-7-methyl-3-oxo-1,3-dihydroisobenzofuran-5-yl)-4-methylhex-4-enamide* ***(16).*** It was synthesized using **1** (0.1 g, 0.31 mmol), 2-chlorobenzylamine **13c** (0.042 mL, 0.35 mmol), DMAP (0.044 g, 0.35 mmol) and EDCI.HCl (0.066 g, 0.34 mmol) as Method A to yield **16** as off-white solid. Yield: 70%; TLC: $R_f$ = 0.65 (DCM:EtOAc, 8:2); purity (HPLC): 98.69%; mp: 150-152 °C; IR (KBr) $cm^{-1}$ 3423 (OH, str), 3284 (NH, str), 1750 (O-C=O, str), 1636 (NH-C=O, str), 1536 (C=C, str), 1136 (C-O, str); $^1$H-NMR (DMSO-$d_6$, $D_2O$ exchange, 400 MHz) δ 7.37 (d, *J* = 3.5, 5.4 Hz, 1H), 7.27 – 7.17 (m, 3H), 5.20 (s, 2H), 5.08 – 5.12 (t, *J* = 7.2 Hz, 1H), 4.21 (s, 2H), 3.64 (s, 3H), 3.26 (d, *J* = 6.9 Hz, 2H), 2.19 (dd, *J* = 8.4, 15.1 Hz, 4H), 2.03 (s, 3H), 1.71 (s, 3H); MS (ESI) *m/z*: 442 $[M-H]^-$.

*(E)-N-(furan-2-ylmethyl)-6-(4-hydroxy-6-methoxy-7-methyl-3-oxo-1,3-dihydroisobenzofuran-5-yl)-4-methylhex-4-enamide* ***(17).*** It was synthesized using **1** (0.1 g, 0.31 mmol),

furfurylamine **13d** (0.031 mL, 0.35 mmol), DMAP (0.044 g, 0.35 mmol) and EDCI.HCl (0.066 g, 0.34 mmol) as per Method A to yield **17** as off-white solid. Yield: 59%; TLC: $R_f$ = 0.59 (DCM:EtOAc, 8:2); purity (HPLC): 99.99%; mp: 118-120 °C; IR (KBr) $cm^{-1}$ 3426 (OH, str), 3289 (NH, str), 1745 (O-C=O, str), 1641 (NH-C=O, str), 1548 (C=C, str), 1137 (C-O, str); $^1$H-NMR (DMSO-$d_6$, $D_2O$ exchange, 400 MHz) δ 7.47 (d, J = 1.7 Hz, 1H), 6.32 (t, J = 1.8, 3.2 Hz, 1H), 6.14 (d, J = 3.1 Hz, 1H), 5.10 – 5.07 (m, 3H), 4.14 (s, 2H), 3.59 (s, 3H), 3.19 (d, J = 6.8 Hz, 2H), 2.14 – 2.08 (m, 4H), 1.96 (s, 3H), 1.67 (s, 3H); MS (ESI) *m/z*: 398 $[M-H]^-$.

*(E)-6-(4-hydroxy-6-methoxy-7-methyl-3-oxo-1,3-dihydroisobenzofuran-5-yl)-N-(4-methoxy-benzyl)-4-methylhex-4-enamide* ***(18).*** It was synthesized using **1** (0.1 g, 0.31 mmol), 4-methoxybenzylamine **13e** (0.046 mL, 0.35 mmol), DMAP (0.044 g, 0.35 mmol) and EDCI.HCl (0.066 g, 0.34 mmol) as per Method A to yield **18** as off-white solid. Yield: 54%; TLC: $R_f$ = 0.63 (DCM:EtOAc, 8:2); purity (HPLC): 99.99%; mp: 144-146 °C; IR (KBr) $cm^{-1}$ 3463 (OH, str), 3302 (NH, str), 1738 (O-C=O, str), 1641 (NH-C=O, str), 1550 (C=C, str), 1136 (C-O, str); $^1$H-NMR (DMSO-$d_6$, $D_2O$ exchange, 400 MHz) δ 7.08 (d, *J* = 8.3 Hz, 2H), 6.80 (d, *J* = 8.4 Hz, 2H), 5.20 (s, 2H), 5.10 – 5.07 (t, *J* = 7.1 Hz, 1H), 4.09 (s, 2H), 3.67 (s, 3H), 3.64 (s, 3H), 3.26 (d, *J* = 6.8 Hz, 2H), 2.14 (s, 4H), 2.04 (s, 3H), 1.70 (s, 3H); MS (ESI) *m/z*: 438 $[M-H]^-$.

*(E)-6-(4-hydroxy-6-methoxy-7-methyl-3-oxo-1,3-dihydroisobenzofuran-5-yl)-4-methyl-N-(4-methylbenzyl)hex-4-enamide* ***(19).*** It was synthesized using **1** (0.1 g, 0.31 mmol), 4-methybenzylamine **13f** (0.045 mL, 0.35 mmol), DMAP (0.044 g, 0.35 mmol) and EDCI.HCl (0.066 g, 0.34 mmol) as per Method A to yield **19** as off-white solid. Yield: 58%; TLC: $R_f$ = 0.67 (DCM:EtOAc, 8:2); purity (HPLC): 99.67%; mp: 154-156 °C; IR (KBr) $cm^{-1}$ 3444 (OH, str), 3268 (NH, str), 1750 (O-C=O, str), 1645 (NH-C=O, str), 1556 (C=C, str), 1132 (C-O, str); $^1$H-NMR (DMSO-$d_6$, $D_2O$ exchange, 400 MHz) δ 7.20 (d, *J* = 7.7 Hz, 2H), 7.09 (d, *J* = 7.7 Hz,

2H), 5.14 – 5.10 (m, 3H), 4.11 (s, 2H), 3.61(s, 3H), 3.23 (d, *J* = 6.8 Hz, 2H), 2.22 (d, *J* = 9.4 Hz, 4H), 2.14 (s, 3H), 2.01 (s, 3H), 1.69 (s, 3H); MS (ESI) *m/z*: 422 $[M-H]^-$.

*(E)-N-(4-chlorobenzyl)-6-(4-hydroxy-6-methoxy-7-methyl-3-oxo-1,3-dihydroisobenzofuran-5-yl)-4-methylhex-4-enamide* ***(20).*** It was synthesized using **1** (0.1 g, 0.31 mmol), 4-chlorobenzylamine **13g** (0.043 mL, 0.35 mmol), DMAP (0.044 g, 0.35 mmol) and EDCI.HCl (0.066 g, 0.34 mmol) as per Method A to yield **20** as off-white solid. Yield: 75%; TLC: $R_f$ = 0.64 (DCM:EtOAc, 8:2); purity (HPLC): 99.99%; mp: 142-144 °C; IR (KBr) $cm^{-1}$ 3422 (OH, str), 3287 (NH, str), 1729 (O-C=O, str), 1641 (NH-C=O, str), 1549 (C=C, str), 1141 (C-O, str); $^1$H-NMR (DMSO-$d_6$, $D_2O$ exchange, 400 MHz) δ 7.27 (dd, *J* = 1.8, 8.3 Hz, 2H), 7.16 (dd, 2H), 5.20 (s, 2H), 5.10 – 5.07 (t, *J* = 7.1 Hz, 1H), 4.14 (s, 2H), 3.63 (d, *J* = 1.7 Hz, 3H), 3.25 (d, *J* = 6.8 Hz, 2H), 2.18 – 2.15 (m, 4H), 2.04 (s, 3H), 1.70 (s, 3H); MS (ESI) *m/z*: 442 $[M-H]^-$.

*(E)-6-(4-hydroxy-6-methoxy-7-methyl-3-oxo-1,3-dihydroisobenzofuran-5-yl)-4-methyl-N-(pyridin-4-ylmethyl)hex-4-enamide* ***(21).*** It was synthesized using **1** (0.1 g, 0.31 mmol), 4-(aminomethyl)pyridine **13h** (0.036 mL, 0.35 mmol), DMAP (0.044 g, 0.35 mmol) and EDCI.HCl (0.066 g, 0.34 mmol) as per Method A to yield **21** as light-brown solid. Yield: 45%; TLC: $R_f$ = 0.43 (DCM:EtOAc, 8:2); purity (HPLC): 99.21%; mp: 120-122 °C; IR (KBr) $cm^{-1}$ 3303 (NH, str), 1767 (O-C=O, str), 1643 (NH-C=O, str), 1560 (C=C, str), 1132 (C-O, str); $^1$H-NMR (DMSO-$d_6$, $D_2O$ exchange, 400 MHz) δ 8.40 (d, *J* = 5.0 Hz, 2H), 7.17 (d, *J* = 5.0 Hz, 2H), 5.20 (s, 2H), 5.11 (t, 1H), 4.20 (s, 2H), 3.64 (s, 3H), 3.26 (d, *J* = 6.9 Hz, 2H), 2.26 – 2.11 (m, 4H), 2.03 (s, 3H), 1.71 (s, 3H); MS (ESI) *m/z*: 409 $[M-H]^-$.

*(E)-6-(4-hydroxy-6-methoxy-7-methyl-3-oxo-1,3-dihydroisobenzofuran-5-yl)-4-methyl-N-(pyridin-2-ylmethyl)hex-4-enamide* ***(22).*** It was synthesized using **1** (0.1 g, 0.31 mmol), 2-(aminomethyl)pyridine **13i** (0.036 mL, 0.35 mmol), DMAP (0.044 g, 0.35 mmol) and EDCI.HCl (0.066 g, 0.34 mmol) as per Method A to yield **22** as light-brown solid. Yield: 40%;

TLC $R_f$ 0.41 (DCM:EtOAc, 8:2); purity (HPLC): 99.17%; mp: 158-160 °C; IR (KBr) $cm^{-1}$ 3416 (OH, str), 3284 (NH, str), 1761 (O-C=O, str), 1688 (NH-C=O, str), 1563 (C=C, str), 1149 (C-O, str); $^1$H-NMR (DMSO-$d_6$, $D_2O$ exchange, 400 MHz) δ 8.41 (s, 1H), 7.69 (t, *J* = 8.3 Hz, 1H), 7.20 (dd, *J* = 7.4, 8.3 Hz, 2H), 5.12 (s, 3H), 4.25 (s, 2H), 3.58 (s, 3H), 3.18 (s, 2H), 2.26 – 2.03 (m, 4H), 1.95 (s, 3H), 1.67 (s, 3H); MS (ESI) *m/z*: 409 $[M-H]^-$.

*(E)-6-(4-hydroxy-6-methoxy-7-methyl-3-oxo-1,3-dihydroisobenzofuran-5-yl)-4-methyl-N-(2-methylbenzyl)hex-4-enamide* ***(23).*** It was synthesized using **1** (0.1 g, 0.31 mmol), 2-methylbenzylamine **13j** (0.043 mL, 0.35 mmol), DMAP (0.044 g, 0.35 mmol) and EDCI.HCl (0.066 g, 0.34 mmol) as per Method A to yield **23** as off-white solid. Yield: 54%; TLC: $R_f$ = 0.68 (DCM:EtOAc, 8:2); purity (HPLC): 99%; mp: 130-132 °C; IR (KBr) $cm^{-1}$ 3433 (OH, str), 3291 (NH, str), 1742 (O-C=O, str), 1639 (NH-C=O, str), 1548 (C=C, str), 1135 (C-O, str); $^1$H-NMR (DMSO-$d_6$, $D_2O$ exchange, 400 MHz) δ 7.13 – 7.04 (m, 4H), 5.20 (s, 2H), 5.09 (t, 1H), 4.12 (s, 2H), 3.64 (s, 3H), 3.25 (d, *J* = 6.8 Hz, 2H), 2.23 – 2.11 (m, 7H), 2.03 (s, 3H), 1.70 (s, 3H); MS (ESI) *m/z*: 422 $[M-H]^-$.

*(E)-6-(4-hydroxy-6-methoxy-7-methyl-3-oxo-1,3-dihydroisobenzofuran-5-yl)-4-methyl-N-(4-phenylbutyl)hex-4-enamide* ***(24).*** It was synthesized using **1** (0.1 g, 0.31 mmol), 4-phenylbutylamine **13k** (0.055 mL, 0.35 mmol), DMAP (0.044 g, 0.35 mmol) and EDCI.HCl (0.066 g, 0.34 mmol) as per Method A to yield **24** as off-white solid. Yield: 60%; TLC: $R_f$ = 0.71 (DCM:EtOAc, 8:2); purity (HPLC): 99.83%; mp: 140-142 °C; IR (KBr) $cm^{-1}$ 3449 (OH, str), 3310 (NH, str), 1745 (O-C=O, str), 1639 (NH-C=O, str), 1561 (C=C, str), 1134 (C-O, str); $^1$H-NMR (DMSO-$d_6$, $D_2O$ exchange, 400 MHz) δ 7.31 – 7.03 (m, 5H), 5.18 (s, 2H), 5.06 (t, 1H), 3.80 (s, 3H), 3.23 (d, *J* = 6.9 Hz, 2H), 2.94 (t, *J* = 7.0 Hz, 2H), 2.47 (t, 2H), 2.08 (s, 4H), 2.03 (s, 3H), 1.68 (s, 3H), 1.45 (p, *J* = 7.7 Hz, 2H), 1.28 (p, *J* = 7.1 Hz, 2H); MS (ESI) *m/z*: 450 $[M-H]^-$.

*(E)-N-(benzo[d][1,3]dioxol-5-ylmethyl)-6-(4-hydroxy-6-methoxy-7-methyl-3-oxo-1,3-dihydr-isobenzofuran-5-yl)-4-methylhex-4-enamide* ***(25).*** It was synthesized using **1** (0.1 g, 0.31 mmol), 3,4-(methylenedioxy)benzylamine **13l** (0.044 mL, 0.35 mmol), DMAP (0.044 g, 0.35 mmol) and EDCI.HCl (0.066 g, 0.34 mmol) as per Method A to yield **25** as off-white solid. Yield: 56%; TLC: $R_f$ = 0.62 (DCM:EtOAc, 8:2); purity (HPLC): 99.31%; mp: 100-102 °C; IR (KBr) $cm^{-1}$ 3425 (OH, str), 3283 (NH, str), 1739 (O-C=O, str), 1640 (NH-C=O, str), 1551 (C=C, str), 1135 (C-O, str); $^1$H-NMR (DMSO-$d_6$, $D_2O$ exchange, 400 MHz) δ 6.76 (d, *J* = 1.9, 8.0 Hz, 1H), 6.71 (s, 1H), 6.64 (d, *J* = 8.0 Hz, 1H), 5.91 (s, 2H), 5.20 (s, 2H), 5.07 – 5.10 (t, *J* = 6.9 Hz, 1H), 4.07 (s, 2H), 3.65 (s, 3H), 3.26 (d, *J* = 6.8 Hz, 2H), 2.14 (s, 4H), 2.04 (d, *J* = 1.8 Hz, 3H), 1.70 (s, 3H); MS (ESI) *m/z*: 452 $[M-H]^-$.

*(E)-6-(4-hydroxy-6-methoxy-7-methyl-3-oxo-1,3-dihydroisobenzofuran-5-yl)-4-methyl-N-(2-morpholinoethyl)hex-4-enamide* ***(26).*** It was synthesized using **1** (0.1 g, 0.31 mmol), 4-(2-aminoethyl)morpholine **13m** (0.046 mL, 0.35 mmol), DMAP (0.044 g, 0.35 mmol) and EDCI.HCl (0.066 g, 0.34 mmol) as per Method A to yield **26** as off-white solid. Yield: 55%; TLC: $R_f$ = 0.57 (DCM:EtOAc, 8:2); purity (HPLC): 99.99%; mp: 124-126 °C; IR (KBr) $cm^{-1}$ 3428 (OH, str), 3292 (NH, str), 1739 (O-C=O, str), 1642 (NH-C=O, str), 1562 (C=C, str), 1117 (C-O, str); $^1$H-NMR (DMSO-$d_6$, $D_2O$ exchange, 400 MHz) δ 5.20 (s, 2H), 5.04 – 5.08 (t, *J* = 7.0 Hz, 1H), 3.65 (s, 3H), 3.50 (t, *J* = 4.6 Hz, 4H), 3.25 (d, *J* = 6.8 Hz, 2H), 3.05 (t, *J* = 6.9 Hz, 2H), 2.31 – 2.23 (m, 4H), 2.20 (t, *J* = 7.0 Hz, 2H), 2.09 (s, 4H), 2.04 (s, 3H), 1.69 (s, 3H); MS (ESI) *m/z*: 431 $[M-H]^-$.

*(E)-6-(4-hydroxy-6-methoxy-7-methyl-3-oxo-1,3-dihydroisobenzofuran-5-yl)-N-(4-methoxy phenyl)-4-methylhex-4-enamide* ***(28).*** It was synthesized using **1** (0.1 g, 0.31 mmol), 4-methoxyaniline **13o** (0.026 g, 0.21 mmol), HATU (0.158 g, 0.41 mmol) and DIPEA (0.185 mL, 1.06 mmol) as per Method B to yield **28** as off-white solid. Yield: 48%; TLC: $R_f$ = 0.60

(DCM:EtOAc, 8:2); purity (HPLC): 99.99%; mp: 166-168 °C; IR (KBr) $cm^{-1}$ 3302 (OH, str), 1753 (O-C=O, str), 1657 (NH-C=O, str), 1613 (C=C, str), 1132 (C-O, str); $^1$H-NMR (DMSO-$d_6$, $D_2O$ exchange, 400 MHz) δ 7.31 (d, $J$ = 8.5 Hz, 2H), 6.77 (d, $J$ = 8.5 Hz, 2H), 5.17 (s, 2H), 5.14 – 5.12 (t, $J$ = 7.0 Hz, 1H), 3.66 (s, 3H), 3.60 (s, 3H), 3.25 (d, $J$ = 6.8 Hz, 2H), 2.28 (t, $J$ = 7.4 Hz, 2H), 2.19 (t, $J$ = 7.5 Hz, 2H), 2.00 (s, 3H), 1.73 (s, 3H); MS (ESI) *m/z*: 424 $[M-H]^-$.

**Protocol for human peripheral blood mononuclear cells (hPBMC) assay**

hPBMCs were separated from heparinized blood by density gradient centrifugation in Ficoll-Urotropine. Cells were placed in 96-well round-bottom plates (0.1 million cells/well) and Dynabeads® human T-activator CD3/CD28 for cell expansion and activation (culture medium - RPMI containing 10% heat inactivated FBS and 100 units/ml of penicillin and streptomycin). The compound were dissolved in DMSO, the final concentration of which was 1% and 1% DMSO treated cells considered as a control. Compound **24** and MPA**(1)** were tested at 10 μM concentration in triplicate and incubated with the compounds for 48 h (37°C, atmosphere 5% $CO_2$). MTT solution (20 μL; 5 mg/mL) was added to each well and incubated for 4 h. After this time, the supernatant was removed and 0.4N HCl/ isopropanol was added in order to dissolve the formazan crystals. The absorbance was measured at 570 nm (Perkin Elmer, Victor X4).

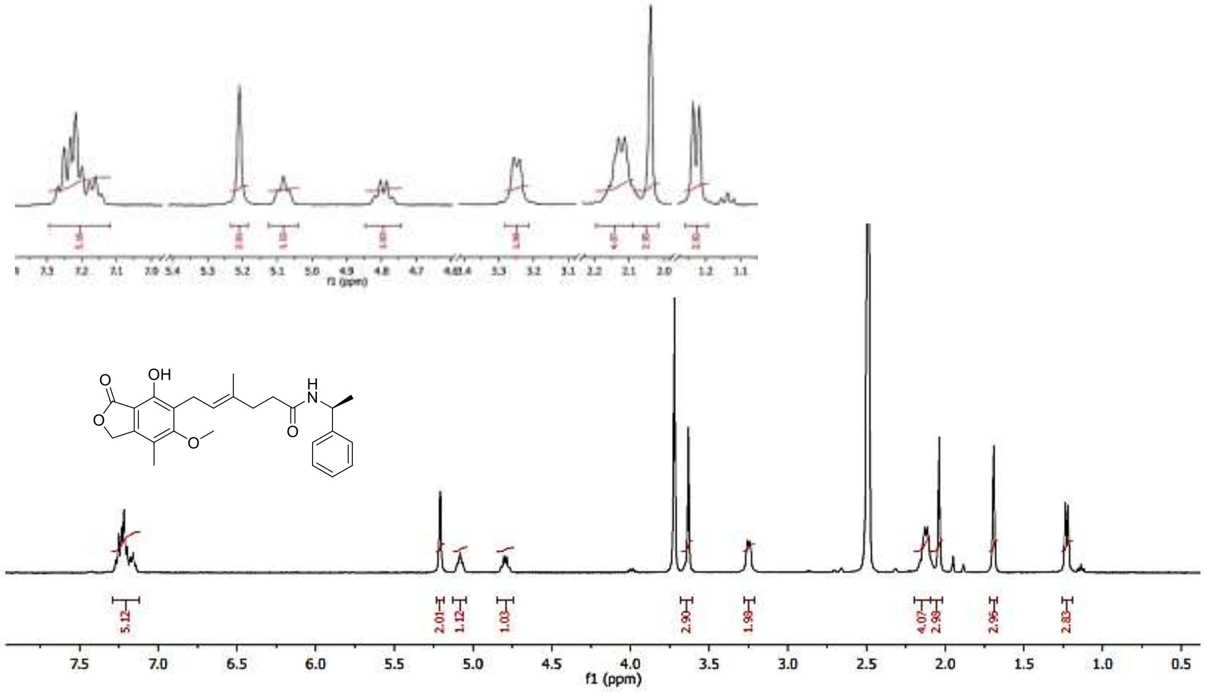

**Figure 1S.** $^{1}$H-NMR spectrum ($D_2O$ exchg.) of compound **14**

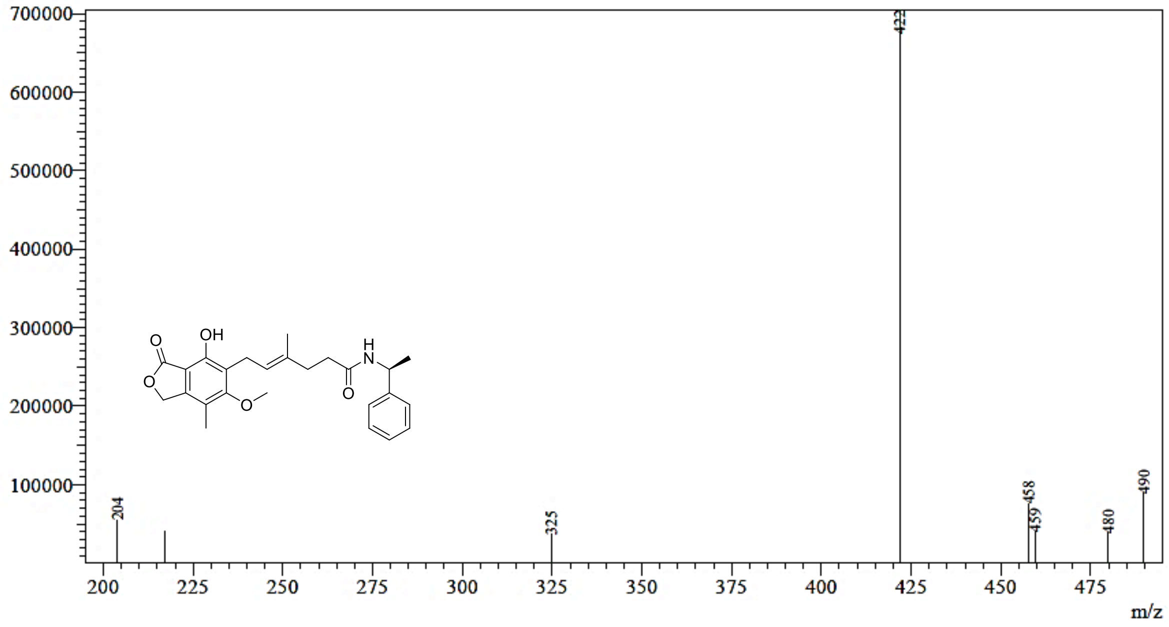

**Figure 2S.** Mass spectrum of compound **14**

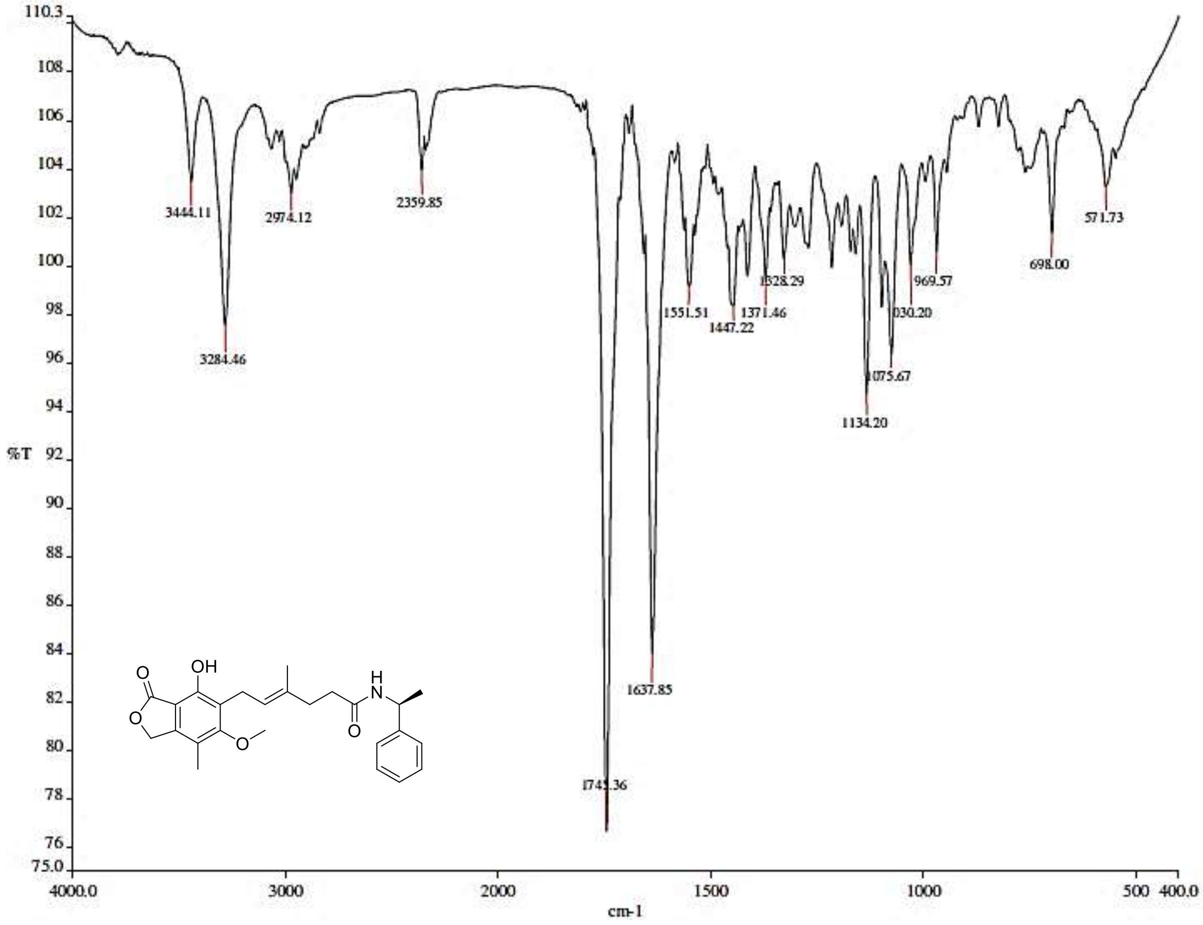


**Figure 3S.** FT-IR spectrum of compound **14**

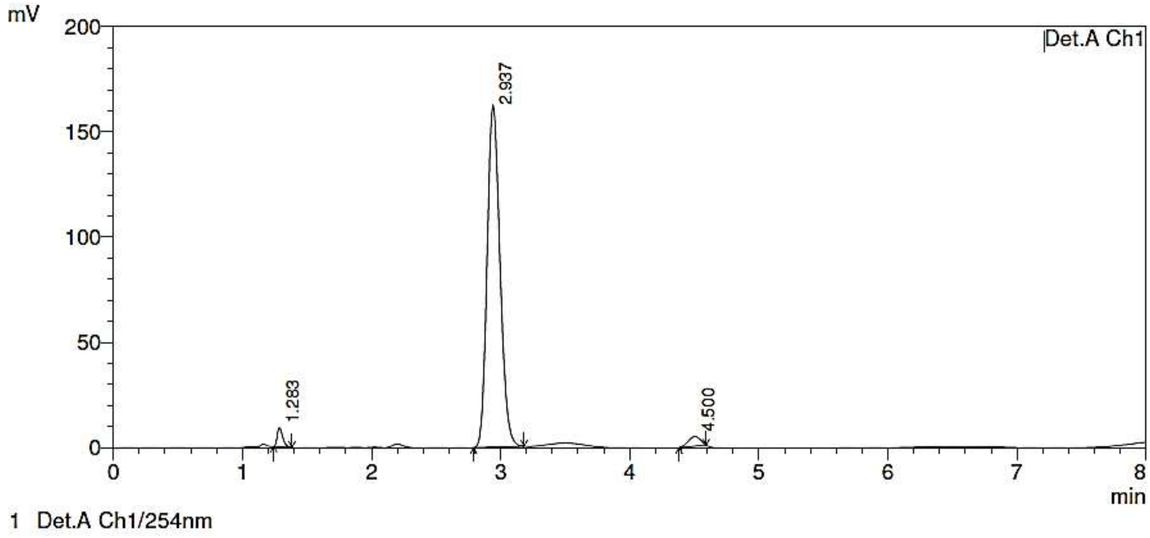


PeakTable

Detector A Ch1 254nm

| Peak# | Ret. Time | Area | Height | Area % | Height % |
|---|---|---|---|---|---|
| 1 | 1.283 | 26474 | 8996 | 2.251 | 5.111 |
| 2 | 2.937 | 1120522 | 162487 | 95.284 | 92.328 |
| 3 | 4.500 | 28990 | 4507 | 2.465 | 2.561 |
| Total | | 1175986 | 175989 | 100.000 | 100.000 |

**Figure 4S.** HPLC chromatogram of compound **14**

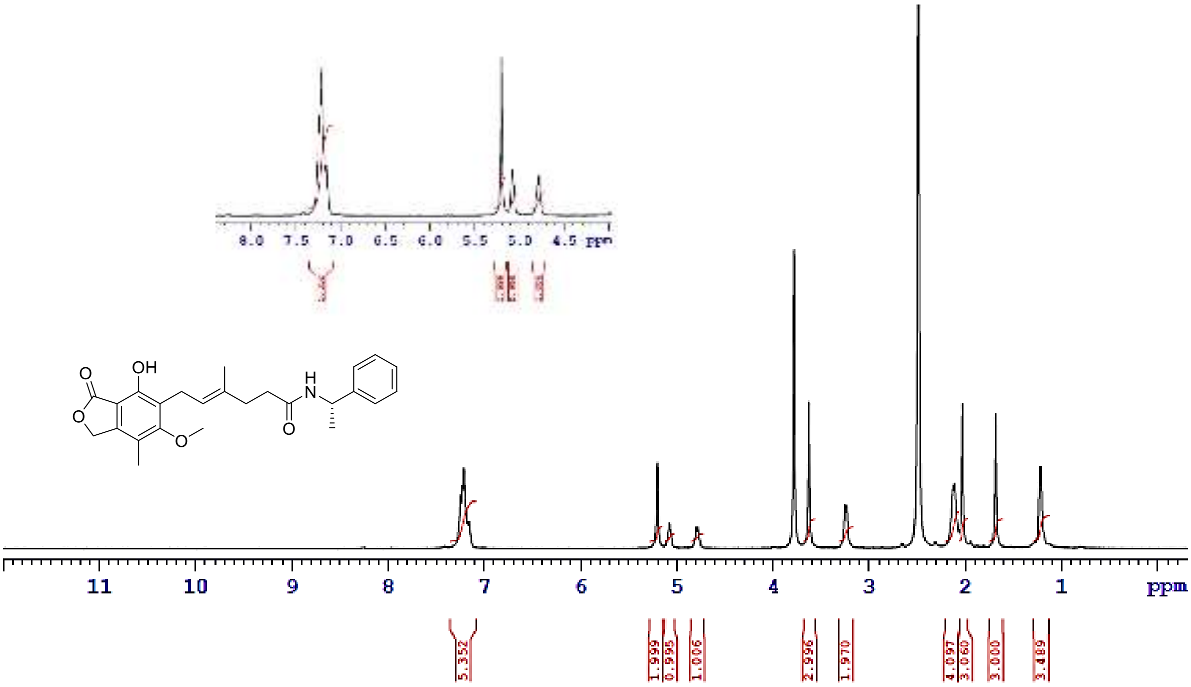


**Figure 5S.** $^{1}$H-NMR spectrum ($D_2O$ exchg.) of compound **15**

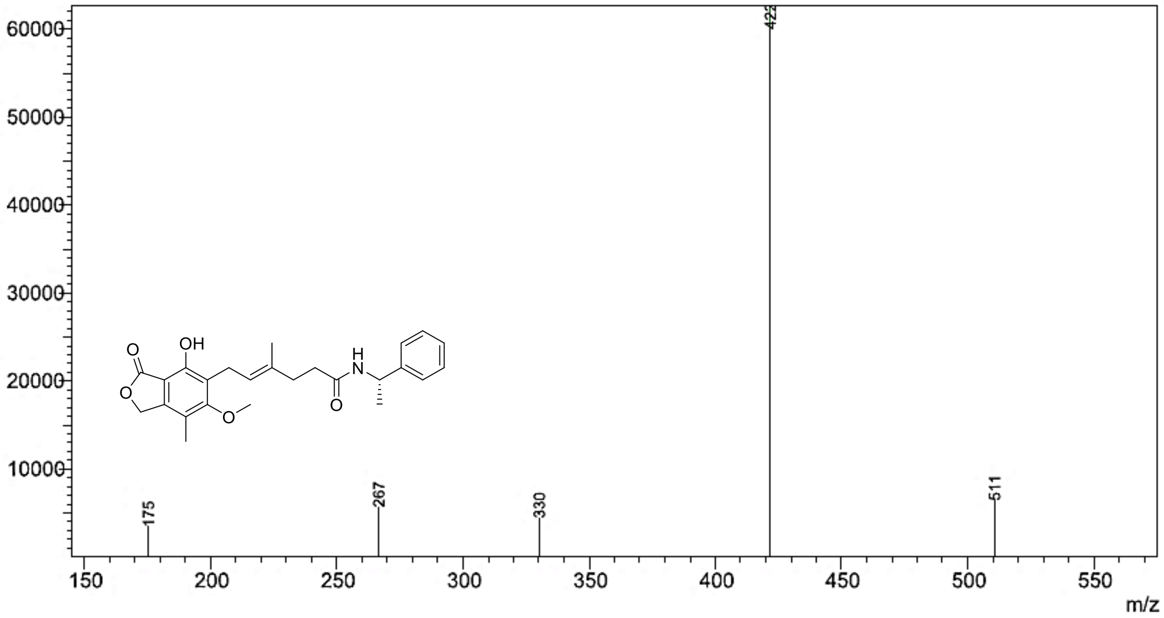


**Figure 6S.** Mass spectrum of compound **15**

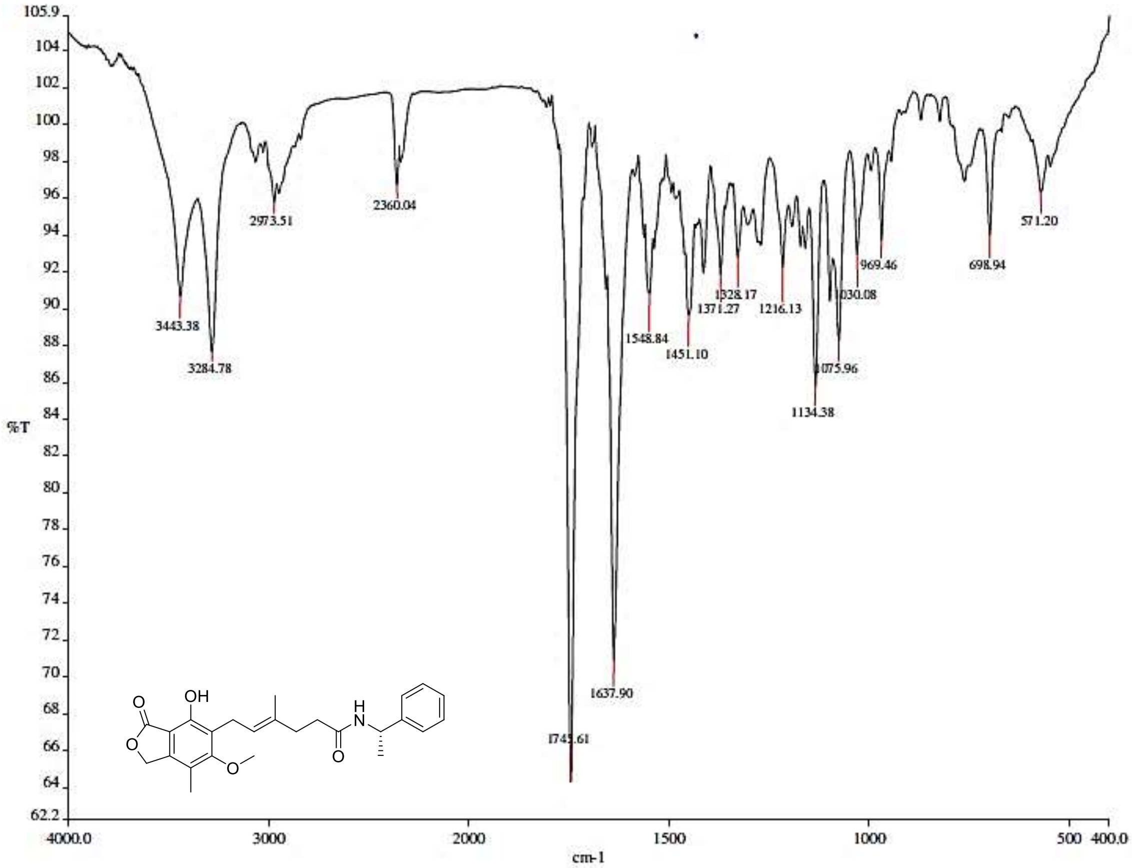


**Figure 7S.** FT-IR spectrum of compound **15**

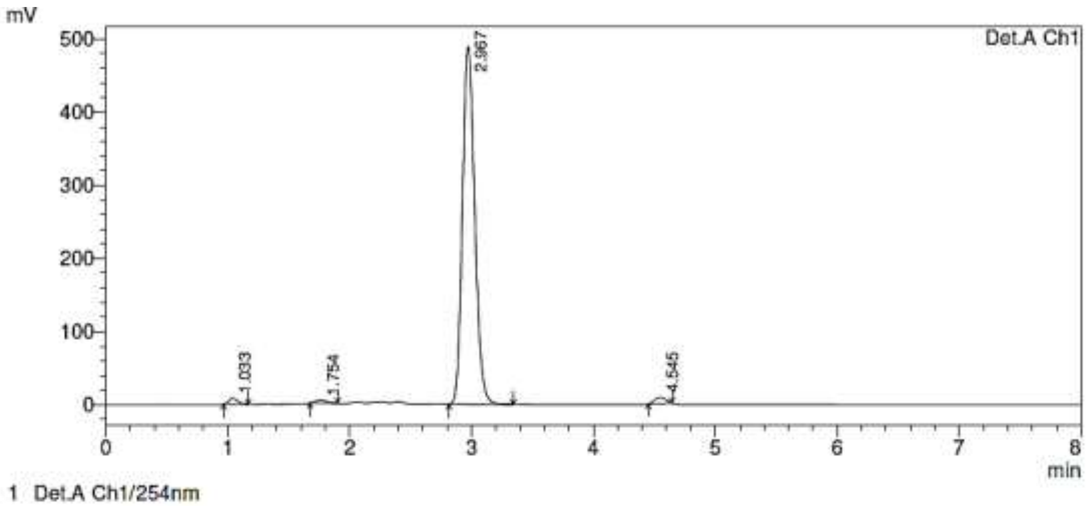


PeakTable

Detector A Ch1 254nm

| Peak# | Ret. Time | Area | Height | Area % | Height % |
|---|---|---|---|---|---|
| 1 | 1.033 | 41631 | 7940 | 1.174 | 1.559 |
| 2 | 1.754 | 25898 | 4694 | 0.731 | 0.922 |
| 3 | 2.967 | 3427192 | 488553 | 96.672 | 95.939 |
| 4 | 4.545 | 50466 | 8044 | 1.423 | 1.580 |
| Total | | 3545187 | 509231 | 100.000 | 100.000 |

**Figure 8S.** HPLC chromatogram of compound **15**

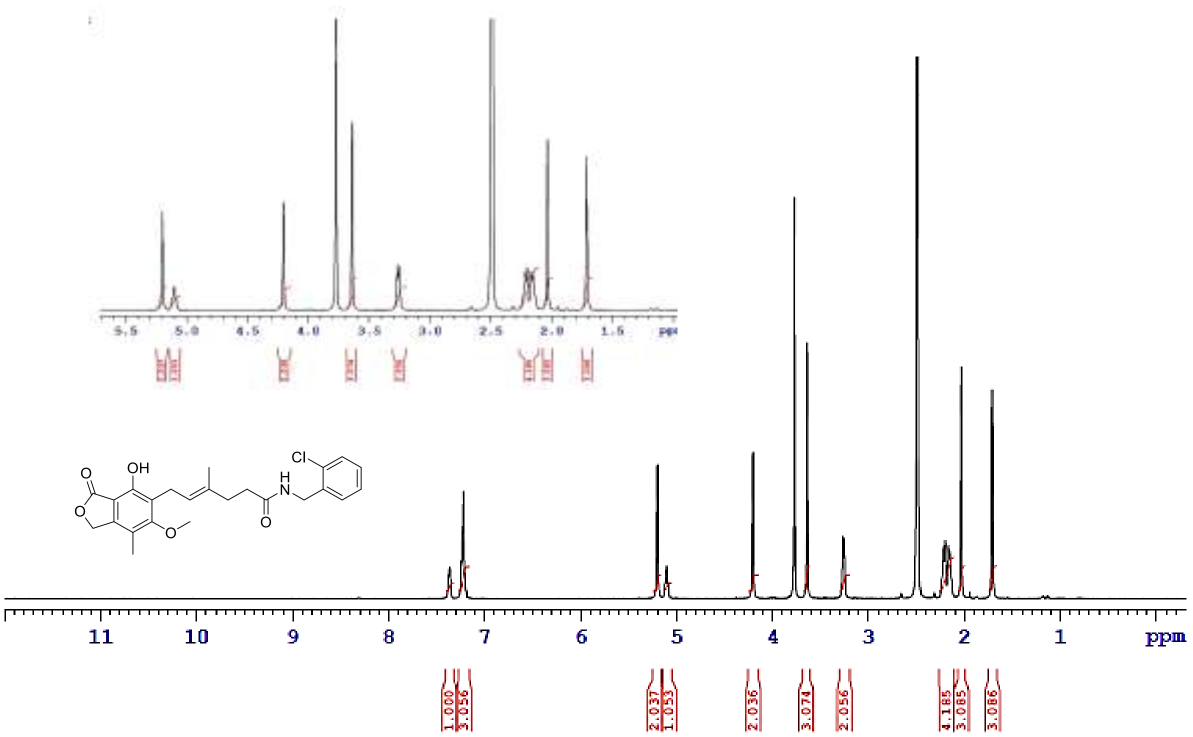


**Figure 9S.** $^{1}$H-NMR spectrum ($D_2O$ exchg.) of compound **16**

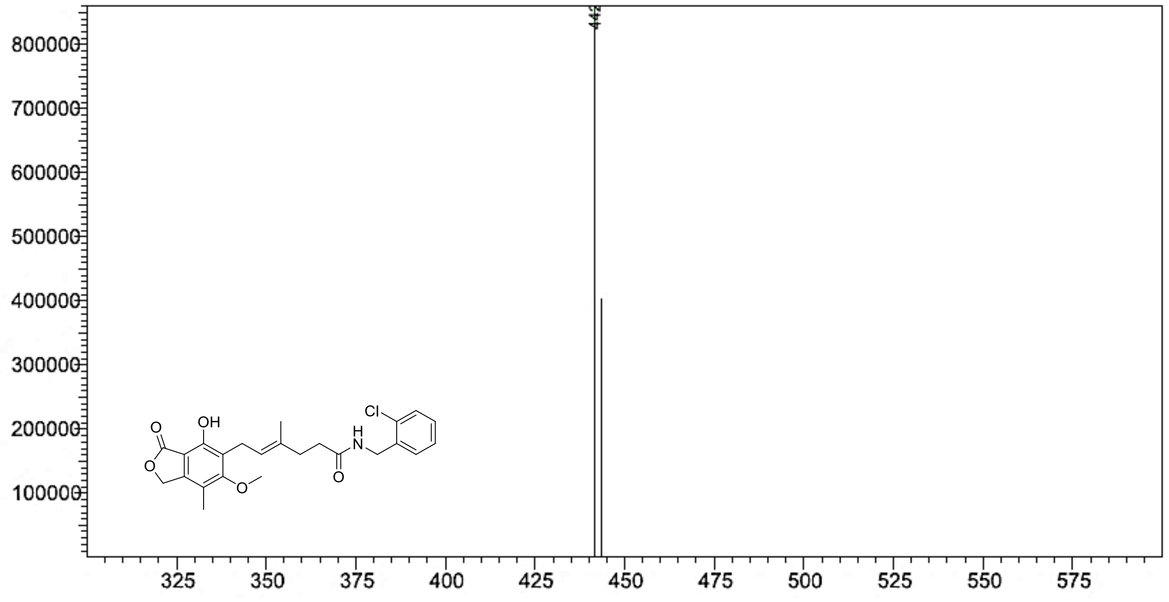


**Figure 10S.** Mass spectrum of compound **16**

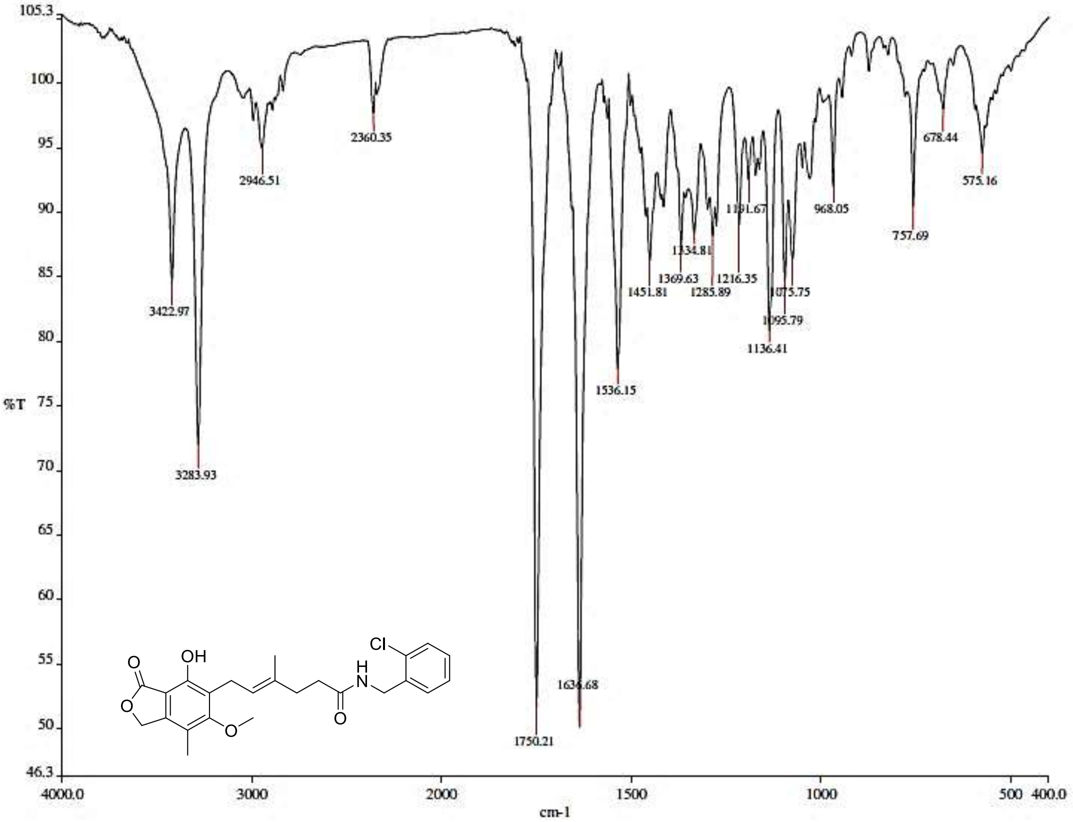


**Figure 11S.** FT-IR spectrum of compound **16**

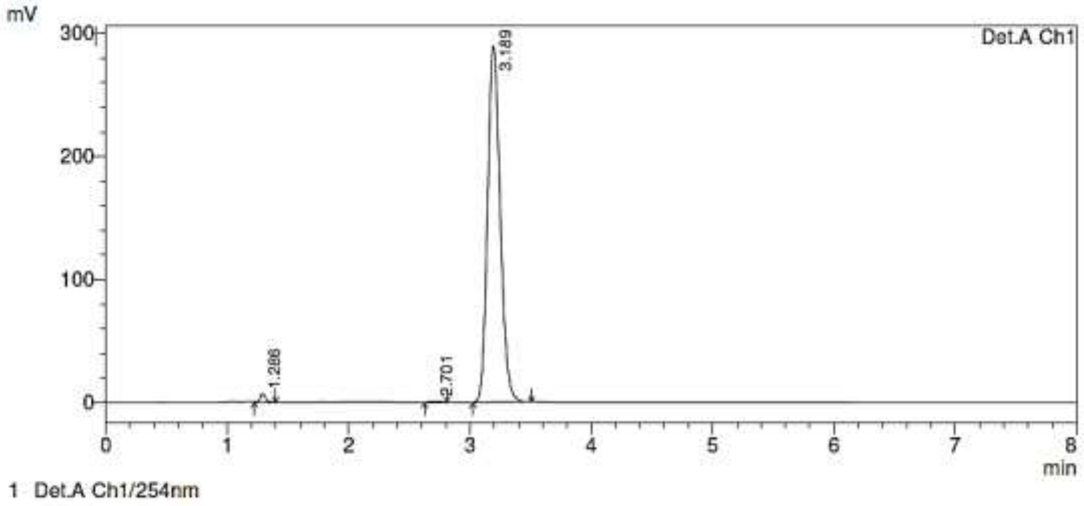


PeakTable

Detector A Ch1 254nm

| Peak# | Ret. Time | Area | Height | Area % | Height % |
|---|---|---|---|---|---|
| 1 | 1.286 | 21232 | 7096 | 1.000 | 2.382 |
| 2 | 2.701 | 6544 | 1150 | 0.308 | 0.386 |
| 3 | 3.189 | 2094609 | 289709 | 98.691 | 97.232 |
| Total | | 2122384 | 297956 | 100.000 | 100.000 |

**Figure 12S.** HPLC chromatogram of compound **16**

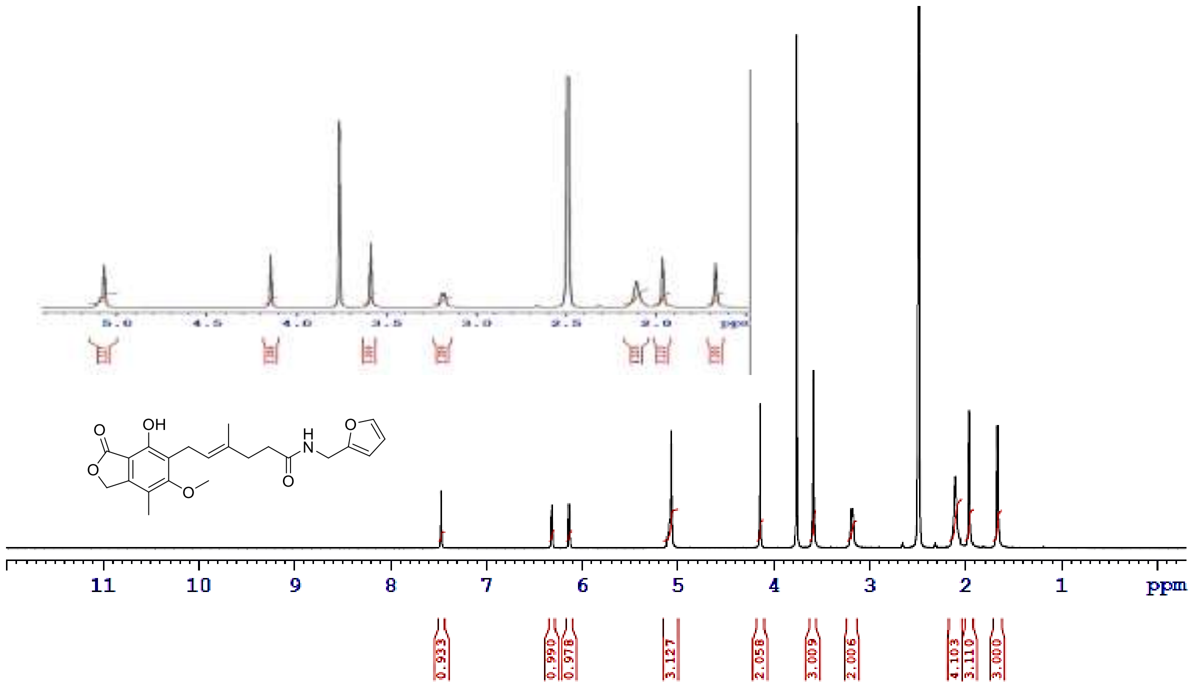


**Figure 13S.** $^{1}$H-NMR spectrum ($D_2O$ exchg.) of compound **17**

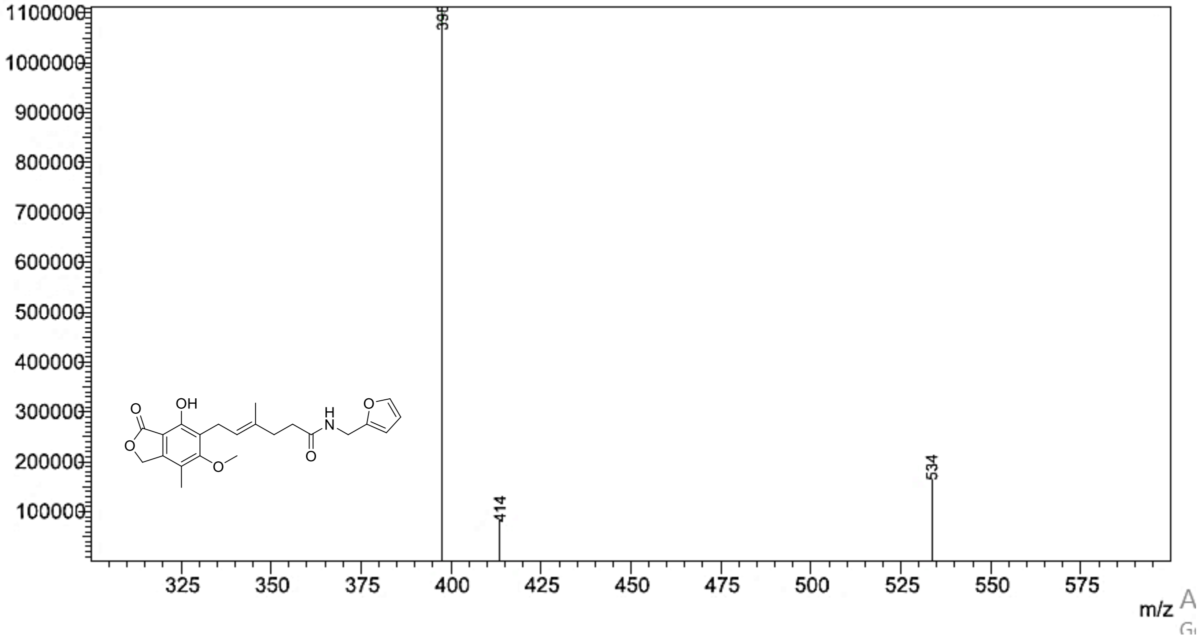


**Figure 14S.** Mass spectrum of compound **17**

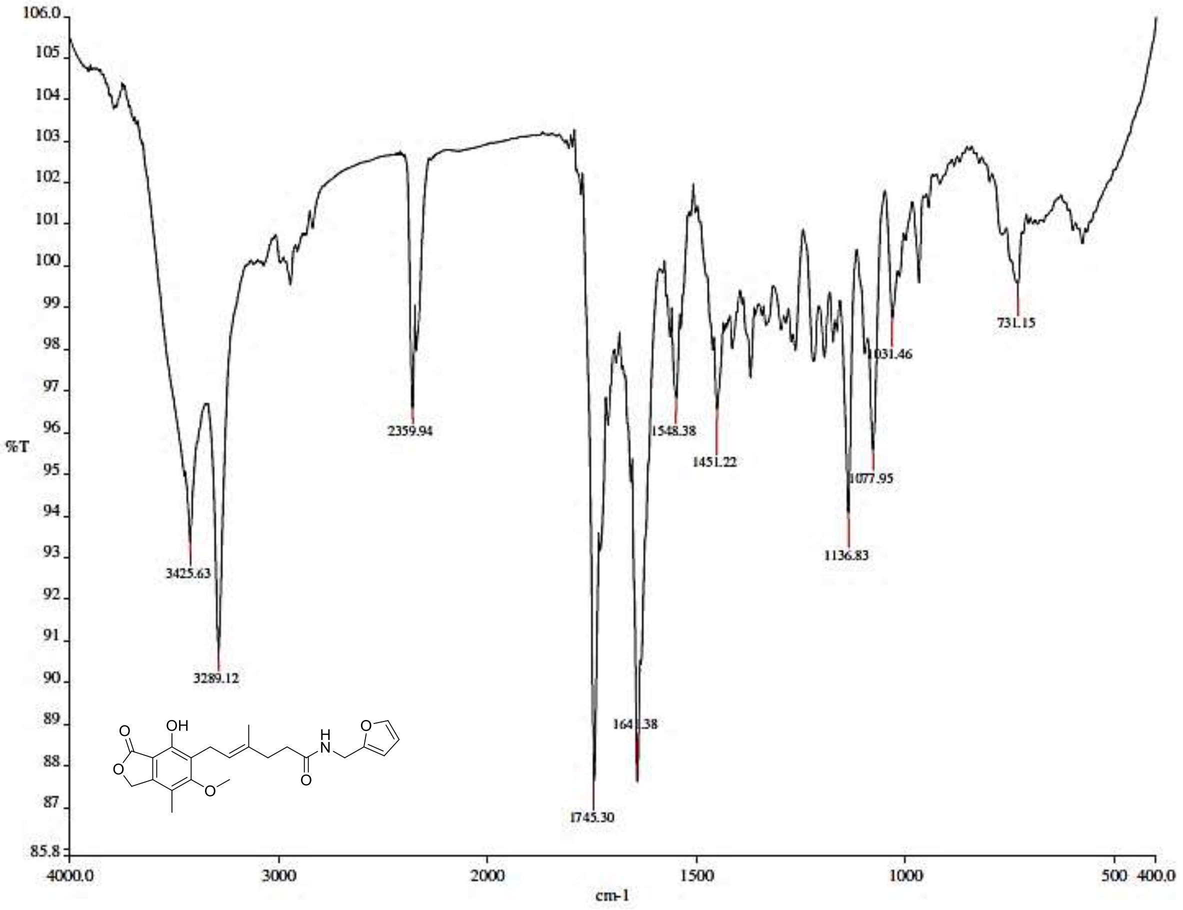


**Figure 15S.** FT-IR spectrum of compound **17**

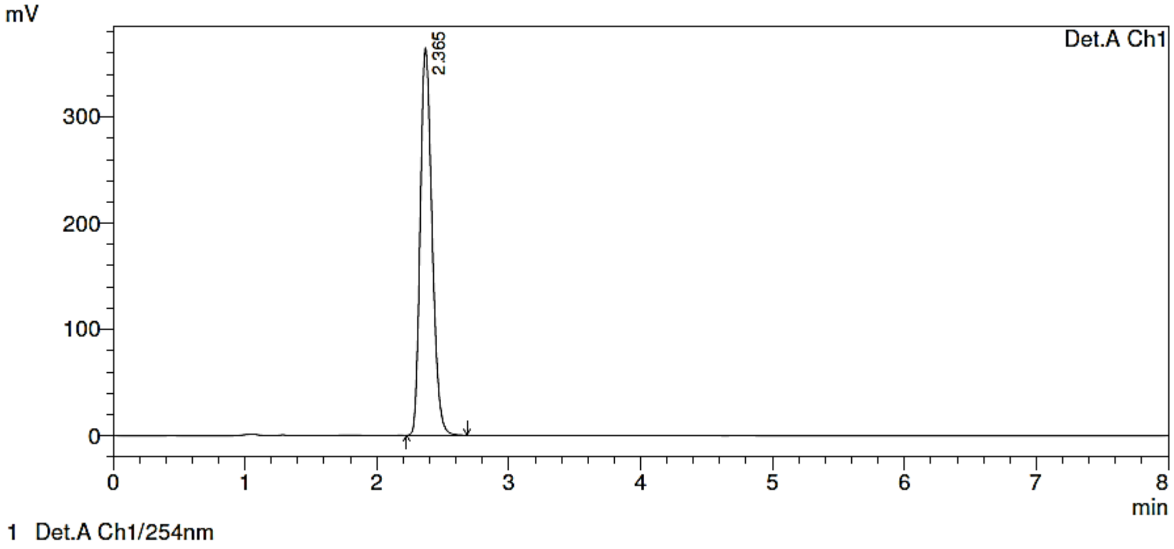


PeakTable

Detector A Ch1 254nm

| Peak# | Ret. Time | Area | Height | Area % | Height % |
|---|---|---|---|---|---|
| 1 | 2.365 | 2264238 | 364544 | 100.000 | 100.000 |
| Total | | 2264238 | 364544 | 100.000 | 100.000 |

Act

**Figure 16S.** HPLC chromatogram of compound **17**

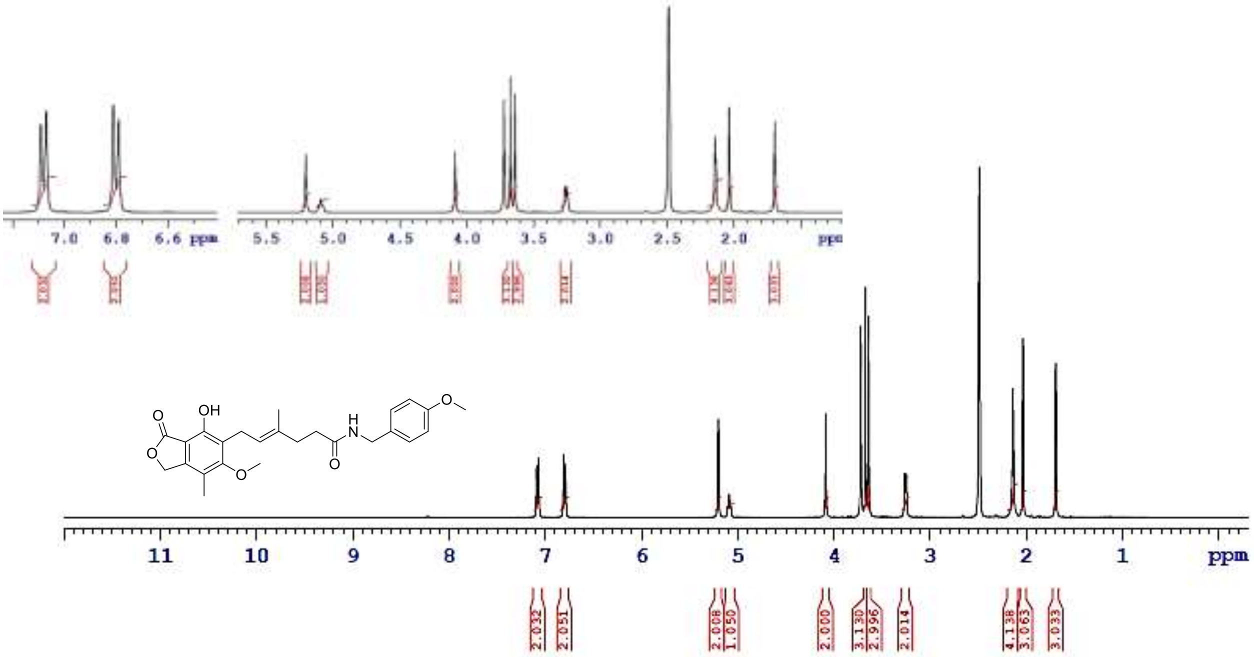

**Figure 17S.** $^{1}$H-NMR spectrum ($D_2O$ exchg.) of compound **18**

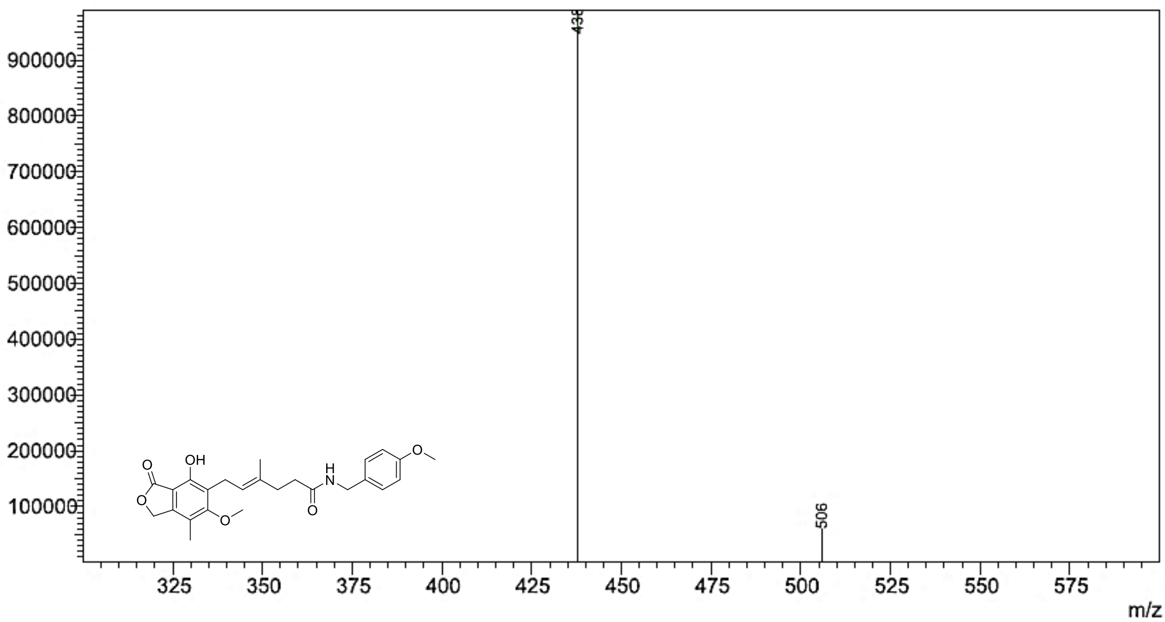

**Figure 18S.** Mass spectrum of compound **18**

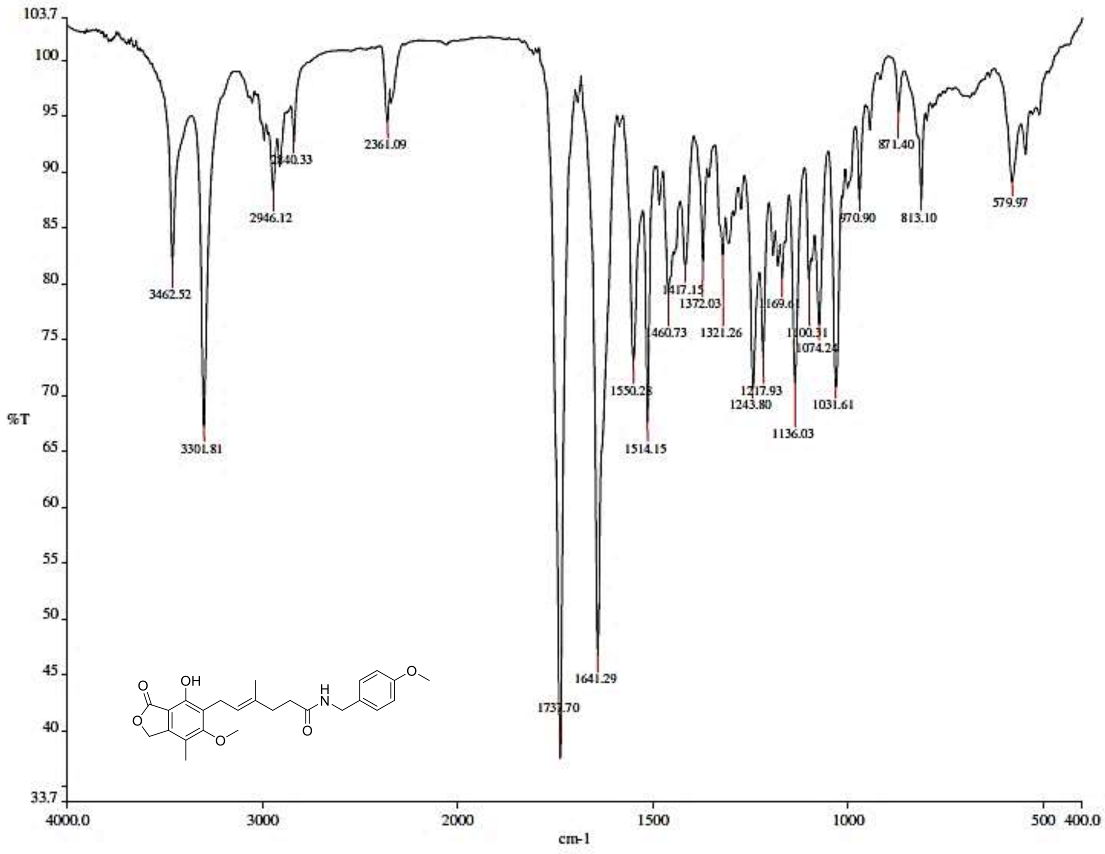


**Figure 19S.** FT-IR spectrum of compound **18**

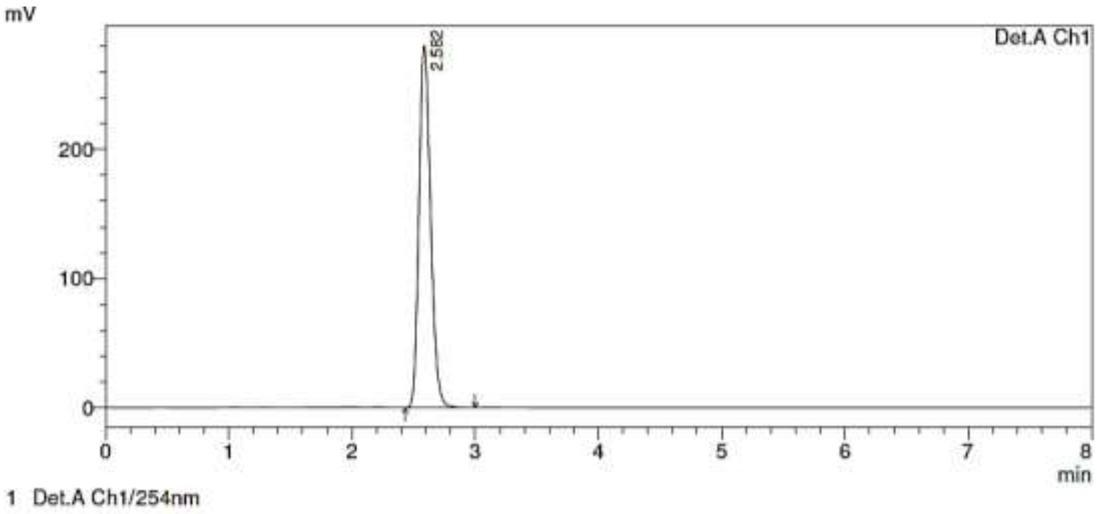


PeakTable

Detector A Ch1 254nm

| Peak# | Ret. Time | Area | Height | Area % | Height % |
|---|---|---|---|---|---|
| 1 | 2.582 | 1837216 | 280386 | 100.000 | 100.000 |
| Total | | 1837216 | 280386 | 100.000 | 100.000 |

**Figure 20S.** HPLC chromatogram of compound **18**

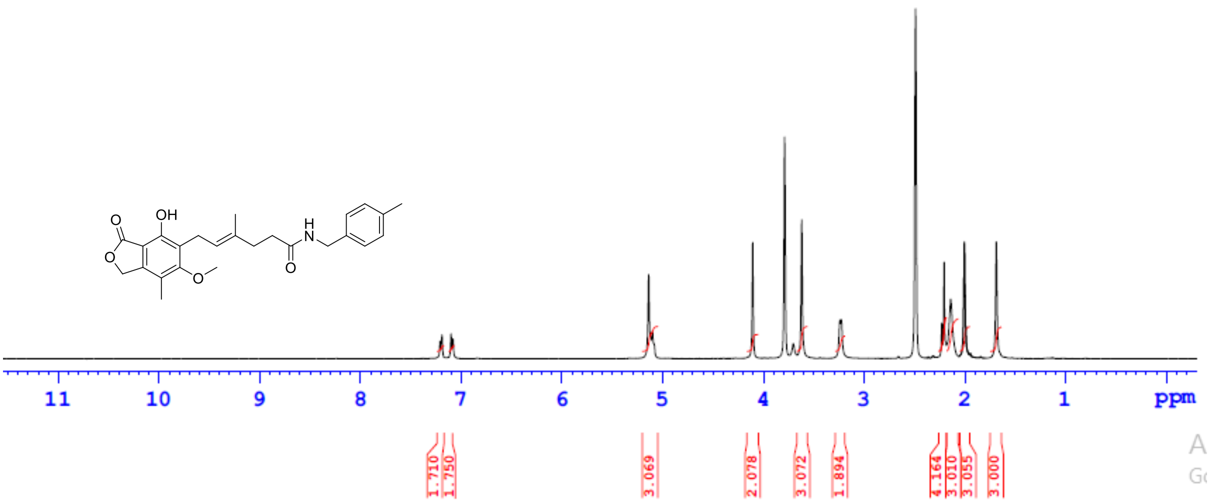


**Figure 21S.** $^{1}$H-NMR spectrum ($D_2O$ exchg.) of compound **19**

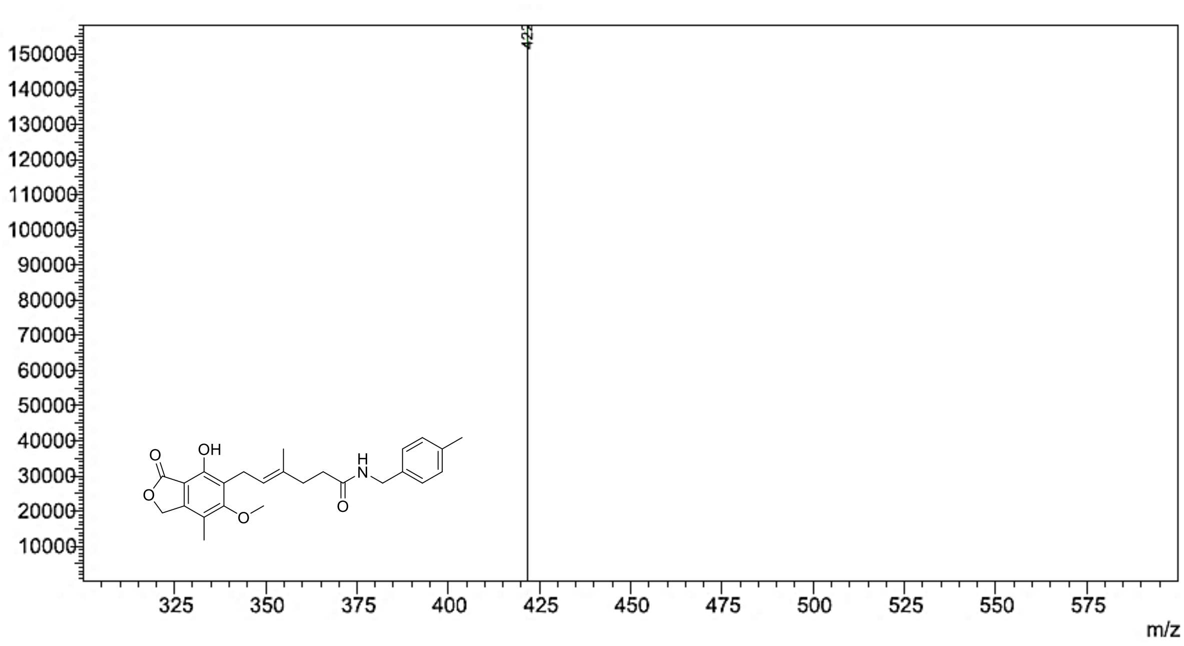


**Figure 22S.** Mass spectrum of compound **19**

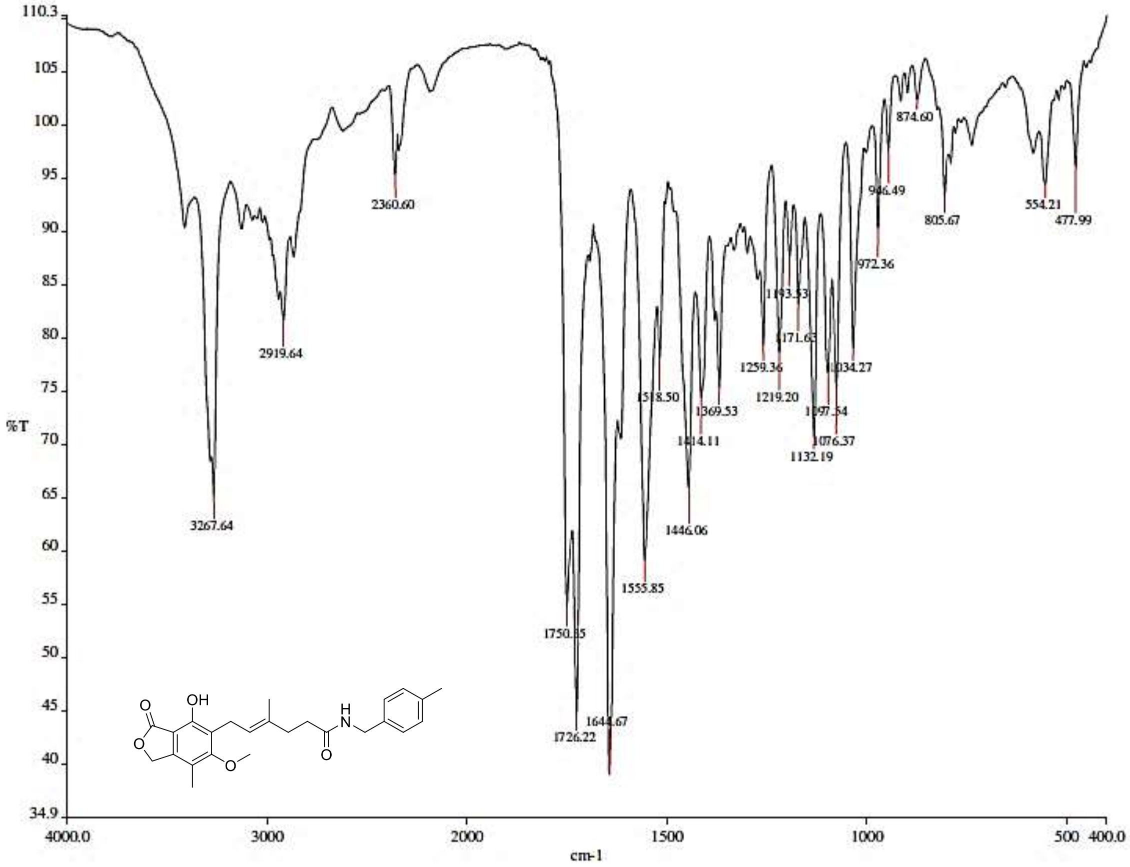


**Figure 23S.** FT-IR spectrum of compound **19**

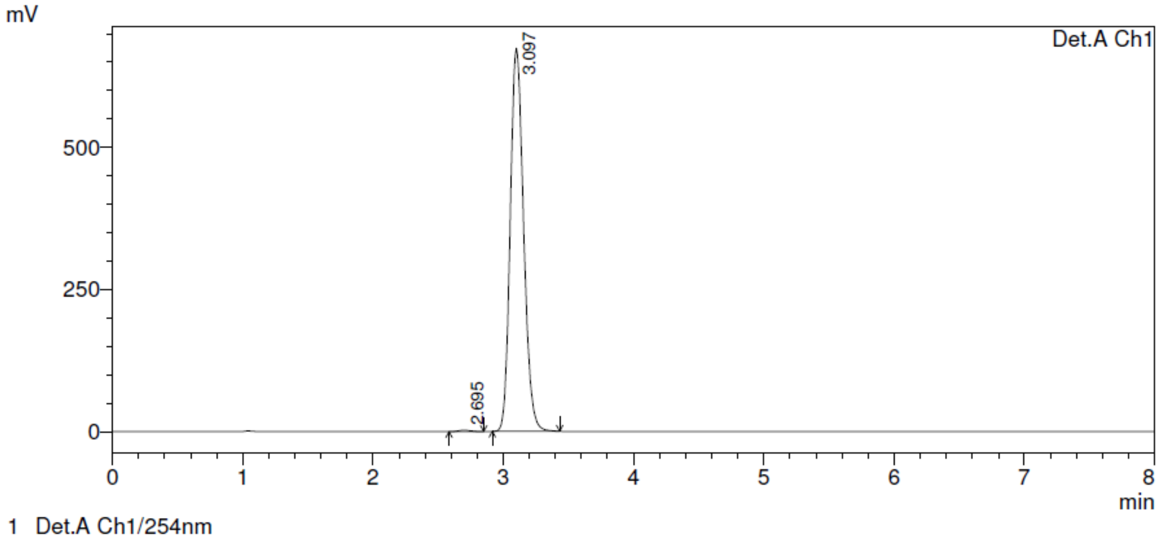


PeakTable

Detector A Ch1 254nm

| Peak# | Ret. Time | Area | Height | Area % | Height % |
|---|---|---|---|---|---|
| 1 | 2.695 | 15677 | 2233 | 0.325 | 0.330 |
| 2 | 3.097 | 4810232 | 673700 | 99.675 | 99.670 |
| Total | | 4825909 | 675934 | 100.000 | 100.000 |

**Figure 24S.** HPLC chromatogram of compound **19**

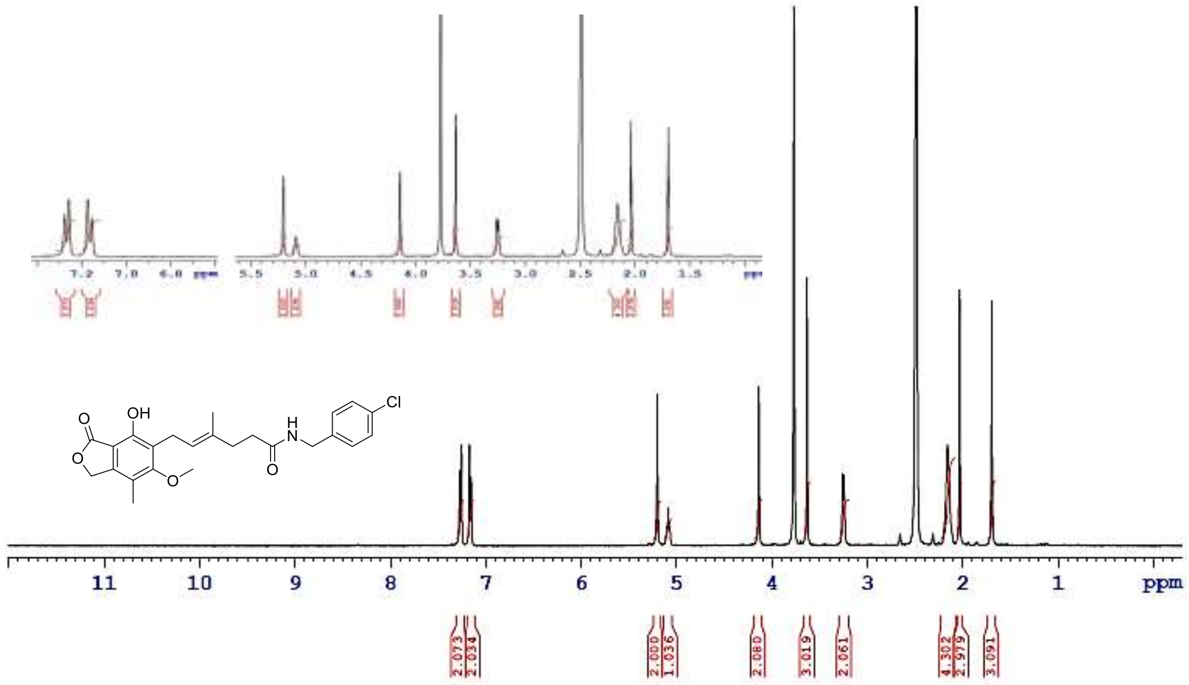

**Figure 25S.** $^1$H-NMR spectrum ($D_2O$ exchg.) of compound **20**

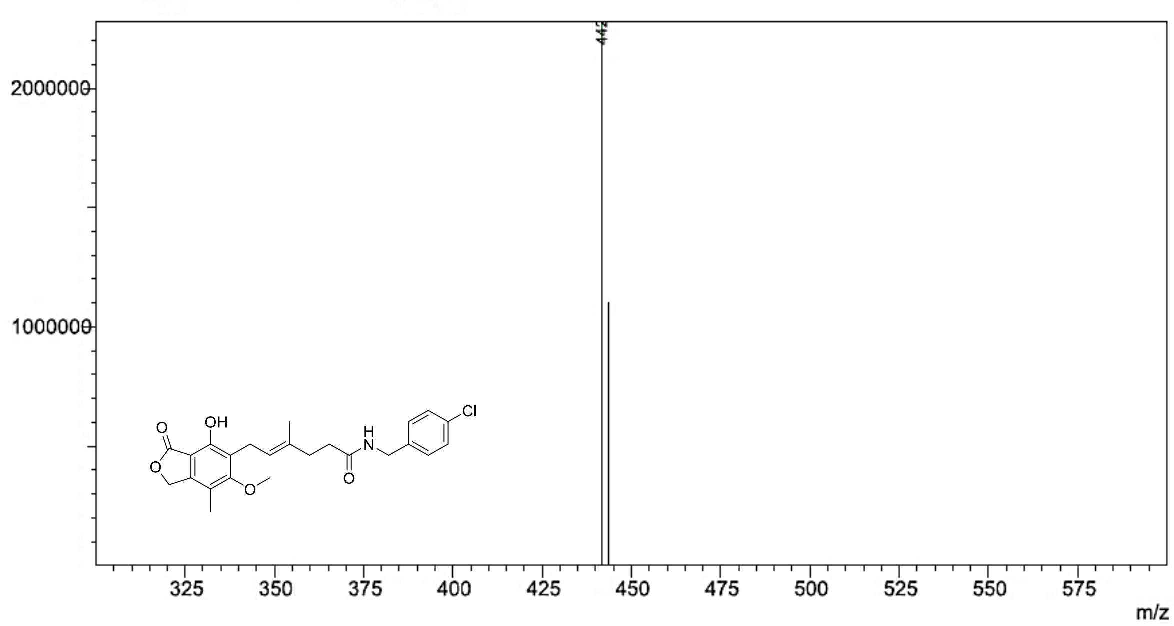

**Figure 26S.** Mass spectrum of compound **20**

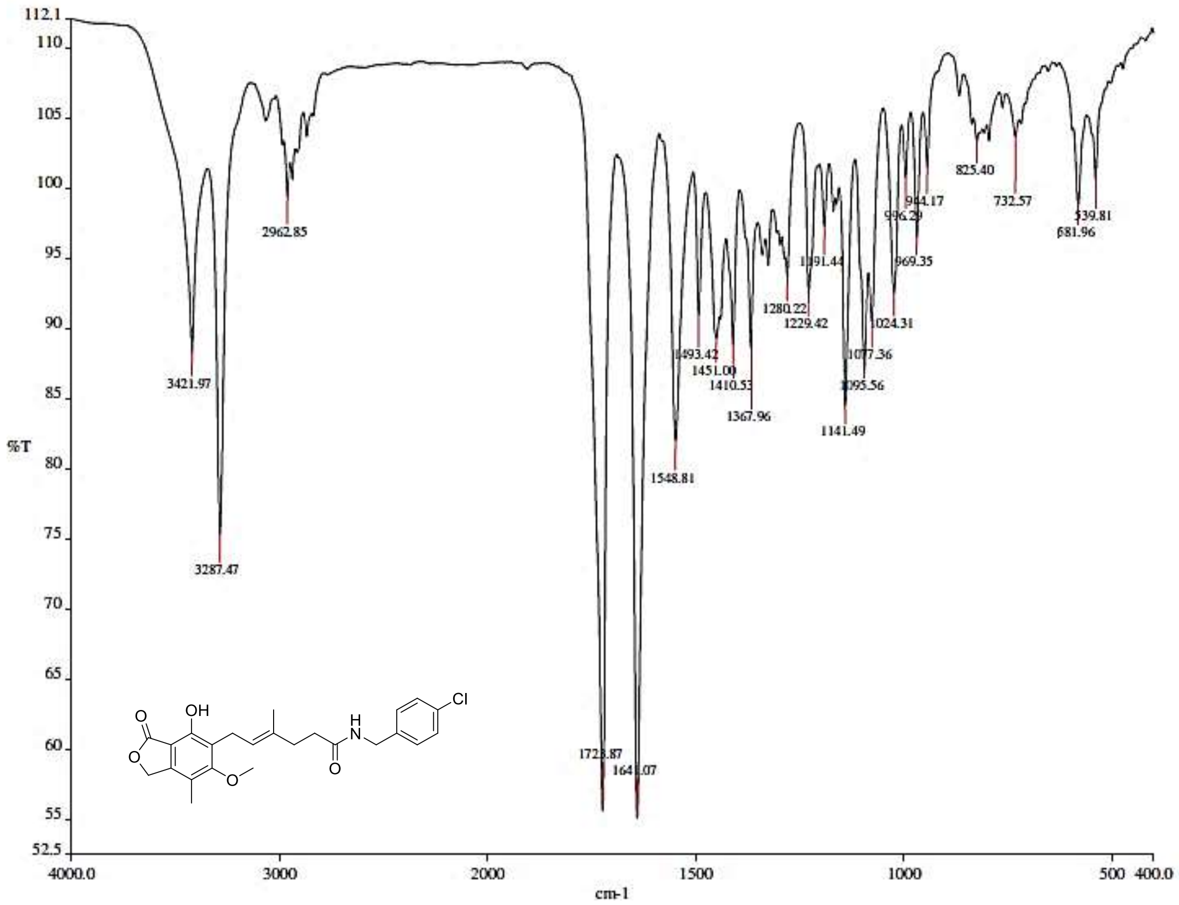


**Figure 27S.** FT-IR spectrum of compound **20**

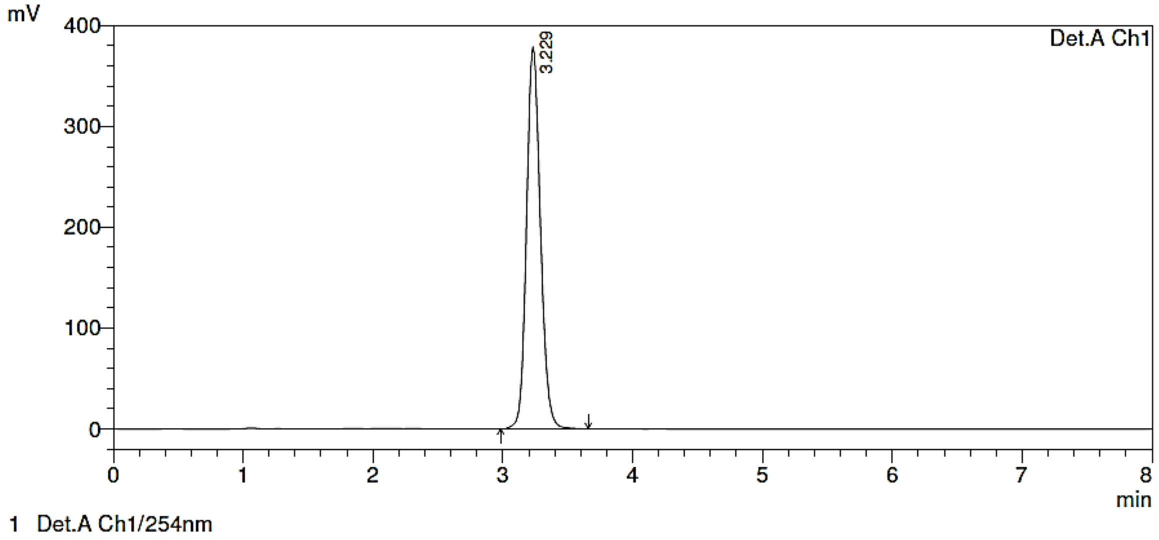


PeakTable

Detector A Ch1 254nm

| Peak# | Ret. Time | Area | Height | Area % | Height % |
|---|---|---|---|---|---|
| 1 | 3.229 | 2803722 | 378678 | 100.000 | 100.000 |
| Total | | 2803722 | 378678 | 100.000 | 100.000 |

Ac

**Figure 28S.** HPLC chromatogram of compound **20**

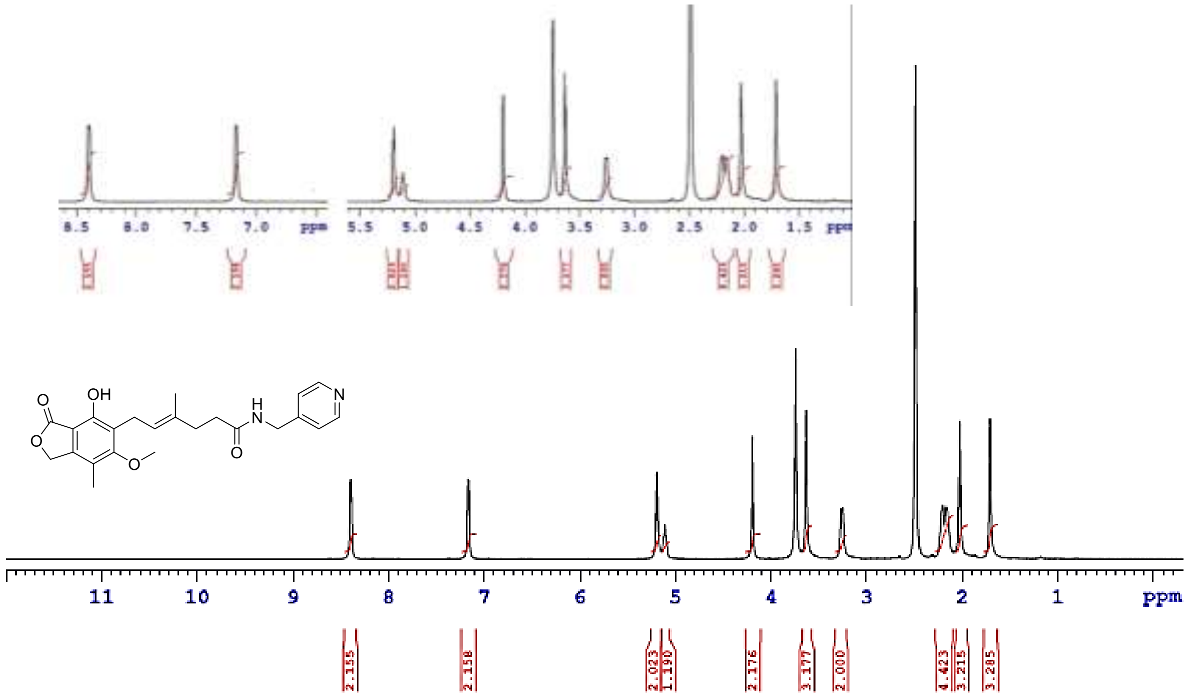


**Figure 29S.** $^{1}$H-NMR spectrum ($D_2O$ exchg.) of compound **21**

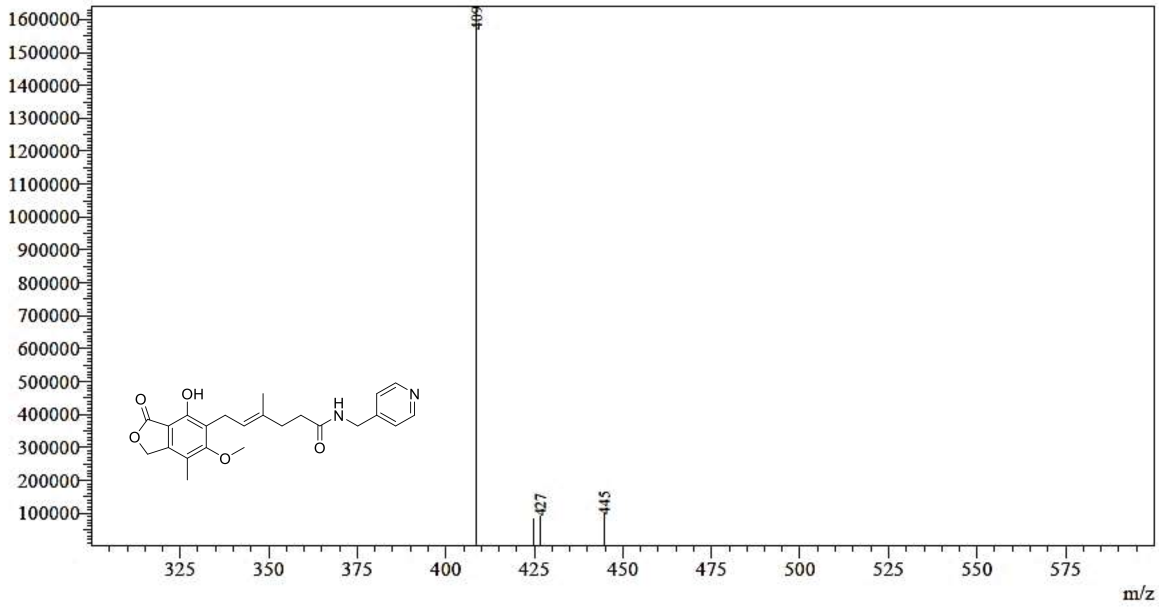


**Figure 30S.** Mass spectrum of compound **21**

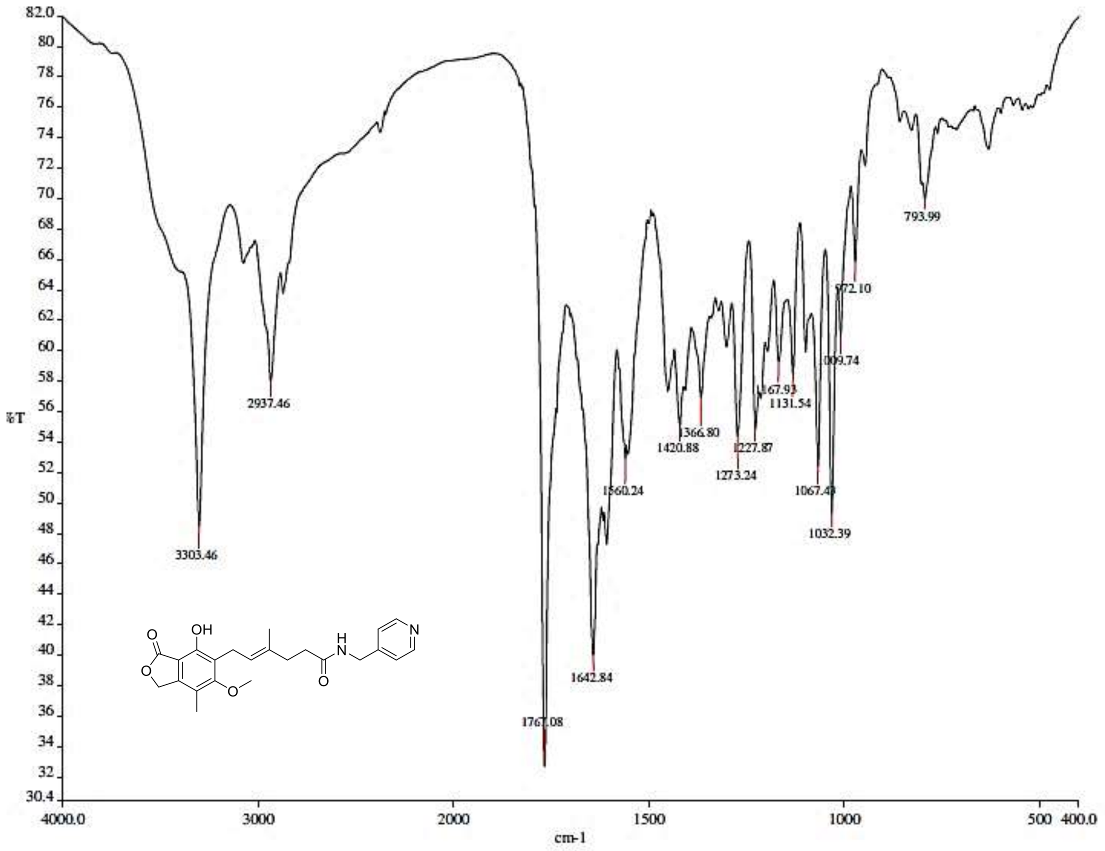


**Figure 31S.** FT-IR spectrum of compound **21**

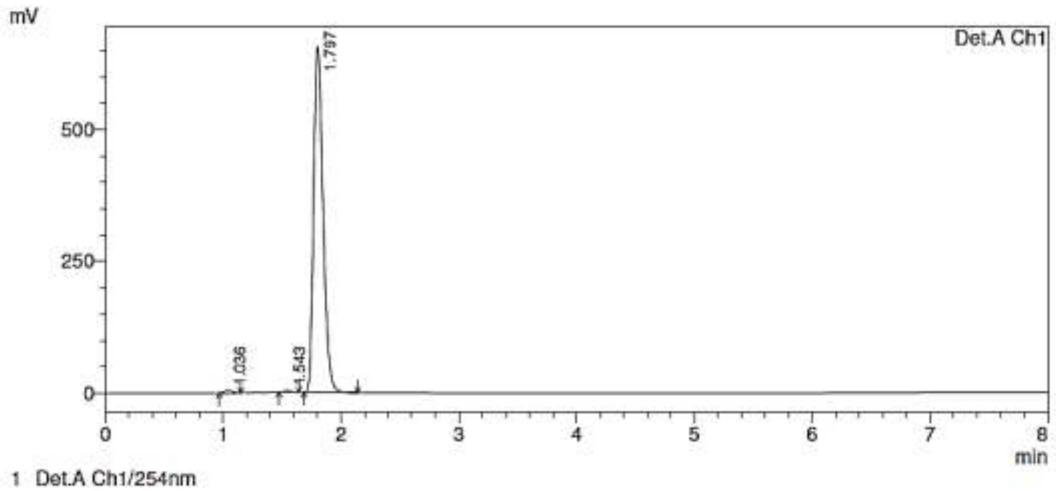


PeakTable

Detector A Ch1 254nm

| Peak# | Ret. Time | Area | Height | Area % | Height % |
|---|---|---|---|---|---|
| 1 | 1.036 | 19538 | 4140 | 0.530 | 0.625 |
| 2 | 1.543 | 9541 | 1753 | 0.259 | 0.264 |
| 3 | 1.797 | 3659242 | 656943 | 99.212 | 99.111 |
| Total | | 3688321 | 662835 | 100.000 | 100.000 |

**Figure 32S.** HPLC chromatogram of compound **21**

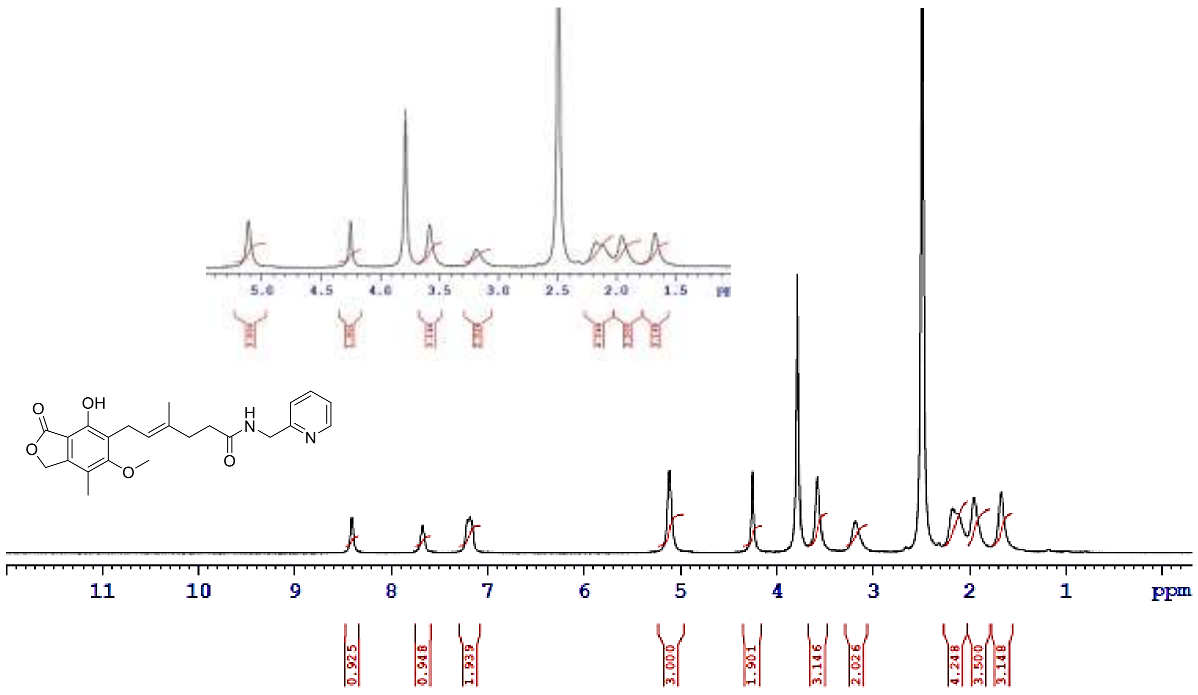


**Figure 33S.** $^{1}$H-NMR spectrum ($D_2O$ exchg.) of compound **22**

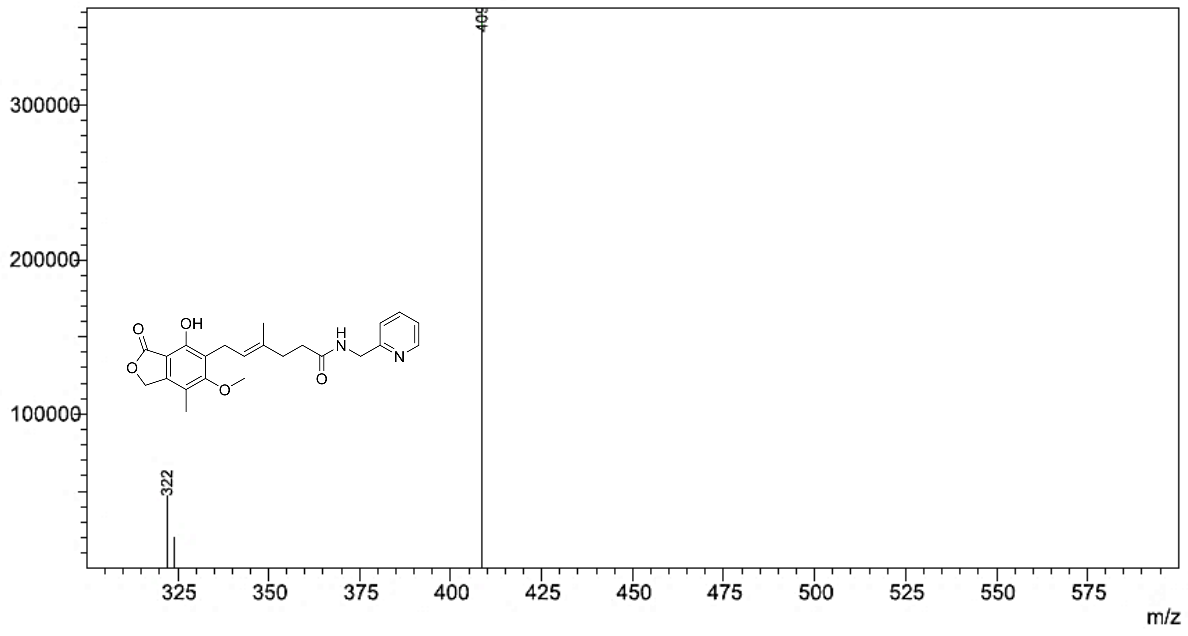


**Figure 34S.** Mass spectrum of compound **22**

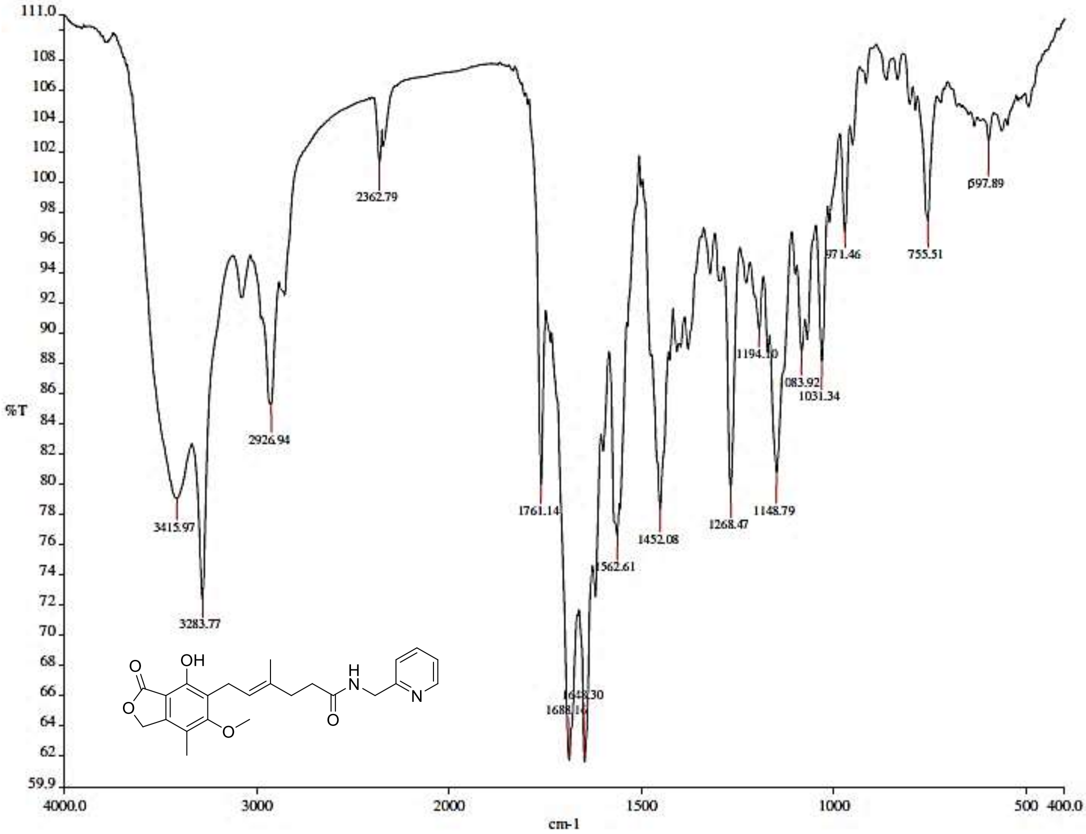


**Figure 35S.** FT-IR spectrum of compound **22**

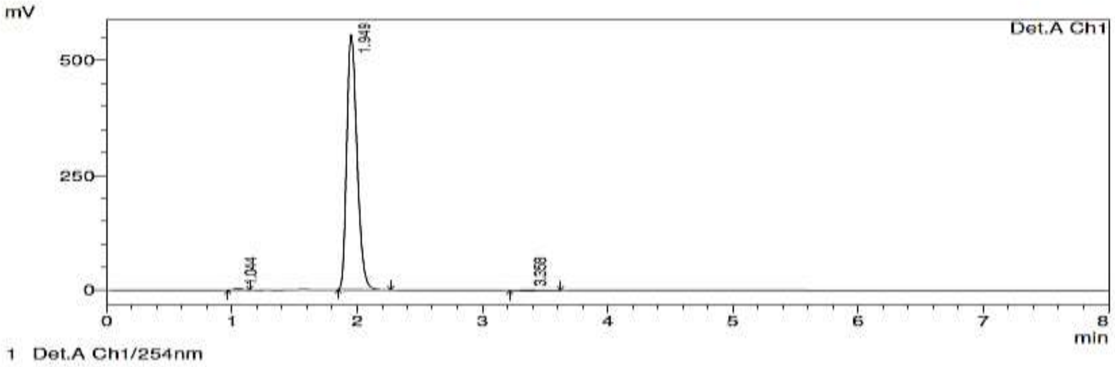


PeakTable

Detector A Ch1 254nm

| Peak# | Ret. Time | Area | Height | Area % | Height % |
|---|---|---|---|---|---|
| 1 | 1.044 | 16970 | 3568 | 0.532 | 0.637 |
| 2 | 1.949 | 3165941 | 555397 | 99.174 | 99.195 |
| 3 | 3.358 | 9385 | 941 | 0.294 | 0.168 |
| Total | | 3192296 | 559906 | 100.000 | 100.000 |

**Figure 36S.** HPLC chromatogram of compound **22**

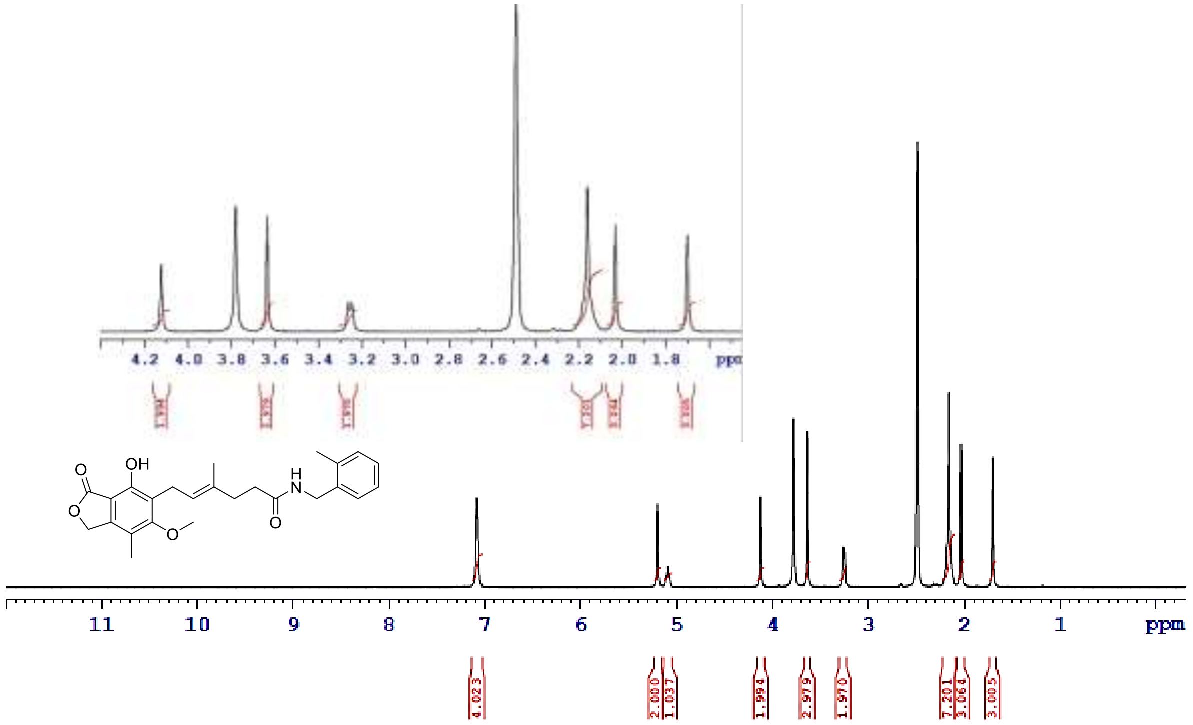

**Figure 37S.** $^1$H-NMR spectrum ($D_2O$ exchg.) of compound **23**

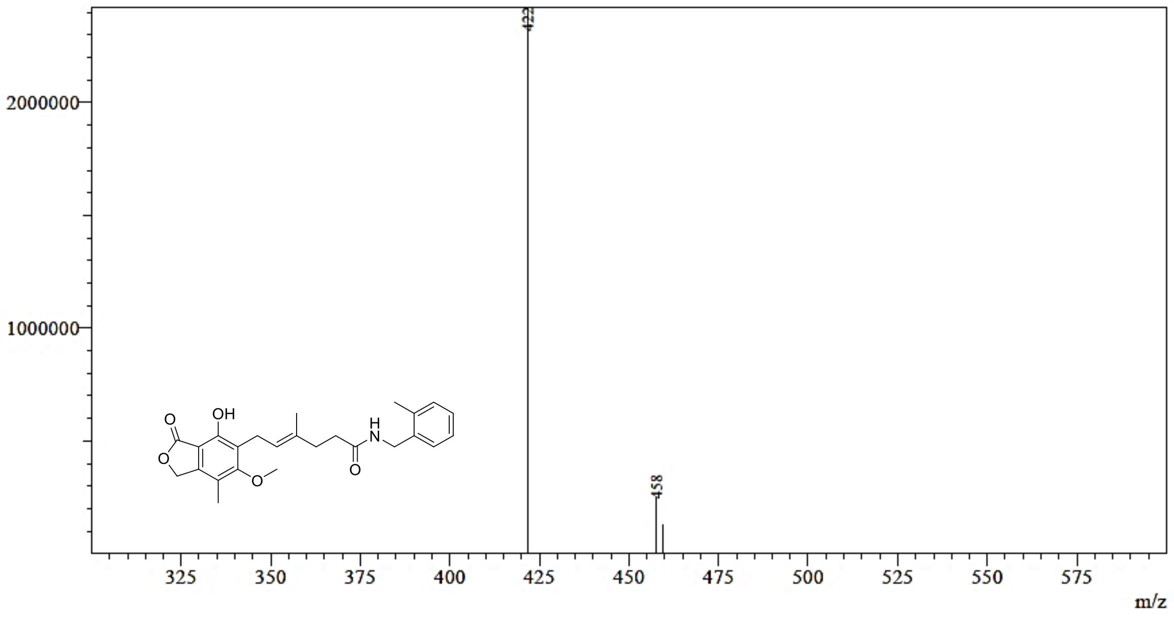

**Figure 38S.** Mass spectrum of compound **23**

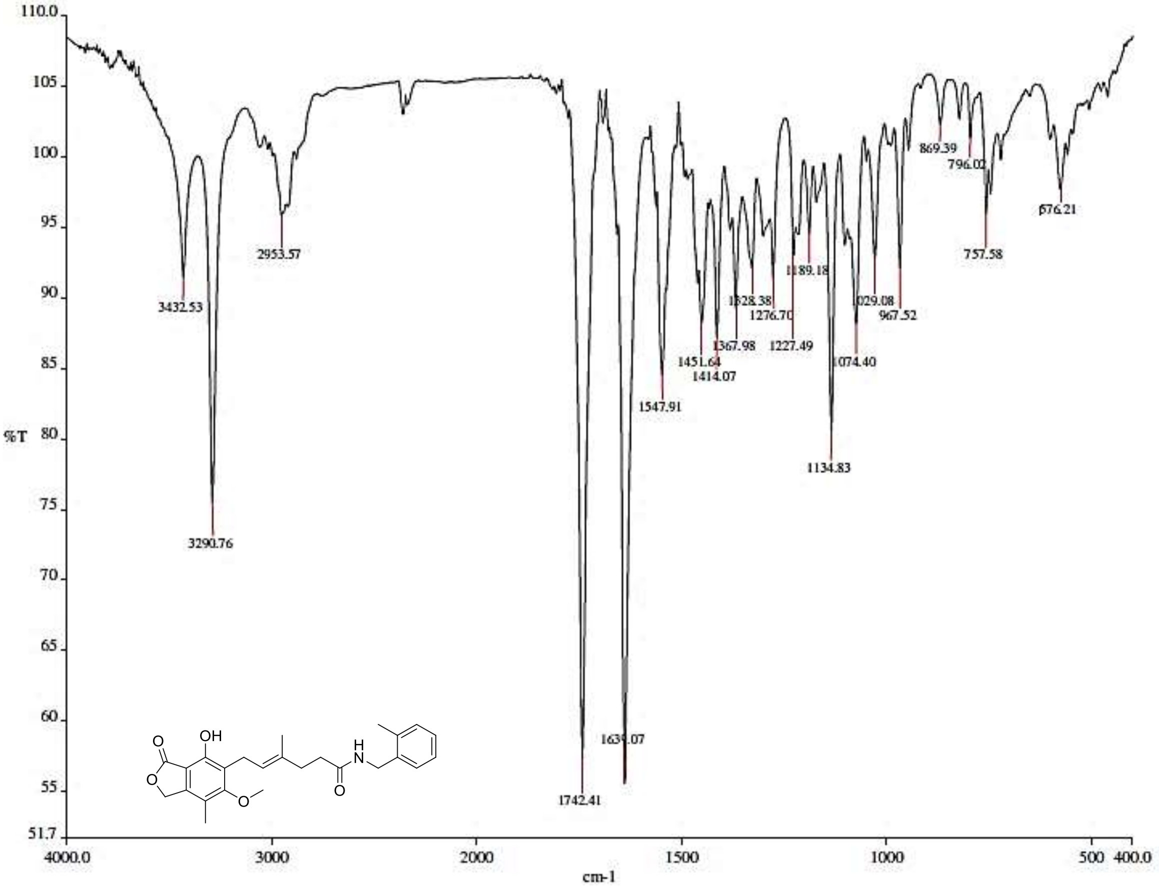


**Figure 39S.** FT-IR spectrum of compound **23**

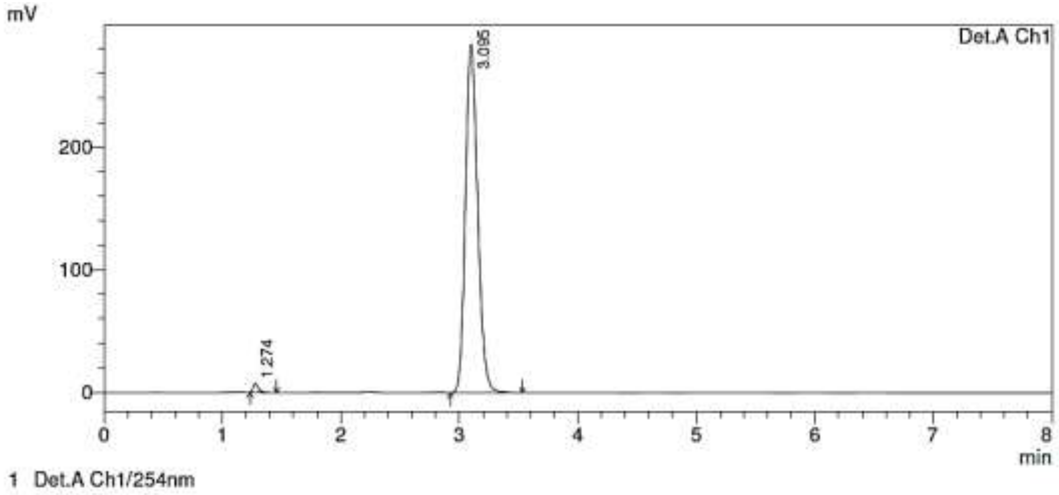


PeakTable

Detector A Ch1 254nm

| Peak# | Ret. Time | Area | Height | Area % | Height % |
|---|---|---|---|---|---|
| 1 | 1.274 | 20388 | 6994 | 0.989 | 2.405 |
| 2 | 3.095 | 2041335 | 283799 | 99.011 | 97.595 |
| Total | | 2061723 | 290793 | 100.000 | 100.000 |

Act
Go t

**Figure 40S.** HPLC chromatogram of compound **23**

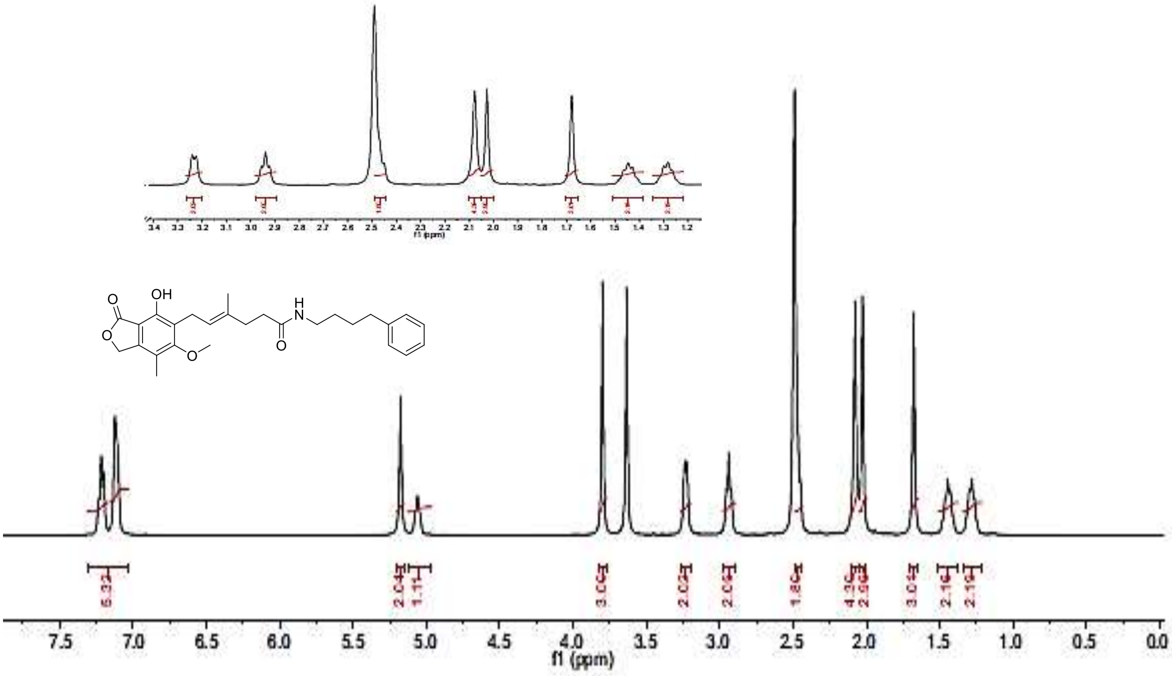


**Figure 41S.** $^1$H-NMR spectrum ($D_2O$ exchg.) of compound **24**

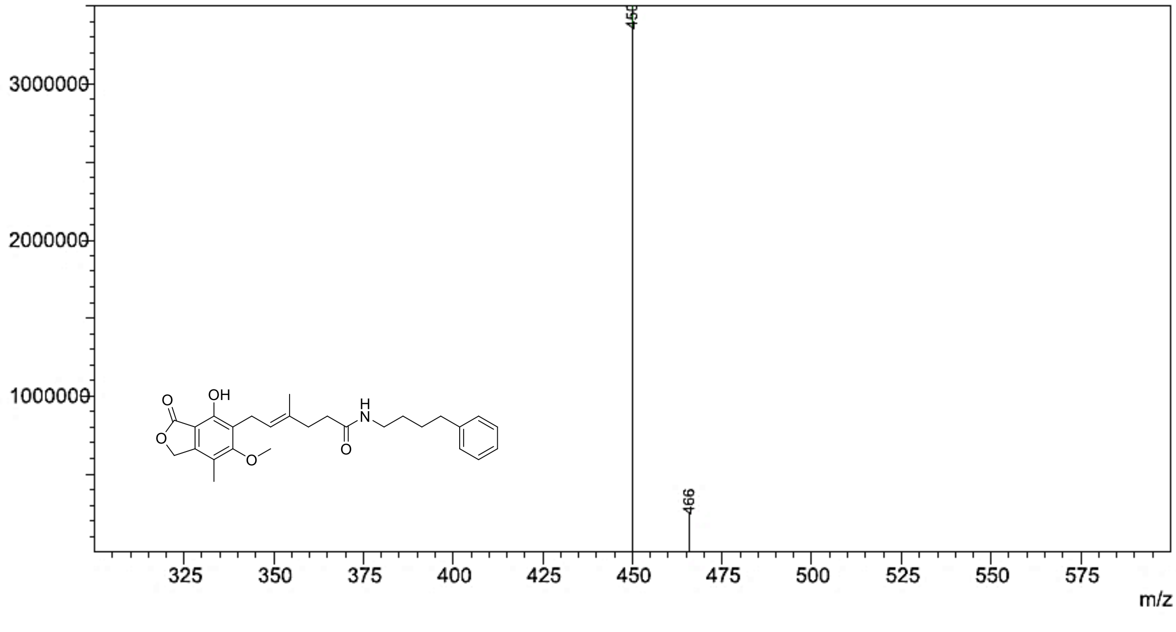


**Figure 42S.** Mass spectrum of compound **24**

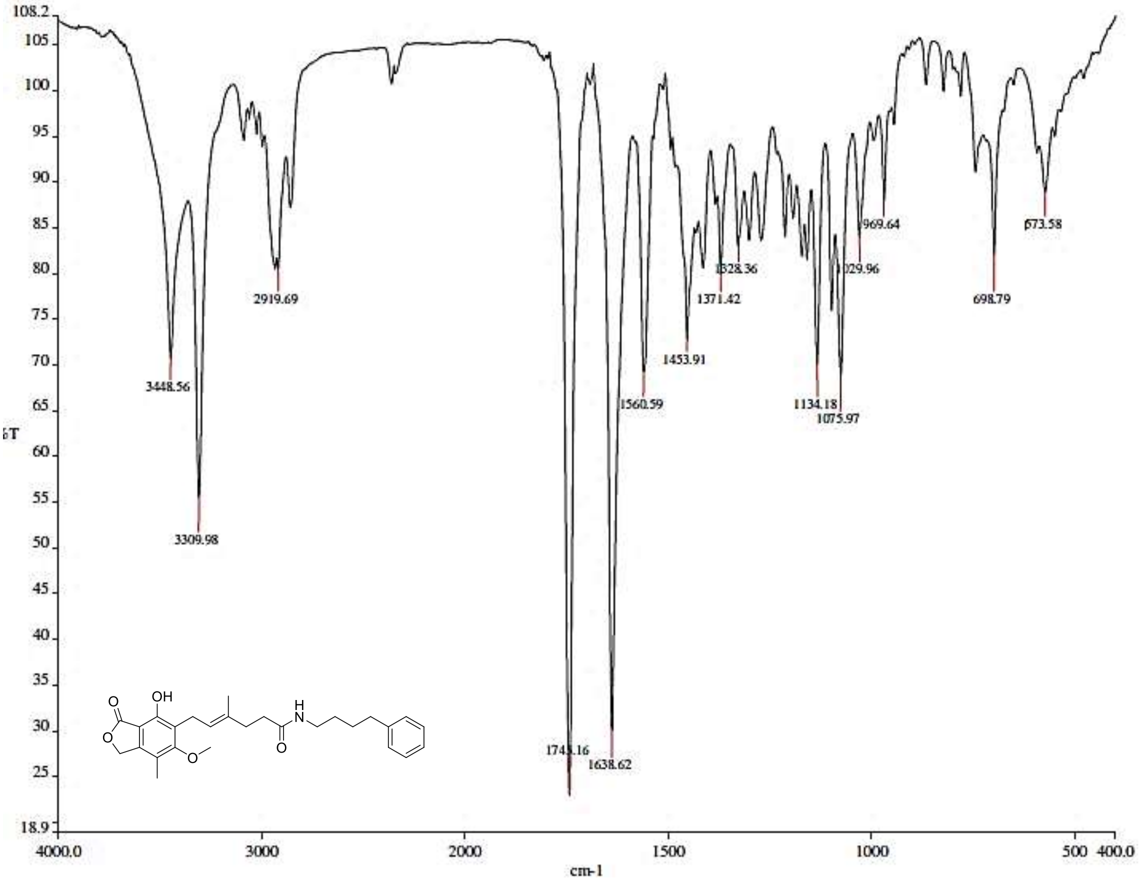


**Figure 43S.** FT-IR spectrum of compound **24**

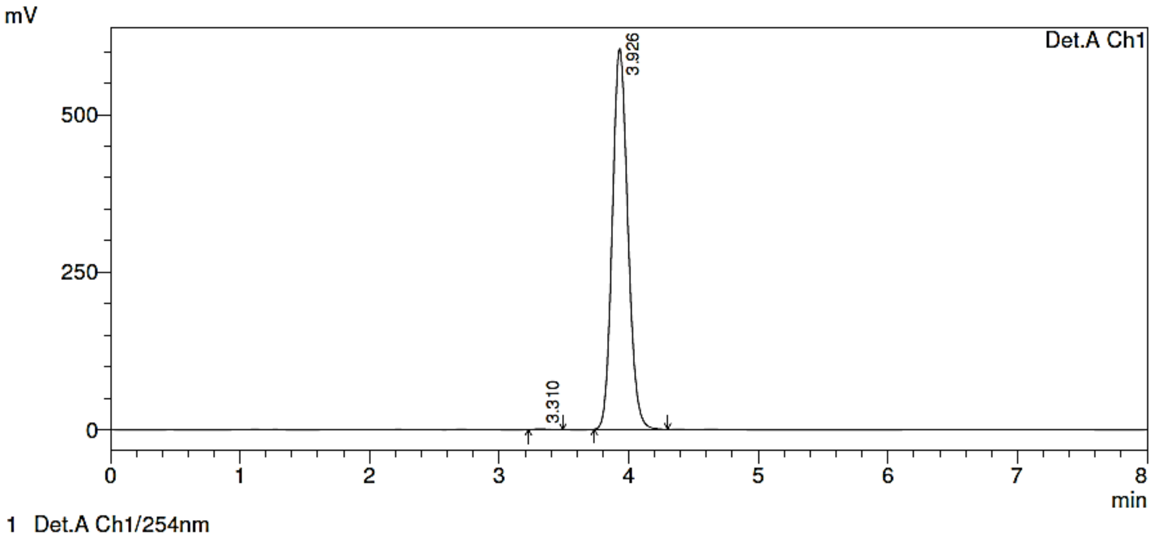


PeakTable

Detector A Ch1 254nm

| Peak# | Ret. Time | Area | Height | Area % | Height % |
|---|---|---|---|---|---|
| 1 | 3.310 | 8229 | 1215 | 0.163 | 0.201 |
| 2 | 3.926 | 5033605 | 604121 | 99.837 | 99.799 |
| Total | | 5041834 | 605336 | 100.000 | 100.000 |

A
G

**Figure 44S.** HPLC chromatogram of compound **24**

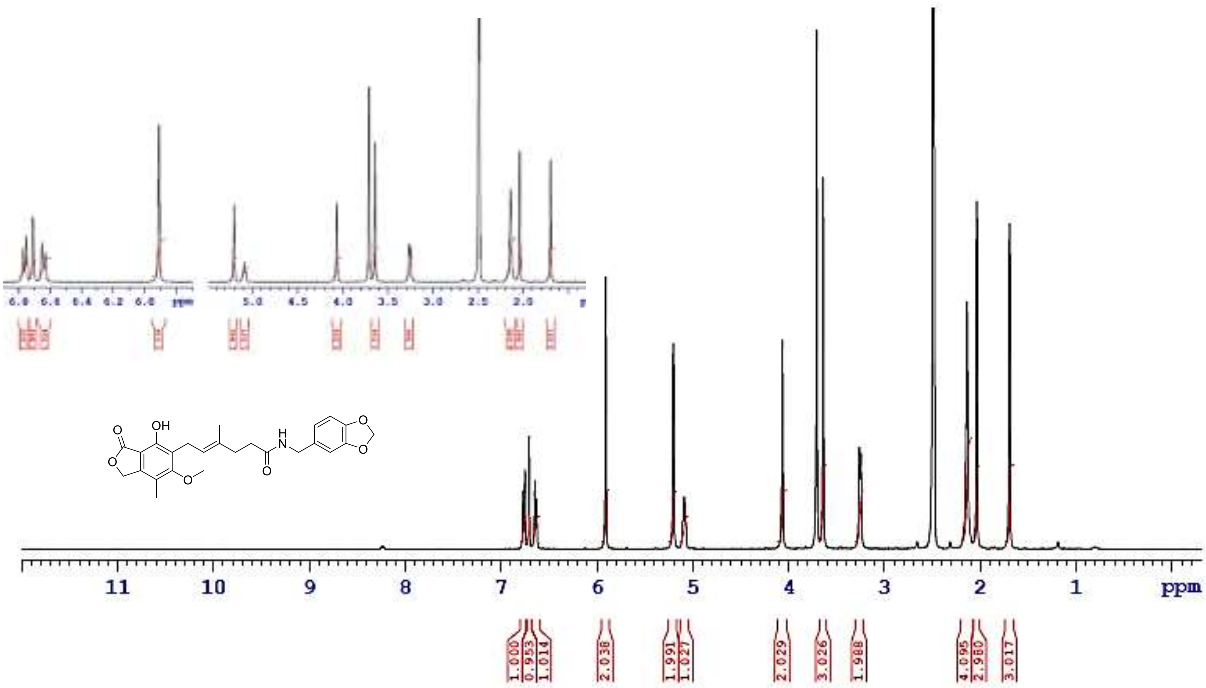


**Figure 45S.** $^{1}$H-NMR spectrum ($D_2O$ exchg.) of compound **25**

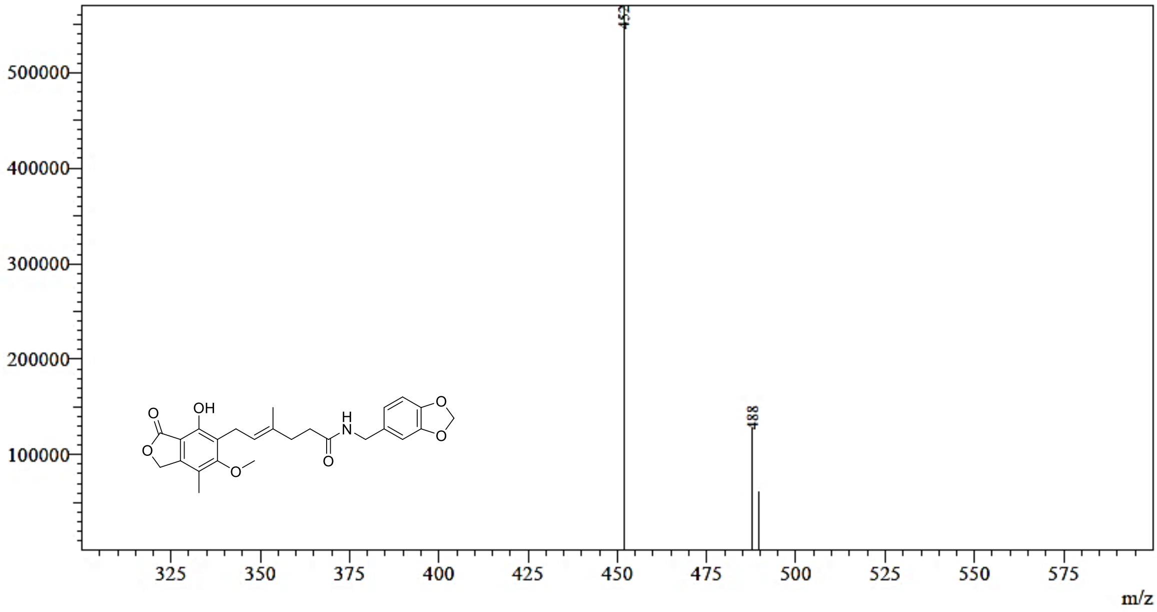


**Figure 46S.** Mass spectrum of compound **25**

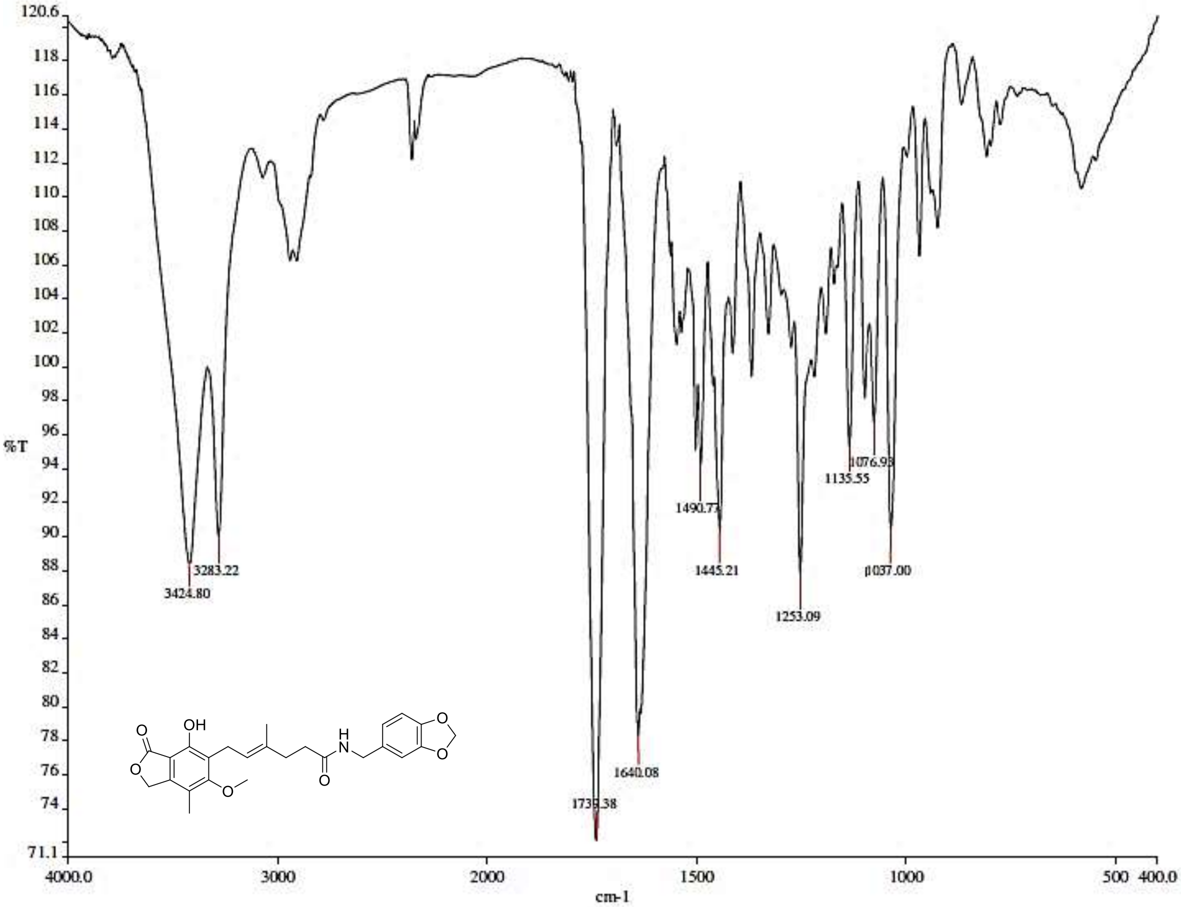


**Figure 47S.** FT-IR spectrum of compound **25**

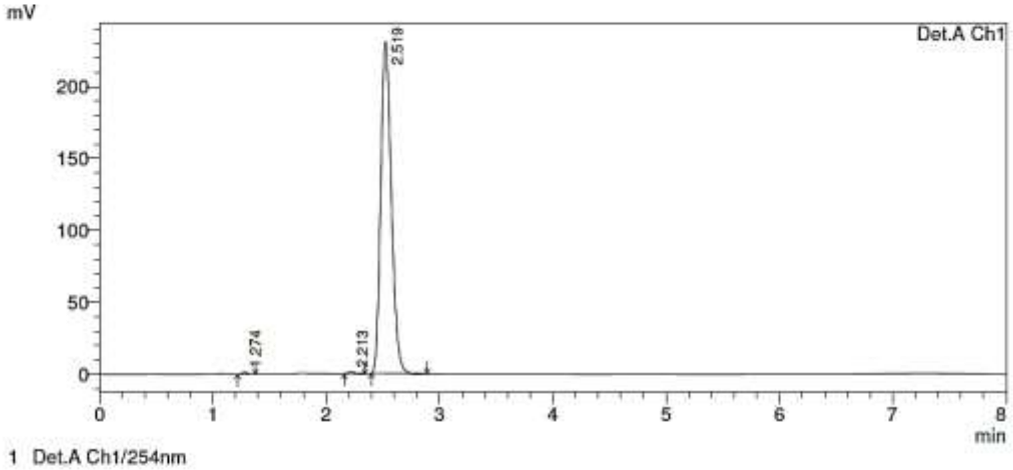


PeakTable

Detector A Ch1 254nm

| Peak# | Ret. Time | Area | Height | Area % | Height % |
|---|---|---|---|---|---|
| 1 | 1.274 | 4442 | 1435 | 0.295 | 0.615 |
| 2 | 2.213 | 5945 | 1165 | 0.394 | 0.499 |
| 3 | 2.519 | 1497425 | 230875 | 99.311 | 98.887 |
| Total | | 1507812 | 233474 | 100.000 | 100.000 |

**Figure 48S.** HPLC chromatogram of compound **25**

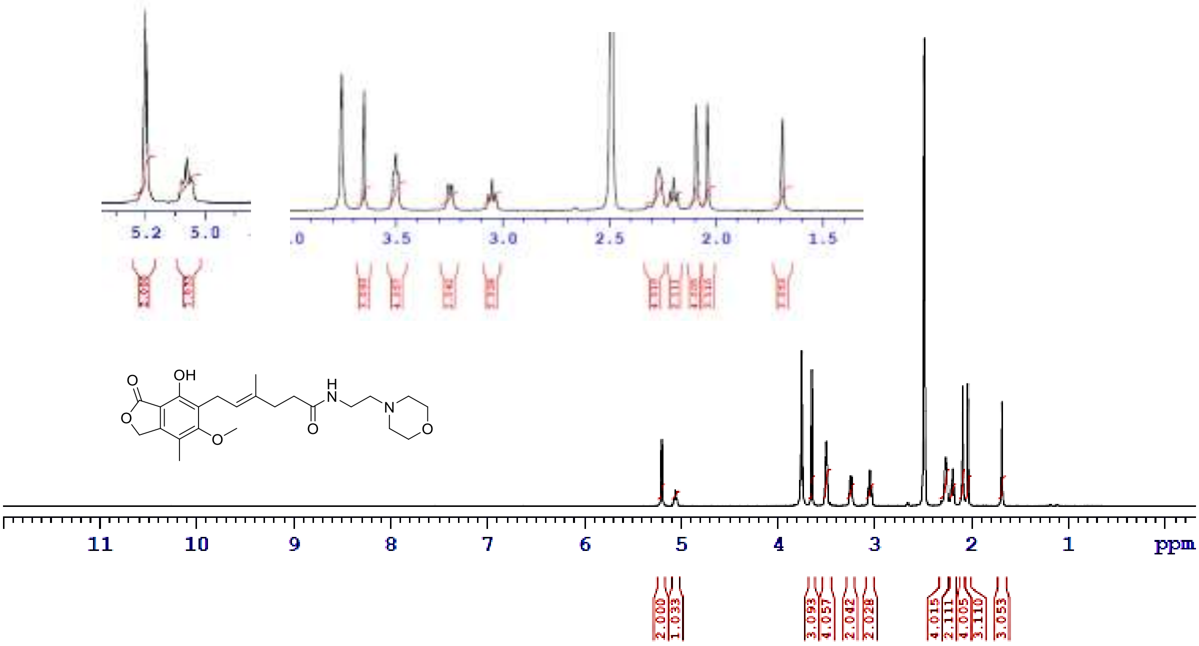


**Figure 49S.** $^{1}$H-NMR spectrum ($D_2O$ exchg.) of compound **26**

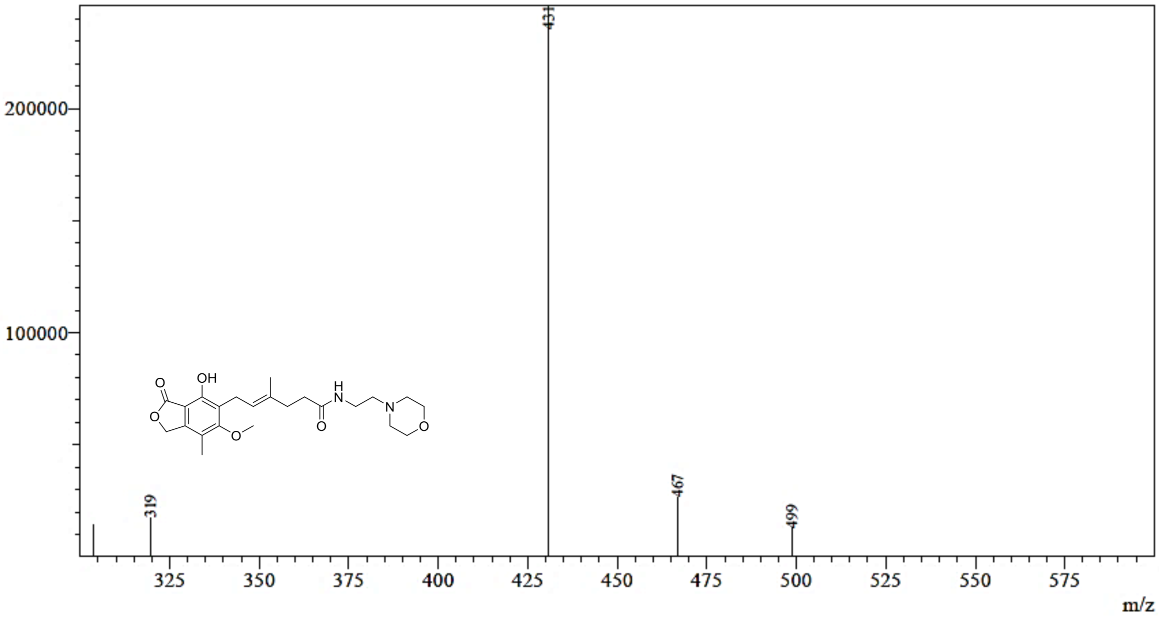


**Figure 50S.** Mass spectrum of compound **26**

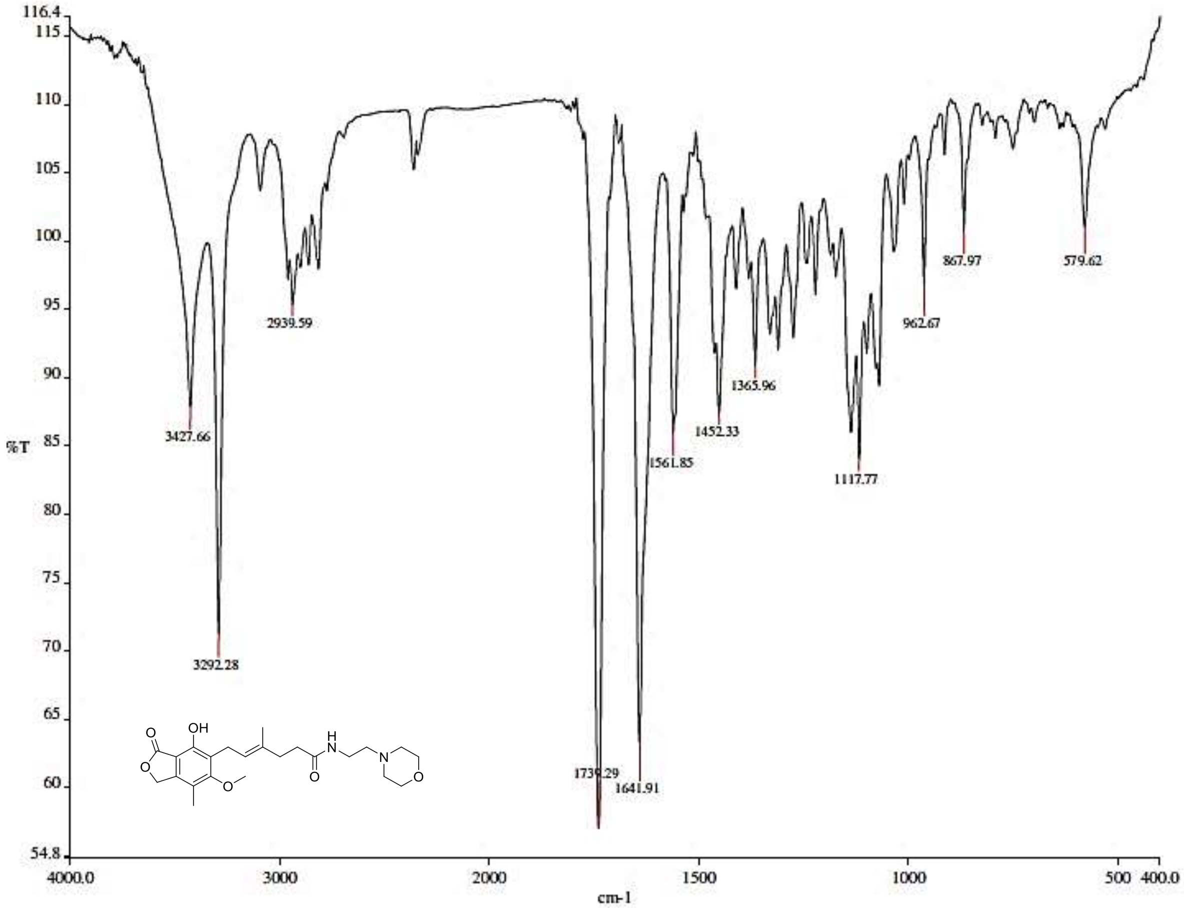


**Figure 51S.** FT-IR spectrum of compound **26**

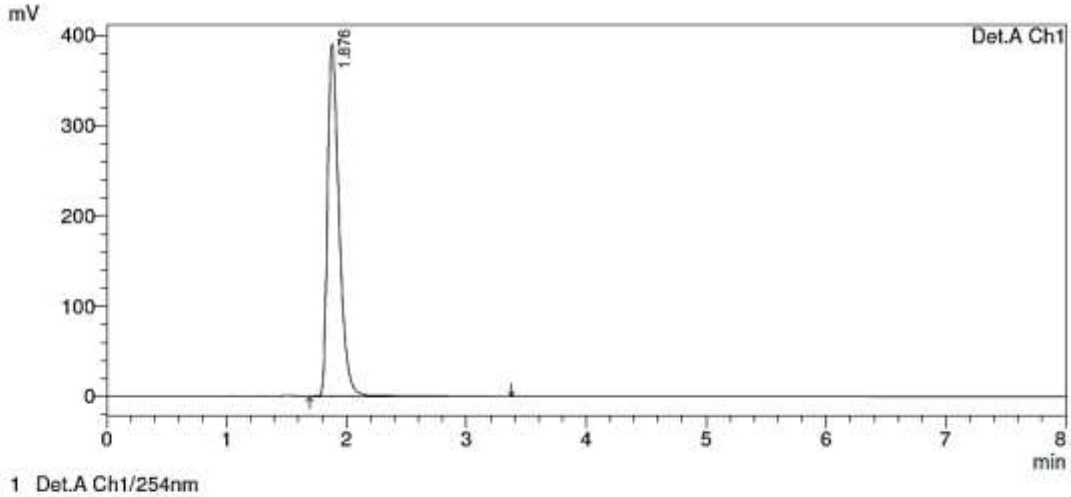


PeakTable

Detector A Ch1 254nm

| Peak# | Ret. Time | Area | Height | Area % | Height % |
|---|---|---|---|---|---|
| 1 | 1.876 | 2623697 | 390010 | 100.000 | 100.000 |
| Total | | 2623697 | 390010 | 100.000 | 100.000 |

L,

**Figure 52S.** HPLC chromatogram of compound **26**

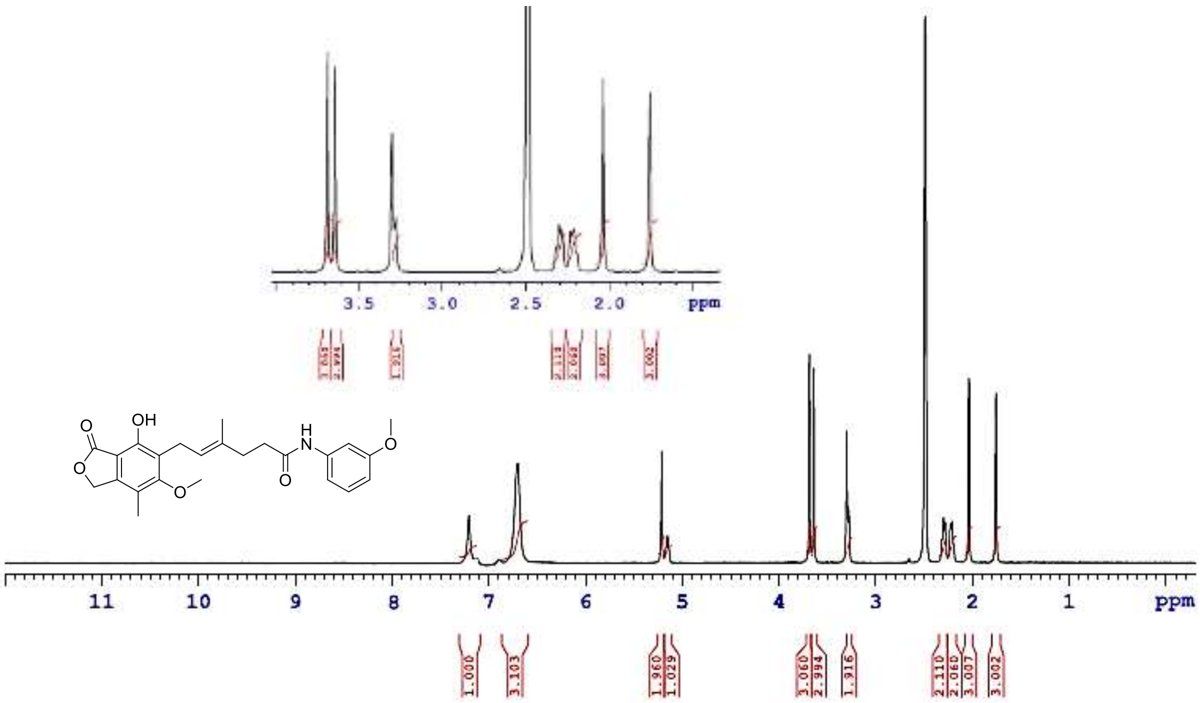

**Figure 53S.** $^{1}$H-NMR spectrum ($D_2O$ exchg.) of compound **27**

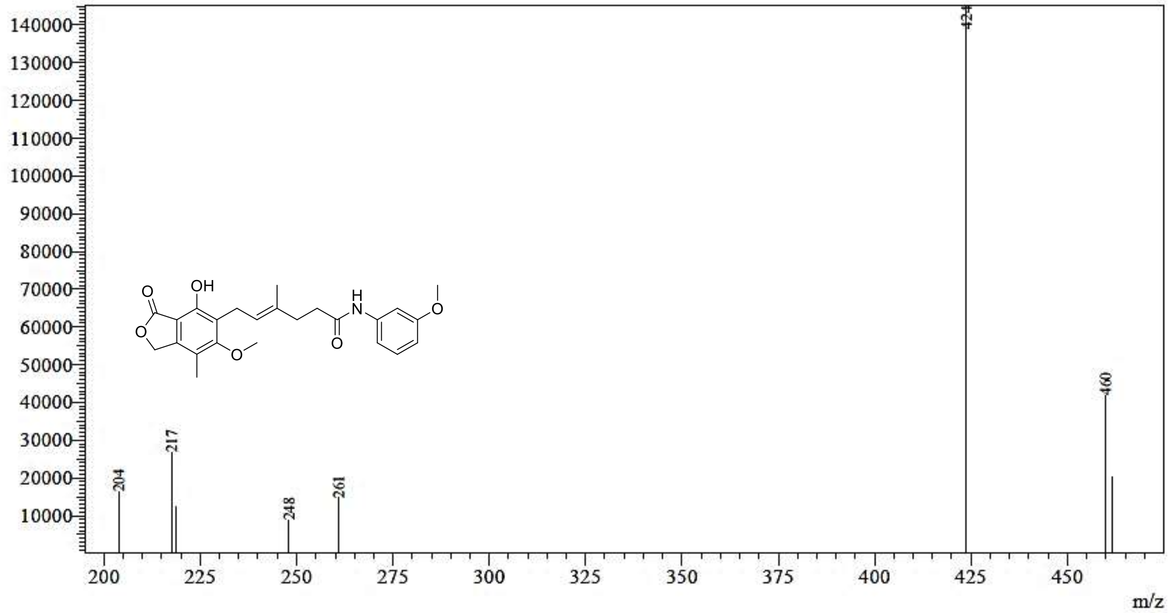

**Figure 54S.** Mass spectrum of compound **27**

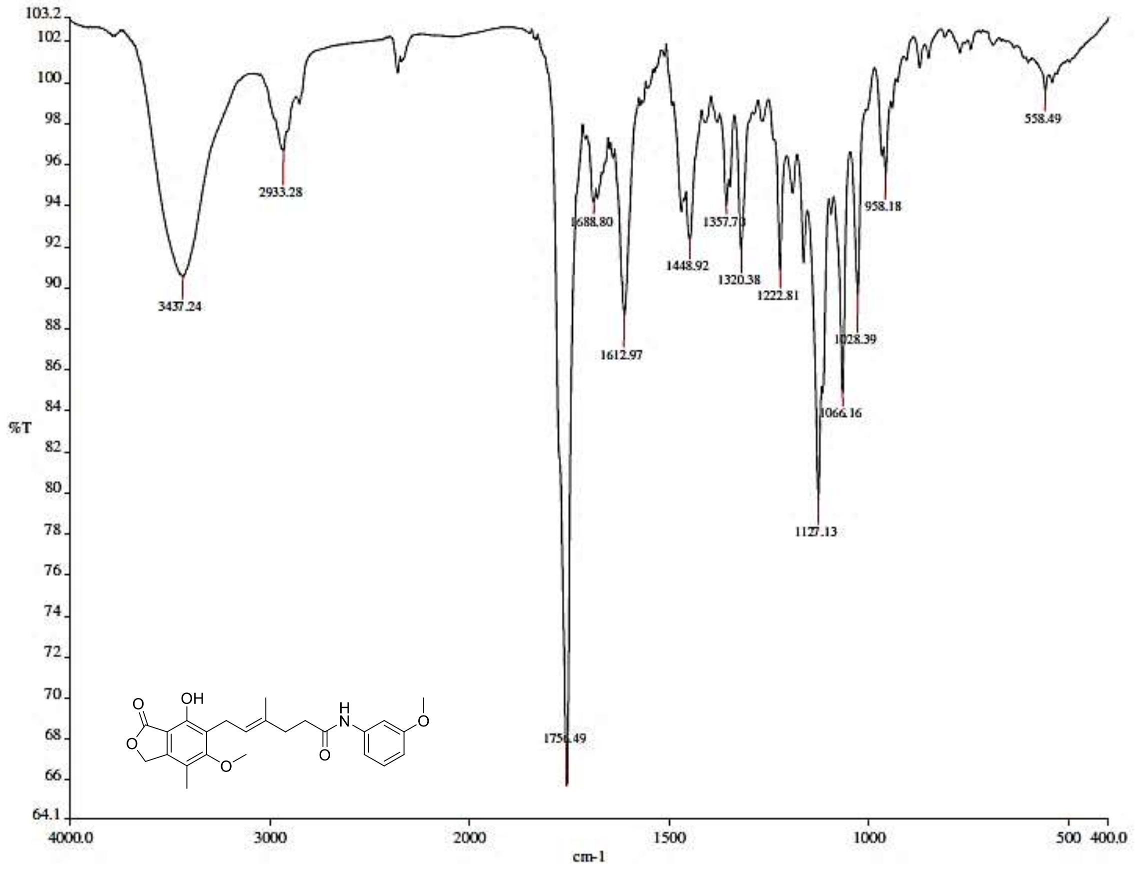


**Figure 55S.** FT-IR spectrum of compound **27**

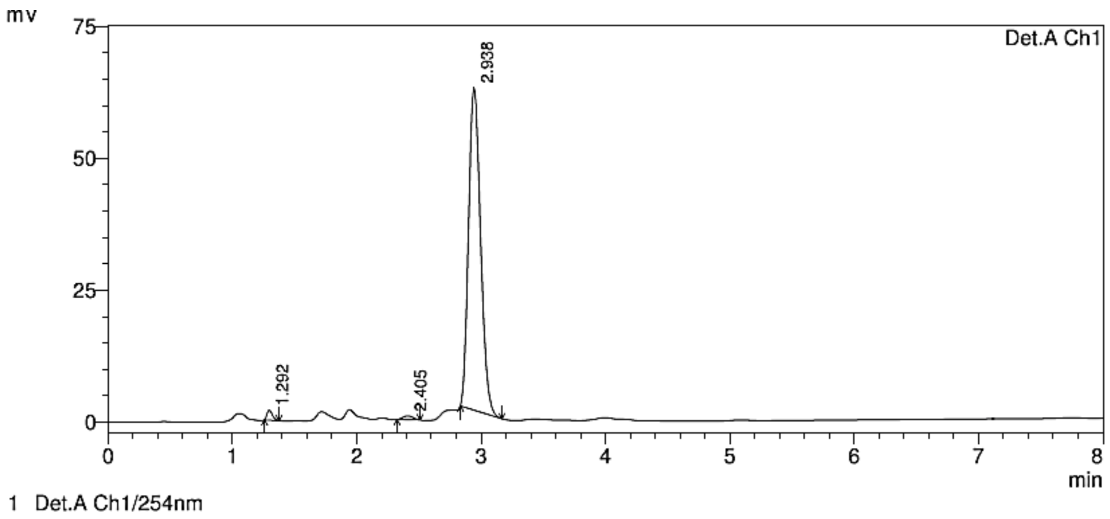


PeakTable

Detector A Ch1 254nm

| Peak# | Ret. Time | Area | Height | Area % | Height % |
|---|---|---|---|---|---|
| 1 | 1.292 | 5572 | 1867 | 1.335 | 2.933 |
| 2 | 2.405 | 4287 | 724 | 1.027 | 1.137 |
| 3 | 2.938 | 407628 | 61064 | 97.638 | 95.930 |
| Total | | 417488 | 63655 | 100.000 | 100.000 |

**Figure 56S.** HPLC chromatogram of compound **27**

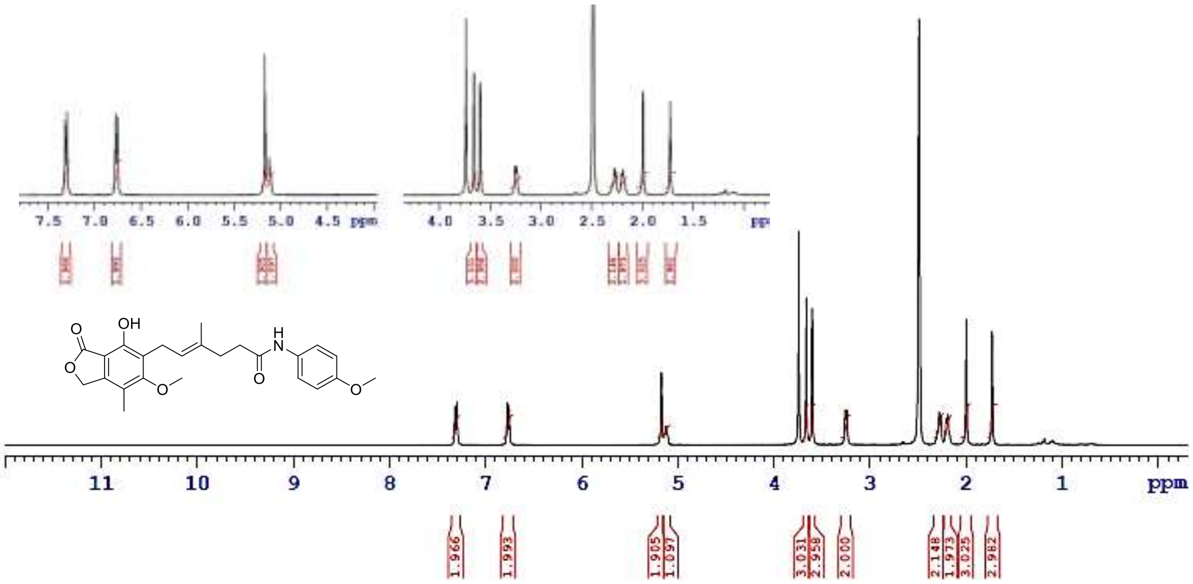

**Figure 57S.** $^{1}$H-NMR spectrum ($D_2O$ exchg.) of compound **28**

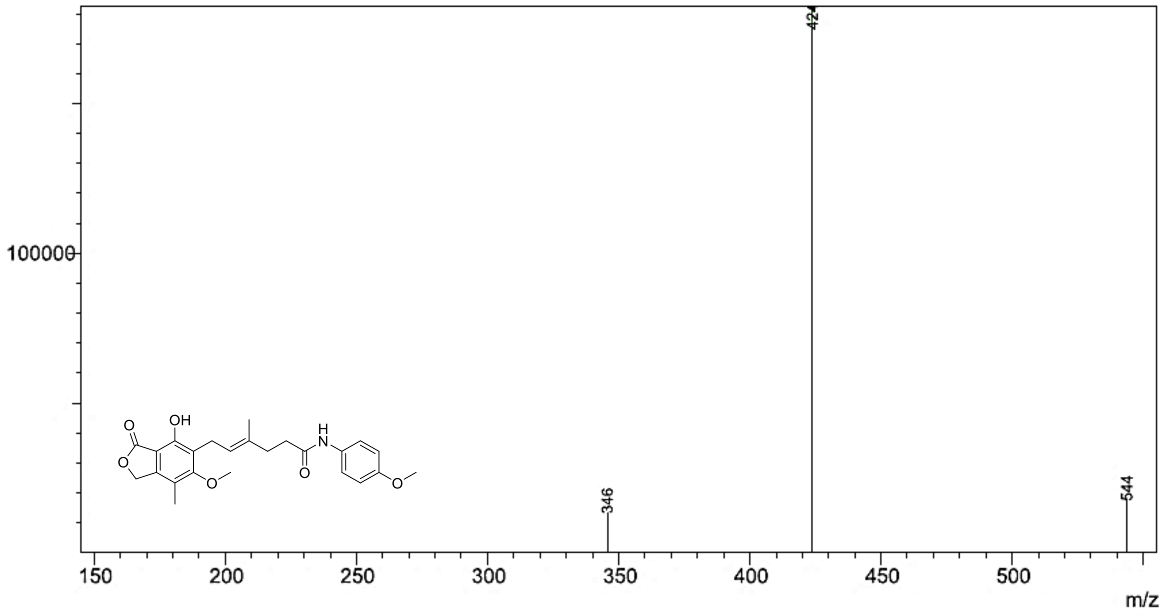

**Figure 58S.** Mass spectrum of compound **28**

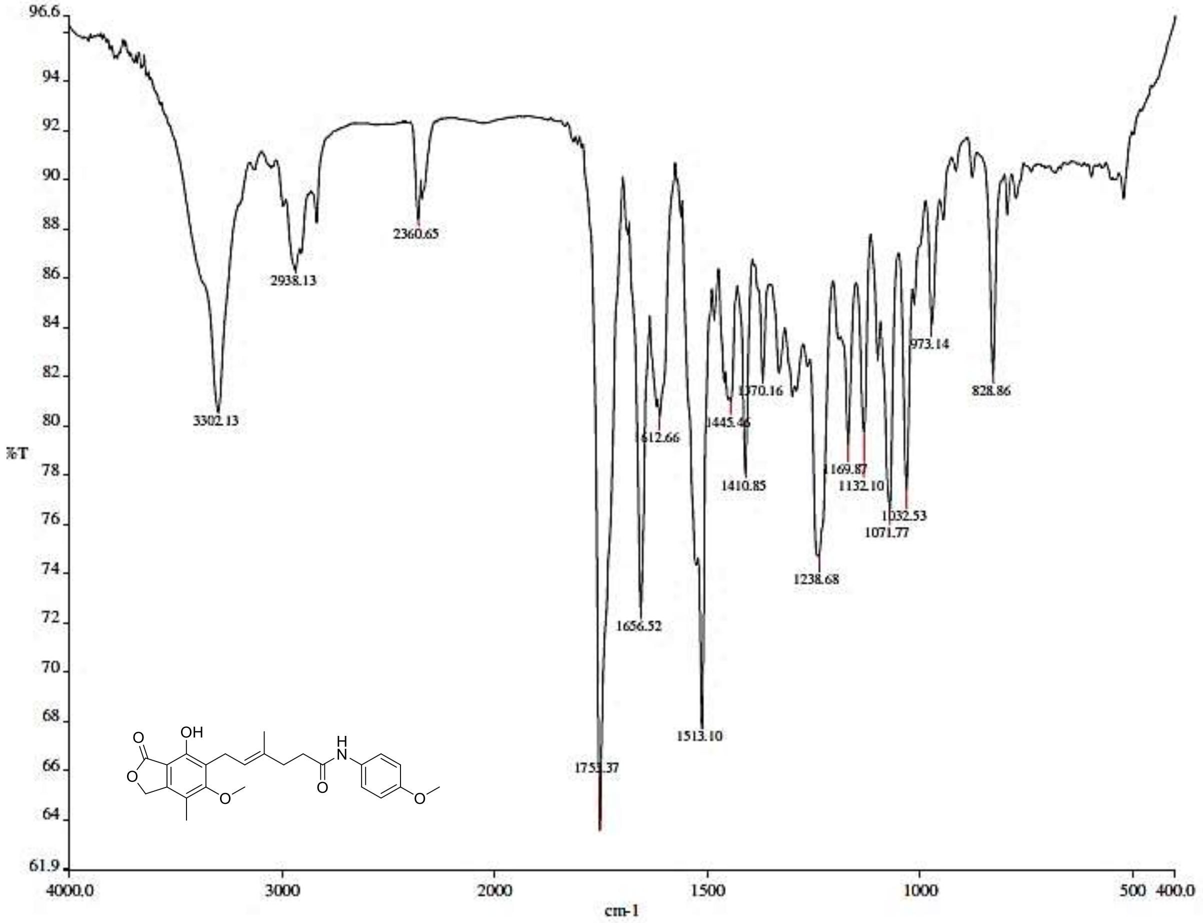


**Figure 59S.** FT-IR spectrum of compound **28**

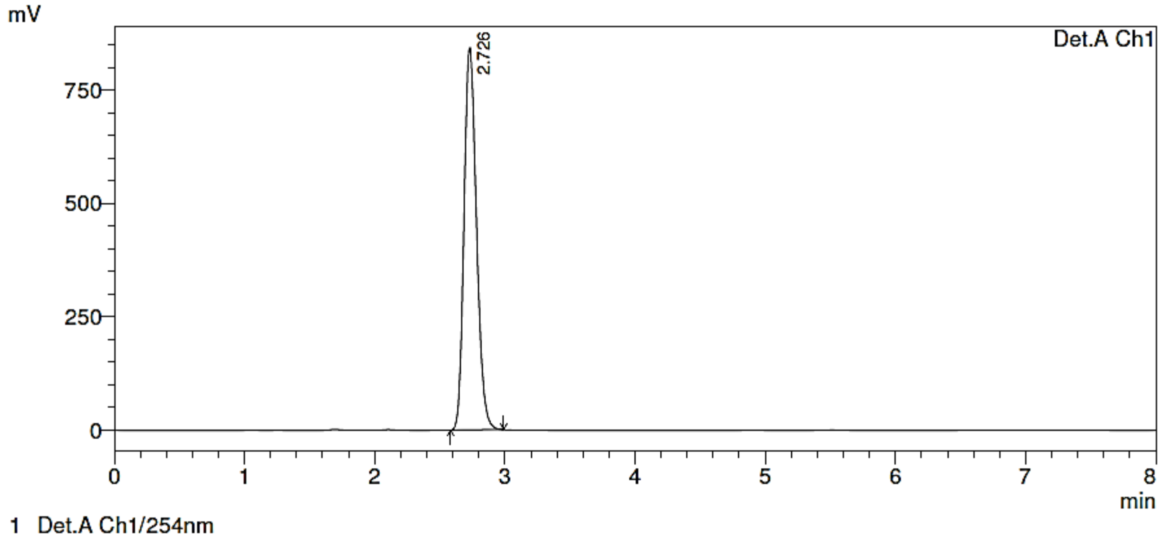


PeakTable

Detector A Ch1 254nm

| Peak# | Ret. Time | Area | Height | Area % | Height % |
|---|---|---|---|---|---|
| 1 | 2.726 | 5610588 | 842404 | 100.000 | 100.000 |
| Total | | 5610588 | 842404 | 100.000 | 100.000 |

Ac

**Figure 60S.** HPLC chromatogram of compound **28**
